# MMA-induced LOXL2^+^ PSCs promote linear ECM alignment in the aging pancreas leading to pancreatic cancer progression

**DOI:** 10.1038/s41419-025-07751-5

**Published:** 2025-05-27

**Authors:** Wenyuan Shi, Haodong Tang, Siyuan Tan, Lishan Wang, Zeqian Yu, Shan Gao, Jiahua Zhou

**Affiliations:** 1 Department of Hepato-Pancreatico-Biliary Surgery, Zhongda Hospital Southeast University, Nanjing, China; 2 Department of Surgery, School of Medicine, Southeast University, Nanjing, China; 3 Zhongda Hospital, School of Life Sciences and Technology, Advanced Institute for Life and Health, Southeast University, Nanjing, China

**Keywords:** Cancer microenvironment, Cancer microenvironment

## Abstract

Pancreatic ductal adenocarcinoma (PDAC) is an age-associated malignancy closely linked to the extracellular matrix (ECM). However, the impact of age-related ECM changes in the normal pancreas on PDAC progression remains unclear. Here, we find that increased linear ECM alignment in normal pancreatic tissues from aged PDAC patients is associated with PDAC progression and worse outcomes. Furthermore, serum methylmalonic acid (MMA) levels are elevated in aged PDAC patients and associated with increased linear ECM alignment in normal pancreatic tissues of PDAC patients. Functionally, MMA promotes LOXL2 expression in pancreatic stellate cells (PSCs), increases linear ECM alignment in normal pancreatic tissues, and facilitates tumor progression. Mechanistically, MMA upregulates KLF10, which forms a transcriptional complex with SP1 to enhance LOXL2 expression in PSCs. Our study demonstrates the role of MMA-induced LOXL2^+^PSCs in ECM remodeling, thus serving as a potential therapeutic target to mitigate PDAC progression in aged patients.

**Figure:**
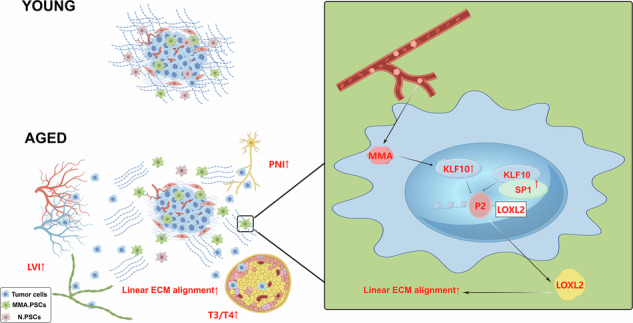
**Schematic diagram showing the molecular mechanism by which MMA-induced LOXL**^**+**^**PSCs promote PDAC progression in the aging pancreas**. In aged individuals, elevated levels of MMA in the blood induce the activation of the KLF10/SP1‒LOXL2 axis in PSCs to increase linear ECM alignment. Following the initiation of pancreatic cancer, this increased linear ECM alignment leads to increased tumor invasion into surrounding tissues, resulting in a greater proportion of stage T3/T4 tumors and a greater incidence of LVI and PNI in aged patients, ultimately leading to poorer outcomes (This schematic was created with www.figdraw.com,export id: PRPRS4e268).

**Schematic diagram showing the molecular mechanism by which MMA-induced LOXL**^**+**^**PSCs promote PDAC progression in the aging pancreas**. In aged individuals, elevated levels of MMA in the blood induce the activation of the KLF10/SP1‒LOXL2 axis in PSCs to increase linear ECM alignment. Following the initiation of pancreatic cancer, this increased linear ECM alignment leads to increased tumor invasion into surrounding tissues, resulting in a greater proportion of stage T3/T4 tumors and a greater incidence of LVI and PNI in aged patients, ultimately leading to poorer outcomes (This schematic was created with www.figdraw.com,export id: PRPRS4e268).

## Introduction

Pancreatic ductal adenocarcinoma (PDAC) is an age-associated malignancy [1, 2]. According to the World Health Organization, by 2050, the percentage of the global population aged 60 and above will increase from 12% to 22% and exceed 2 billion people [3]. This aged population poses substantial challenges for the diagnosis and treatment of pancreatic cancer. Therefore, the impact of aging on pancreatic cancer progression is imperative to be explored.

The extracellular matrix (ECM) is a noncellular component of tissues and can regulate tumor progression [4, 5]. The linear alignment of the ECM increases with increased structural protein alignment [6]. Studies have shown that increased linear ECM alignment in regions near the skin and lymph nodes in aged individuals accelerates melanoma invasion and metastasis to distant organs [7, 8]. However, whether linear ECM alignment is altered in the aging pancreas and whether such alterations are related to PDAC progression are unclear.

Pancreatic stellate cells (PSCs) are stromal cells and can synthesize large amounts of ECM components, such as collagen, fibronectin, laminin, and hyaluronic acid, thereby remodeling the ECM and influencing tumor progression [9, 10]. However, the impact of PSCs on linear ECM alignment in the aging pancreas remains unknown.

In this study, we identify MMA-induced LOXL2^+^ PSCs as key mediators of age-related ECM remodeling in the pancreas, promoting PDAC progression. We show that elevated serum MMA in aged PDAC patients increases linear ECM alignment by upregulating LOXL2 expression in PSCs via KLF10/SP1. Our findings highlight MMA-induced LOXL2^+^ PSCs as a potential therapeutic target to mitigate PDAC progression in aged patients.

## Materials and methods

### Collection and analysis of patient data from the SEER database

Pancreatic cancer patients were identified in the SEER database according to the criteria outlined in Table S1, with 14,242 patients ultimately identified. The detailed patient information is provided in Table S2.

### Patient tissue and serum samples

A total of 100 pancreatic cancer tissue samples were collected from patients at Zhongda Hospital, Southeast University, Nanjing, Jiangsu Province, China, with serum samples also obtained from 40 of these patients. All corresponding patients were pathologically diagnosed with PDAC postsurgery and provided informed consent. The clinical, pathological, and follow-up data for all patients were complete, and no patient had received preoperative radiotherapy or chemotherapy. The detailed patient information is provided in Table S3.

The study was approved by the Ethical Committee of Zhongda Hospital, Southeast University (2016ZDSYLL027.0).

### Culture of PDAC cells and immortalized human PSCs (HPSCs)

The human PDAC cell lines MIA PaCa-2 and Capan-2 were obtained from the Shanghai Cell Bank of the Chinese Academy of Sciences (Shanghai, China). Immortalized HPSCs were purchased from Fenghbio Biological Company (Changsha, China) [11]. All the cells were cultured in a humidified atmosphere at 37 °C with 95% air and 5% CO_2_. PDAC cells were maintained in complete DMEM (Gibco, USA) supplemented with 10% fetal bovine serum (FBS), 100 U/mL penicillin, and 100 μg/mL streptomycin. HPSCs were cultured in complete RPMI-1640 medium (Gibco, USA) supplemented with 10% FBS, 100 U/mL penicillin, and 100 μg/mL streptomycin. HPSCs were cultured for three consecutive days, with treatment 5 mM methylmalonic acid (MMA) (Sigma, GER, catalog number: M54058), 5 mM quinolinate (QA) (Sigma, GER, catalog number: P63204), 5 mM phosphoenolpyruvate (PEP) (Sigma, GER, catalog number: 10108294001) or LOXL2 enzyme inhibitor (LOXL2i) [(2-Chloropyridin-4-yl)methanamine hydrochloride](Medchemexpress, USA, catalog number:HY-101771A) (1 μM). PDAC cells were cultured for five consecutive days and treated with 5 mM MMA.

### Isolation and culture of primary human PSCs

Primary HPSCs were prepared as previously described [12, 13]. Primary HPSCs were isolated from normal pancreatic tissue obtained from PDAC surgical specimens collected at Zhongda Hospital. The tissue was minced and allowed to adhere to the bottom of a 10 cm culture dish. Three milliliters of complete DMEM containing 20% FBS, 100 U/mL penicillin, and 100 μg/mL streptomycin was added to the dish surrounding the tissue blocks. The medium was changed every 48 h, and non-adherent tissue debris was gently removed. Primary HPSCs proliferated outward from the tissue, and cells were passaged when they reached 70–80% confluence. Cell viability was assessed by observing cell attachment and morphology under a microscope. Only cells with good attachment, a typical spindle shape, and no significant floating debris were used for further experiments. Cells between passages 2 and 5 were used for subsequent experiments. Primary HPSCs were identified by immunofluorescence staining for alpha smooth muscle actin (αSMA). Cells were cultured in a humidified atmosphere at 37 °C with 95% air and 5% CO_2_ in complete DMEM supplemented with 20% FBS, 100 U/mL penicillin, and 100 μg/mL streptomycin.

### Immunofluorescence staining (IF)

The tissue samples used were paraffin-embedded sections. After deparaffinization and rehydration, antigen retrieval was performed. The cell-derived extracellular matrix (CDM) samples were fixed with 4% paraformaldehyde (PFA) at room temperature for 30 min. All the samples were then blocked for 30 min with an IF blocking solution containing 0.2% Triton X-100 and 5% bovine serum albumin (BSA). After blocking, the samples were washed with PBS and incubated overnight at 4 °C. The next day, the samples were washed with PBS prior to incubation with secondary antibodies at room temperature for 1 h. After the samples were washed with PBS, the nuclei were stained with 4′,6-diamidino-2-phenylindole (DAPI). The samples were mounted with antifade mounting medium (Servicebio, China, catalog number: G1401), and images were acquired with a Nikon Eclipse C1 microscope (Nikon, Japan). Information about the primary and secondary antibodies is provided in Table S4.

### Measurement of linear alignment of the ECM

Linear alignment of the ECM was measured with the “Orientation” plugin in ImageJ software (RRID:SCR_003070), which generates three indicators to assess linear ECM alignment: light map, peak map, and high-maximum peak frequency (MPF) [8, 14].

### Western blotting (WB)

Protein extraction from tissue samples was performed using the RNA/DNA/Protein Extraction Kit (Beyotime, China; catalog number: R0019M) in accordance with the manufacturer’s instructions. The cells were lysed with radioimmunoprecipitation assay (RIPA) lysis buffer (Beyotime, China; catalog number: P0013B) supplemented with protease inhibitors (Beyotime, China; catalog number: P1005) to extract total cellular protein. Proteins were separated by sodium dodecyl sulfate–polyacrylamide gel electrophoresis (SDS‒PAGE) and transferred to polyvinylidene fluoride (PVDF) membranes (Millipore, Germany). After being blocked with 5% nonfat milk, the membranes were incubated with primary and secondary antibodies. Protein expression was visualized with the SuperSignal™ West Pico PLUS Chemiluminescent Substrate Kit (Beyotime, China; catalog number: P0018AS) and an iBright CL1500 imaging system (Thermo Fisher Scientific, USA), and relative quantification was performed via ImageJ software. Information about the primary and secondary antibodies is provided in Table S4.

### Reverse transcription–quantitative PCR (RT-qPCR)

RNA extraction from tissue and cell samples was performed using the RNA/DNA/Protein Extraction Kit (Beyotime, China; catalog number: R0019M) in accordance with the manufacturer’s instructions. The RNA was reverse transcribed with a reverse transcription kit (Servicebio, China; catalog number: G333). qPCR was performed with a 2× SYBR Green qPCR Master Mix (High ROX) Kit (Servicebio, China; catalog number: G3333-50). The thermal cycling conditions and parameters used for qPCR were selected in accordance with the manufacturer’s instructions. The PCR primers used are listed in Table S5.

### Enzyme-linked immunosorbent assay (ELISA)

The MMA concentration in the serum was measured with an MMA ELISA kit (MyBioSource Inc., USA; catalog number: MBS288266). The assay was performed in accordance with the manufacturer’s instructions.

### CDM assay

CDM preparation was performed as described previously [8, 15]. Twelve-millimeter coverslips were placed in the wells of a 24-well plate and coated with 0.2% gelatin solution at room temperature for 1 h. The coverslips were then washed with PBS and incubated with 1% glutaraldehyde solution at room temperature for 30 min. After washing with PBS, the coverslips were incubated with 1 M ethanolamine at room temperature for 30 min. After five washes with PBS, 1.0 × 10^5^ PSCs were seeded into each well, and the plate was incubated overnight at 37 °C in 5% CO_2_. The medium was then replaced with complete DMEM containing 50 μg/mL ascorbic acid, with or without LOXL2i (1 μM). The medium was replaced every other day thereafter with fresh medium containing 50 μg/mL ascorbic acid, where applicable, LOXL2i. After five treatments, ECM formation was complete, and the PSCs were removed from the ECM. The wells were washed twice with PBS, treated with 200 μL of buffer solution (PBS containing 0.05% Triton X-100 and 20 mM NH_4_OH) for 10 min, and incubated overnight at 4 °C with an additional equal volume of PBS. The next day, the wells were washed multiple times with PBS to remove cell debris. Finally, the ECM was subjected to IF staining for type I collagen (COL I) to measure linear ECM alignment.

### CCK-8 assay

Cells in the logarithmic growth phase were used for this assay. The cells were seeded in a 96-well plate (5000 cells per well), with five replicates established per group. After 24 h of routine culture, the cells were treated with different concentrations of MMA (0 mM, 1.25 mM, 2.5 mM, 5 mM, or 10 mM). Cell viability was measured daily for 3 days. Tumor cells were treated with either 0 mM or 5 mM MMA, and their viability was measured daily for 5 days. For each measurement, the original medium was removed, and 100 μL of CCK-8 reagent (prepared according to the manufacturer’s instructions; Servicebio, China; catalog number: G4103) was added to each well. After 2 h of incubation, the absorbance was measured at 450 nm with a microplate reader. A no-cell control (NOC) well was included in the experiment.

### EdU assay

Cells were treated with 0.5% Triton X-100 and incubated for 10 min. Subsequently, 100 µL of the EdU staining reaction mixture, as per the instructions of the Click-iT EdU-488 Cell Proliferation Detection Kit (Servicebio, China; catalog number: G1601). Afterward, DAPI staining solution was added dropwise, followed by a 10 min incubation. The percentage of green fluorescent cells was then determined via fluorescence microscopy.

### RNA isolation and library preparation

After 3 days of induction with 5 mM MMA (n = 4) or the control treatment (n = 4), RNA-seq was conducted. Total RNA from PSCs induced with MMA (MMA.HPSCs) and the corresponding control PSCs (N.HPSCs) was extracted with TRIzol reagent (Invitrogen, USA) according to the manufacturer’s instructions. RNA purity and quantity were assessed with a NanoDrop 2000 spectrophotometer (Thermo Fisher Scientific, USA), and RNA integrity was evaluated with an Agilent 2100 Bioanalyzer (Agilent Technologies, Santa Clara, CA, USA). Transcriptome libraries were prepared with the VAHTS Universal V5 RNA-seq Library Prep Kit following the manufacturer’s instructions. Transcriptome sequencing and analysis were performed by OE Biotech Co., Ltd. (Shanghai, China).

### RNA-seq and data analysis

RNA-seq was performed by OE Biotech Co., Ltd. (Shanghai, China). Libraries were sequenced on the Illumina NovaSeq 6000 platform, generating 150 bp paired-end reads. To obtain clean reads for subsequent analysis, the raw reads in FASTQ format were processed with the fastp software to remove low-quality reads. HISAT2 was used to align the clean reads to the reference genome, and gene expression levels (in FPKM format) were calculated. The htseq-count script was used to obtain the read count for each gene. Principal component analysis (PCA) and plotting were performed with R (v3.2.0) to evaluate biological replicates. Differentially expressed genes (DEGs) were identified via DESeq2, with genes meeting the criteria of a q-value < 0.05 and a |log2FC | >1 considered as DEGs. Hierarchical clustering analysis of the DEGs was conducted to identify gene expression patterns across different groups and samples. Volcano plots of the top 30 DEGs were generated via the ggradar R package to visualize changes in gene expression. Functional enrichment analyses were performed via databases such as Gene Ontology (GO), Kyoto Encyclopedia of Genes and Genomes (KEGG) Pathway, Reactome, and WikiPathways, with differential enrichment determined on the basis of the hypergeometric distribution, and visualizations were generated via R (v3.2.0). Gene set enrichment analysis (GSEA) was performed via GSEA software with predefined gene sets to test whether these gene sets were significantly enriched or depleted (at the top or bottom of the ranking table, respectively) on the basis of differential gene expression.

### PDAC cell line–PSC subcutaneous cotransplantation model

All animal experiments were approved by the Institutional Animal Care and Use Committee of Southeast University (20230306002). Subcutaneous cotransplantation of PDAC cell lines and PSCs was performed as previously described [16]. A mixture of 1.5 × 10^6^ PSCs and 5 × 10^5^ PDAC cells in 200 µL of phenol red-free Matrigel was injected subcutaneously into 8-week-old BALB/c nude mice (Vital River, China). The mice were divided into groups with equal numbers of male and female mice in each group. After 14 days, the mice were sacrificed, and the tumors were fixed with 4% PFA. For LOXL2i treatment, PDAC cells with N.HPSCs or MMA.HPSCs were subcutaneously injected, and starting from day 6, LOXL2i (30 mg/kg) was administered intraperitoneally every day for 8 consecutive days. The control group was treated with saline. On day 14, the mice were sacrificed, and the tumors were excised for subsequent experiments.

### Immunohistochemistry (IHC)

The paraffin-embedded specimens were placed in a heated incubator for 20 min. Next, the paraffin sections were successively immersed in xylene to remove the paraffin, followed by dehydration using absolute ethanol and a 90% ethanol-water mixture. Subsequently, the sections were incubated in 3% H2O2 at room temperature, protected from light, for 25 min to eliminate endogenous peroxidase activity. The sections were then blocked with BSA blocking solution at room temperature for 1 h, followed by overnight incubation with the primary antibody at 4 °C. The next day, the sections were incubated with the corresponding secondary antibody. Positive staining was visualized using DAB (3,3′-diaminobenzidine) (Servicebio, China; catalog number: G1212), and then the sections were counterstained with hematoxylin. Finally, the sections were dehydrated and mounted with a coverslip. Finally, take photos and analyze with ImageJ.

### Lentiviral vector construction and transduction

The vector-LOXL2, OE-LOXL2, sh1-LOXL2, sh2-LOXL2, and sh3-LOXL2 lentiviral vectors were constructed by GenePharma (Shanghai, China). When PSCs reached 70% confluence in a 6-well plate, they were infected with lentiviruses. Cells selected with puromycin that remained viable in the presence of 10 μg/ml puromycin were determined to be successfully transduced. The transduction efficiency was validated by WB and qPCR analyses.

### Single-cell RNA-seq analysis

Data were downloaded from the Gene Expression Omnibus (GEO) database (GSE84133). The data were processed and analyzed via the R packages limma, Seurat, dplyr, magrittr, celldex, SingleR, and monocle (R v4.3.2).

### Plasmid construction and transfection

The overexpression plasmids for PBX3, E2F1, ELF3, KLF10, and SP1 (pcDNA-PBX3, pcDNA-E2F1, pcDNA-ELF3, pcDNA-KLF10, and pcDNA-SP1, respectively) were constructed by GenePharma (Shanghai, China). Plasmid transfection was performed with Lipofectamine 3000 (Thermo Fisher Scientific, USA, catalog number: L3000008) according to the manufacturer’s instructions. The transfection efficiency and downstream molecule expression levels were verified by WB and qPCR analyses 48–72 h posttransfection.

### Chromatin immunoprecipitation (CHIP)

ChIP was performed with a Pierce Magnetic ChIP Kit (Thermo Fisher Scientific, USA; catalog number: 26157) following the manufacturer’s protocol. After the DNA fragments were obtained, qPCR was used to assess the target promoters. The PCR primers used are listed in Table S5. Information about the primary antibodies is provided in Table S4.

### Dual-luciferase reporter assay

The firefly luciferase plasmids, including the empty vector, plasmids containing the wild-type promoter, plasmids containing a point mutation in either P1 or P2, and plasmids containing point mutations in both P1 and P2 (pGV238-EV, pGV238-WT, pGV238-MutP1, pGV238-MutP2, and pGV238-MutP1&P2, respectively), and the Renilla luciferase plasmid (pGV238-TK), were constructed by Genechem (Shanghai, China). Plasmid transfection was performed with Lipofectamine 3000 (Thermo Fisher Scientific, USA; catalog number: L3000008) following the manufacturer’s instructions. After 48 h of transfection, luciferase activity was measured with a Dual-Luciferase Reporter Assay Kit (Beyotime Biotechnology, China; catalog number: RG027).

### siRNA construction and transfection

Three siRNAs targeting KLF10 were constructed by GenePharma (Shanghai, China). The siRNA sequences are listed in Table S6. siRNA transfection was performed with Lipofectamine 3000 (Thermo Fisher Scientific, USA; catalog number: L3000008) according to the manufacturer’s instructions. The transfection efficiency and downstream molecule expression levels were verified by WB and qPCR analyses 48–72 h posttransfection.

### Co-immunoprecipitation (Co-IP)

Co-IP was performed with an immunoprecipitation kit (Beyotime Biotechnology, China; catalog number: P2179S) according to the manufacturer’s instructions. Information about the primary antibodies is provided in Table S4.

### Statistical analysis

The data were statistically analyzed via SPSS 26.0 software. Independent sample t tests were used for comparisons between two groups, whereas one-way analysis of variance (ANOVA) was used for comparisons among more than two groups. Pearson correlation analysis was applied to assess correlations between categorical variables. Cox univariate and multivariate analyses, Kaplan‒Meier (KM) survival analysis, and the chi‒square test were performed with R (v4.3.2). Graphs were generated via GraphPad Prism 8. Continuous data are presented as the means ± standard deviations. The statistical differences between comparison groups are indicated by black horizontal lines with asterisks on the bar graphs. A p-value < 0.05 was considered to indicate statistical significance (*, p < 0.05; **, p < 0.01; and ***, p < 0.001). All experiments were conducted with a minimum of three replicates.

## Results

### Increased linear ECM alignment in the aged pancreas is associated with PDAC progression

To investigate whether PDAC prognosis differs based on patient age, we screened 14,242 PDAC patients from the SEER database and categorized them into aged (≥65 years) and young (<65 years) groups and confirmed poorer overall survival (OS) in aged PDAC patients (Fig. S1A, B). Furthermore, the aged group had greater proportions of poorly differentiated tumors and stage T3/T4 tumors (Fig. S1C). In a separate cohort of 100 PDAC patients from Zhongda Hospital, 50 aged (≥65 years) and 50 young patients (<65 years) were analyzed. Consistent with the above findings, aged PDAC patients in this cohort had shorter OS (Fig. S1D). Comparisons between the aged and young groups revealed that the aged group had greater proportions of poorly differentiated tumors, stage T3/T4 tumors, lymphovascular invasion (LVI), and perineural invasion (PNI) (Fig. S1E). Therefore, these results suggest that age is an independent factor for poor prognosis in PDAC.

**Fig. 1:**
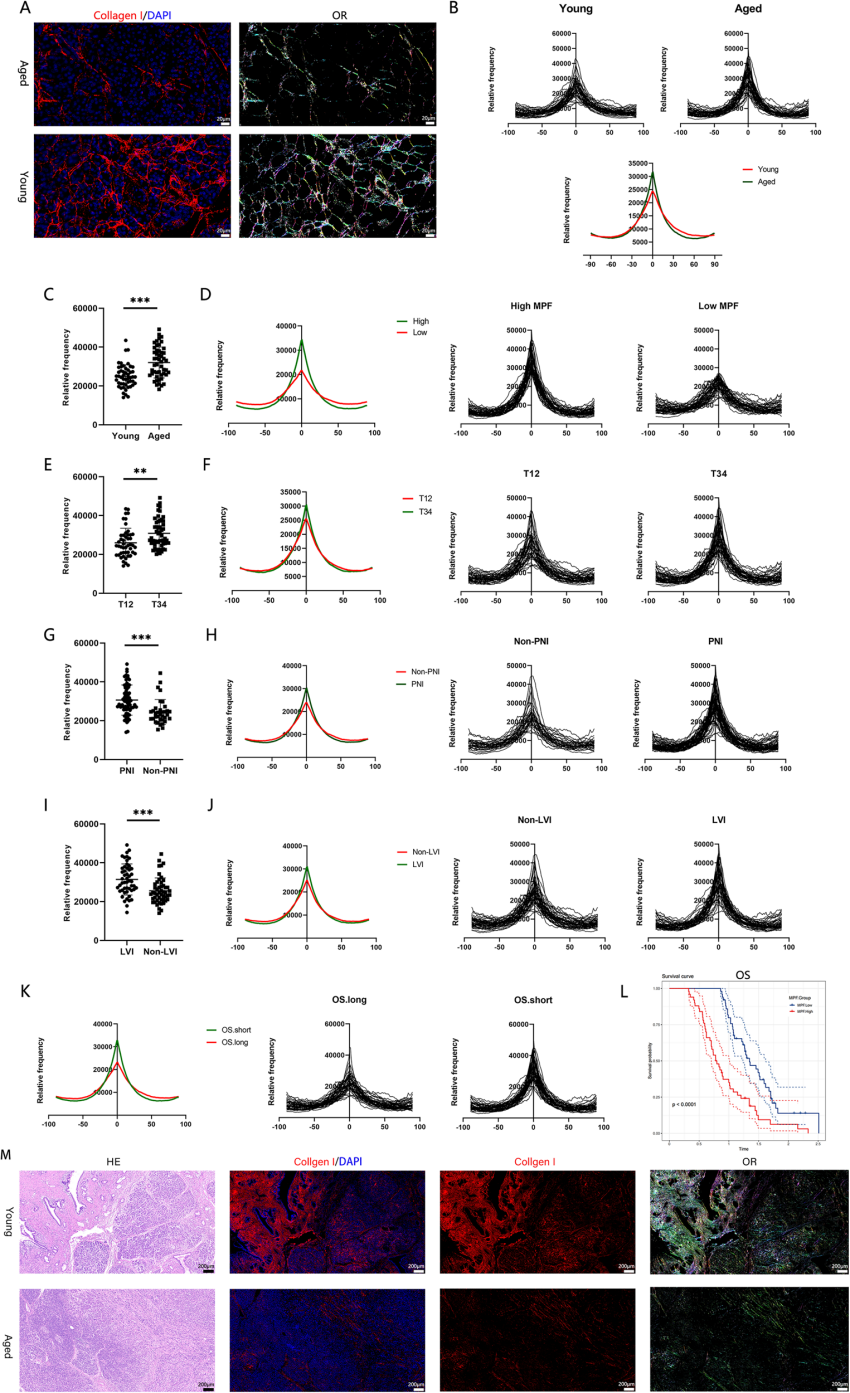
Increased linear ECM alignment in the aged pancreas is associated with PDAC progression. Linear ECM alignment mapping after IF staining for COL I in normal pancreatic tissue (**A**–**C**). **A** Light map of linear ECM alignment. The more colors there are, the weaker the linear alignment. **B** Peak map of linear ECM alignment. **C** MPF data indicating linear ECM alignment. **D** Comparison of linear ECM alignment between the high-MPF and low-MPF groups. Analysis of pathological information related to the tumor tissues corresponding to the normal pancreatic tissues (**E**–**J**). **E**, **F** Comparison of linear ECM alignment between the T1/T2 and T3/T4 groups. **G**, **H** Comparison of linear ECM alignment between the PNI-positive and PNI-negative groups. **I**, **J** Comparison of linear ECM alignment between the LVI-positive and LVI-negative groups. **K** Comparison of linear ECM alignment between the short-term and long-term survival groups. **L** OS of the MPF.High and MPF.Low groups. **M** Representation of pancreatic cancer invading normal pancreatic tissue. (*p < 0.05, **p < 0.01, ***p < 0.001).

We analyzed normal tumor-adjacent pancreatic tissues from PDAC patients in paraffin-embedded samples to investigate differences in linear ECM alignment between aged and young individuals, which revealed that pancreatic tissues from aged individuals presented increased linear ECM alignment (Fig. 1A–C). The patients were then subdivided into MPF and low-MPF groups based on the median MPF value, with a higher linear ECM alignment observed in the high-MPF group (Fig. 1D). We analyzed the pathological information of the tumor tissues corresponding to these adjacent normal pancreatic tissues. Tumors in the T3/4 group (Fig. 1E, F), PNI-positive group (Fig. 1G, H), and LVI-positive group (Fig. 1I, J) exhibited greater linear ECM alignment. Furthermore, patients with shorter survival times had greater linear ECM alignment (Fig. 1K), and those in the high-MPF group had shorter OS (Fig. 1L). Additionally, we observed that in young patients, the weak linear alignment of pancreatic ECM forms a dense collagen network, protecting normal tissue from tumor invasion. In contrast, an aging pancreas with increased ECM alignment is more susceptible to tumor invasion (Fig. 1M). Therefore, these results indicate that in aged patients, increased linear ECM alignment in pancreatic tissue is associated with PDAC progression.

### MMA associates linear ECM alignment in aged pancreas and PDAC progression

**Fig. 2:**
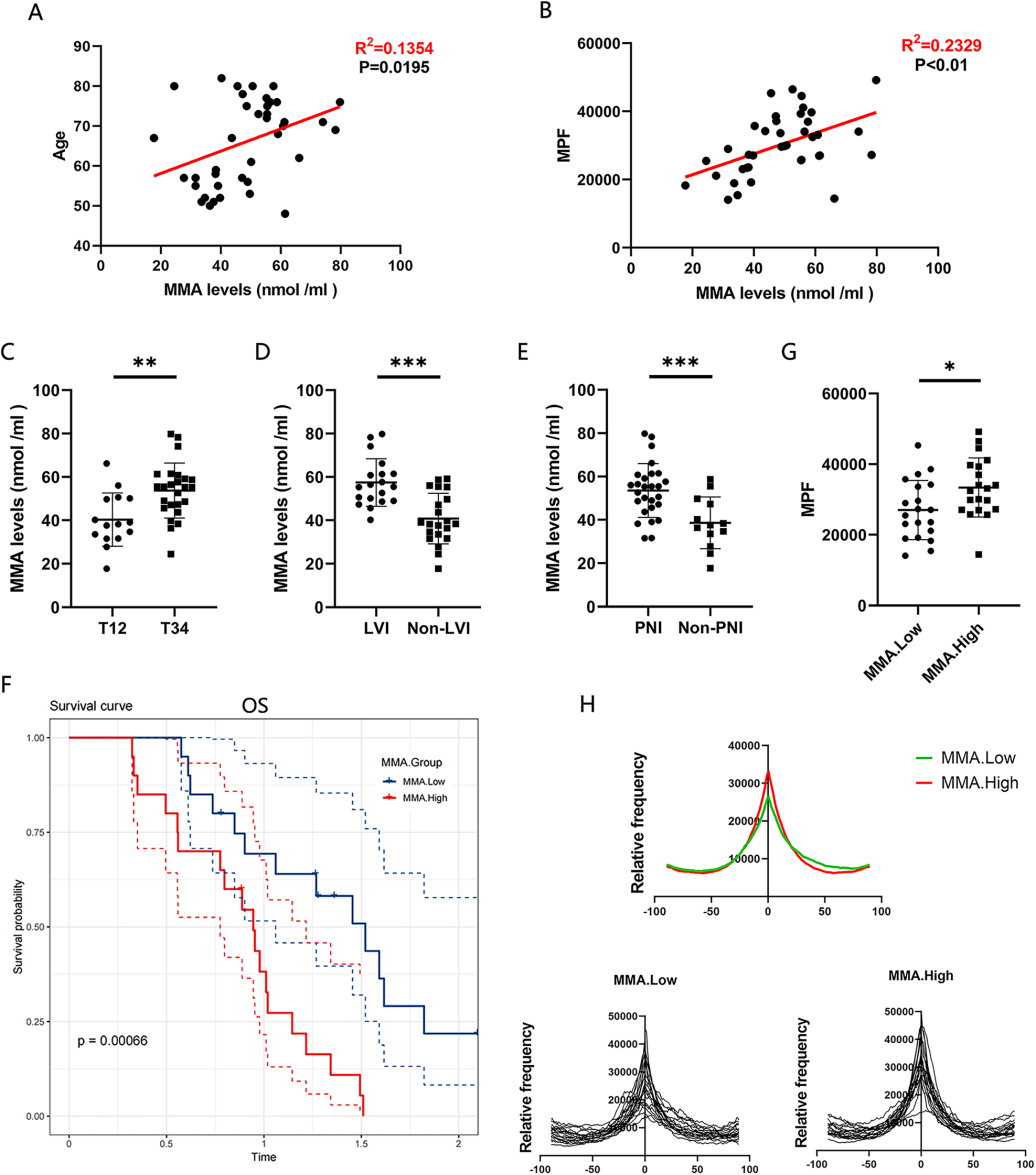
MMA associates linear ECM alignment in aged pancreas and PDAC progression. **A** Correlation analysis between serum MMA levels and age in PDAC patients. **B** Correlation analysis between serum MMA levels in PDAC patients and linear ECM alignment in normal pancreatic tissues. **C** Comparison of serum MMA levels between the T1/T2 and T3/T4 groups. **D** Comparison of serum MMA levels between the LVI-positive and LVI-negative groups. **E** Comparison of serum MMA levels between the PNI-positive and PNI-negative groups. **F** OS of the MMA.High and MMA.Low groups. **G**, **H** Comparison of linear ECM alignment between the MMA.High and MMA.Low groups. (*p < 0.05, **p < 0.01, ***p < 0.001).

A previous study revealed that the level of methylmalonic acid (MMA) in the blood is greater in aged individuals than in young individuals [17]. Serum MMA levels were measured in 40 PDAC patients, revealing that serum MMA levels were positively correlated not only with age (Fig. 2A), but also with linear ECM alignment in normal pancreatic tissues (Fig. 2B). Additionally, serum MMA levels were significantly higher in the T3/4 group (Fig. 2C), LVI-positive group (Fig. 2D), and PNI-positive group (Fig. 2E) among PDAC patients. 40 patients were further subdivided into MMA.High and MMA.Low groups based on the median MMA concentrations. Patients in the MMA.High group exhibited shorter OS (Fig. 2F) and stronger linear ECM alignment in normal pancreatic tissues (Fig. 2G, H) compared to those in the MMA.Low group. These findings suggest that MMA is associated with linear ECM alignment in aged pancreas and with PDAC progression.

### MMA induces LOXL2 expression in PSCs to increase linear ECM alignment

Various molecules, including lysyl oxidase (LOX), lysyl oxidase-like 2 (LOXL2), cellular communication network factor 4 (CCN4), and procollagen-lysine, 2-oxoglutarate 5-dioxygenase 2 (PLOD2), can increase linear ECM alignment [3]. We analyzed the expression of the genes in different normal pancreatic cell lines via the UCSC Cell Browser scRNA-seq dataset. The results revealed greater expression of LOX, LOXL2, PLOD2, and CCN4 in PSCs than in other pancreatic cells (Fig. S2A–D), suggesting that PSCs have the potential to remodel linear ECM alignment.

**Fig. 3:**
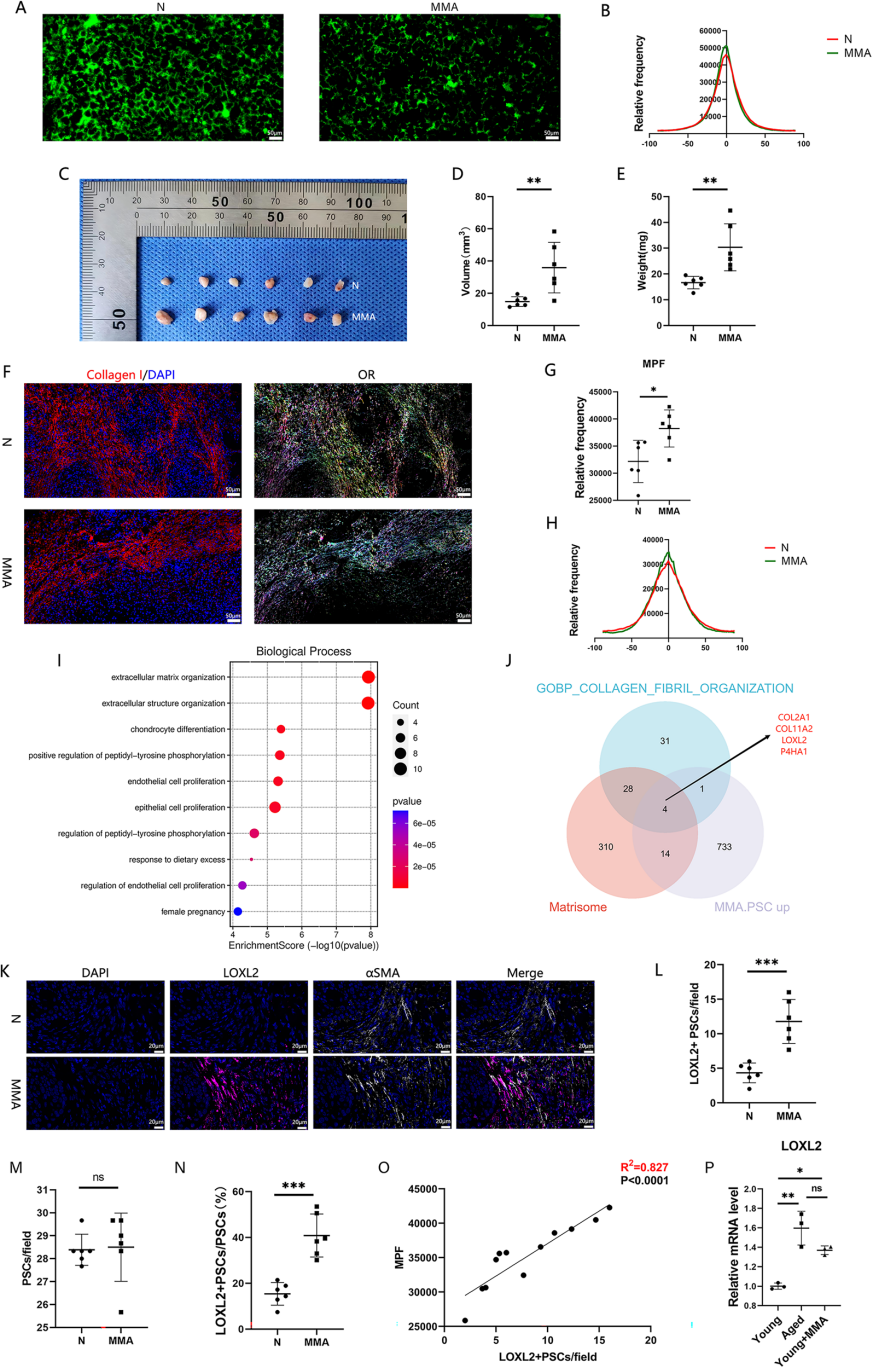
MMA induces LOXL2 expression in PSCs to increase linear ECM alignment. **A** ECM production in MMA.HPSCs and control HPSCs (n = 3 biological replicates). **B** Comparison of linear ECM alignment as measured in (**A**). **C**–**E** Comparison of tumor volume and weight between MMA.HPSC+MIA PaCa-2 tumors and N.HPSC+MIA PaCa-2 tumors (n = 6 mice per group). **F**–**H** Comparison of linear ECM alignment between MMA.HPSC+MIA PaCa-2 tumors and N.HPSC+MIA PaCa-2 tumors. Linear ECM alignment in tumor tissues was assessed via IF staining for COL I (n = 6 mice per group). **I** GO analysis of secretory molecules upregulated in MMA-HPSCs. **J** Identification of overlapping genes among genes whose expression was upregulated in MMA.HPSCs, matrisome genes, and genes in the GOBP_COLLAGEN_FIBRIL_ORGANIZATION gene set. **K** Multiplex IF staining of MMA.HPSC + MIA PaCa-2 tumors and N.HPSC+MIA PaCa-2 tumors. αSMA (white), LOXL2 (pink) (n = 6 mice per group). **L** Number of double-stained cells (LOXL2^+^PSCs) in (**K**). **M** Number of αSMA-stained cells (PSCs) in (**K**). **N** The proportion of LOXL2^+^ PSCs among the total PSCs in (**K**). **O** Correlation analysis between tumor linear ECM alignment and the infiltration of LOXL2^+^ PSCs in subcutaneous coimplantation experiments. **P** qPCR analysis of LOXL2 expression in the Old.PSC, Young.PSC and MMA. Young.PSC groups (n = 3 biological replicates). (*p < 0.05, **p < 0.01, ***p < 0.001).

The potential of MMA to induce remodel linear ECM alignment in PSCs was subsequently investigated. 5 mM MMA treatment for 3 days caused no cytotoxicity (Fig. S3A). Therefore, we treated HPSCs with 5 mM MMA for 3 days for subsequent experiments. In CDM assays, HPSCs produced an ECM with greater linear alignment than did the control group after stimulation with MMA (Fig. 3A, B). We examined COL I expression in HPSCs after MMA induction and found that while COL1A1 mRNA levels increased, COL1A2 expression remained unchanged (Fig. S3B), and COL I protein levels showed no significant difference (Fig. S3C, D). In subcutaneous coimplantation experiments, tumors formed in the experimental group (MMA.HPSC+MIA PaCa-2 group) were larger and heavier than those in the control group (N.PSCs+MIA PaCa-2 group) (Fig. 3C–E). ECM mapping revealed greater linear ECM alignment in the experimental group (Fig. 3F–H). Notably, although MMA-induced MIA PaCa-2 cells exhibited pro-tumorigenic effects (Fig. S3E–J), they did not enhance linear ECM alignment (Fig. S3K–M). linear ECM alignment remained significantly weaker (Fig. S3K–M), and PSCs were also markedly fewer (Fig. S3N, O) in these tumors compared to those in the N.HPSCs+MIA PaCa-2 and MMA.HPSCs+MIA PaCa-2 groups. Therefore, these results indicate that MMA induces PSCs to increase linear ECM alignment, thereby promoting PDAC progression.

To confirm that the increased linear ECM alignment in aged individuals is driven by MMA-induced PSCs, we isolated primary human PSCs from normal pancreatic tissue obtained from fresh surgical samples (Fig. S4A). CDM assay demonstrated that the Old.PSCs group presented an ECM with greater linear alignment (Fig. S4B, C). The Young. PSCs group presented greater linear ECM alignment under MMA treatment (Fig. S4B, C). These findings indicate that MMA can induce PSCs to increase linear ECM alignment in aged individuals.

To identify molecules involved in remodeling the linear ECM alignment, we conducted RNA-seq analysis of the MMA.HPSCs and uninduced control HPSCs. RNA-seq revealed 2354 DEGs, with 752 upregulated genes and 1602 downregulated genes in MMA.HPSCs (q-value < 0.05 & |log2FC | > 1) (Fig. S5A). Reactome analysis revealed their association with the senescence-associated secretory phenotype (SASP) (Fig. S5B), whereas KEGG analysis revealed that the upregulated genes were associated with aging (Fig. S5C). GO analysis of paracrine protein-encoding genes among the upregulated genes revealed significant enrichment in ECM-related gene sets (Fig. 3I). By assessing the intersection of the genes upregulated in MMA.PSCs, matrisome-related genes, and genes in the Gobp Collagen Fibril Organization gene set, we identified four overlapping genes: LOXL2, COL2A1, COL11A2, and P4HA1(Fig. 3J, Table S7). Among the identified molecules, only LOXL2 possessed lysine oxidase activity, which is essential for collagen crosslinking, and was significantly upregulated upon MMA treatment. In vivo, COL I and αSMA expression remained unchanged in both the N.HPSC+MIA PaCa-2 and MMA.HPSC+MIA PaCa-2 groups, while LOXL2 expression was elevated in the MMA.HPSC+MIA PaCa-2 group (Fig. S5D–G). Multiplex IF staining of tumor tissues from subcutaneous coimplantation experiments revealed increased infiltration of LOXL2^+^ PSCs in the MMA.HPSC+MIA PaCa-2 group (Fig. 3K, L). Although the total number of PSCs was similar between the MMA.HPSC+MIA PaCa-2 and N.HPSC+MIA PaCa-2 groups (Fig. 3M), the proportion of LOXL2^+^ PSCs was higher in the MMA.HPSC+MIA PaCa-2 group (Fig. 3N). Additionally, the number of infiltrating LOXL2^+^ PSCs was positively correlated with the degree of linear ECM alignment (Fig. 3O). Compared with Young.PSCs, Old.PSCs presented higher LOXL2 expression, and the Young.PSCs group presented increased LOXL2 expression under MMA treatment (Fig. 3P). Furthermore, Previous research has reported that, in addition to MMA, both QA and PEP are also elevated in the serum of aged individuals [17]. However, when HPSCs were treated separately with 5 mM QA or 5 mM PEP, neither treatment induced any changes in linear ECM alignment (Fig. S5H, I) or LOXL2 expression (Fig. S5J–L). These findings imply that the elevated LOXL2 expression in MMA-induced PSCs increases linear ECM alignment, thereby promoting tumor progression.

**Fig. 4:**
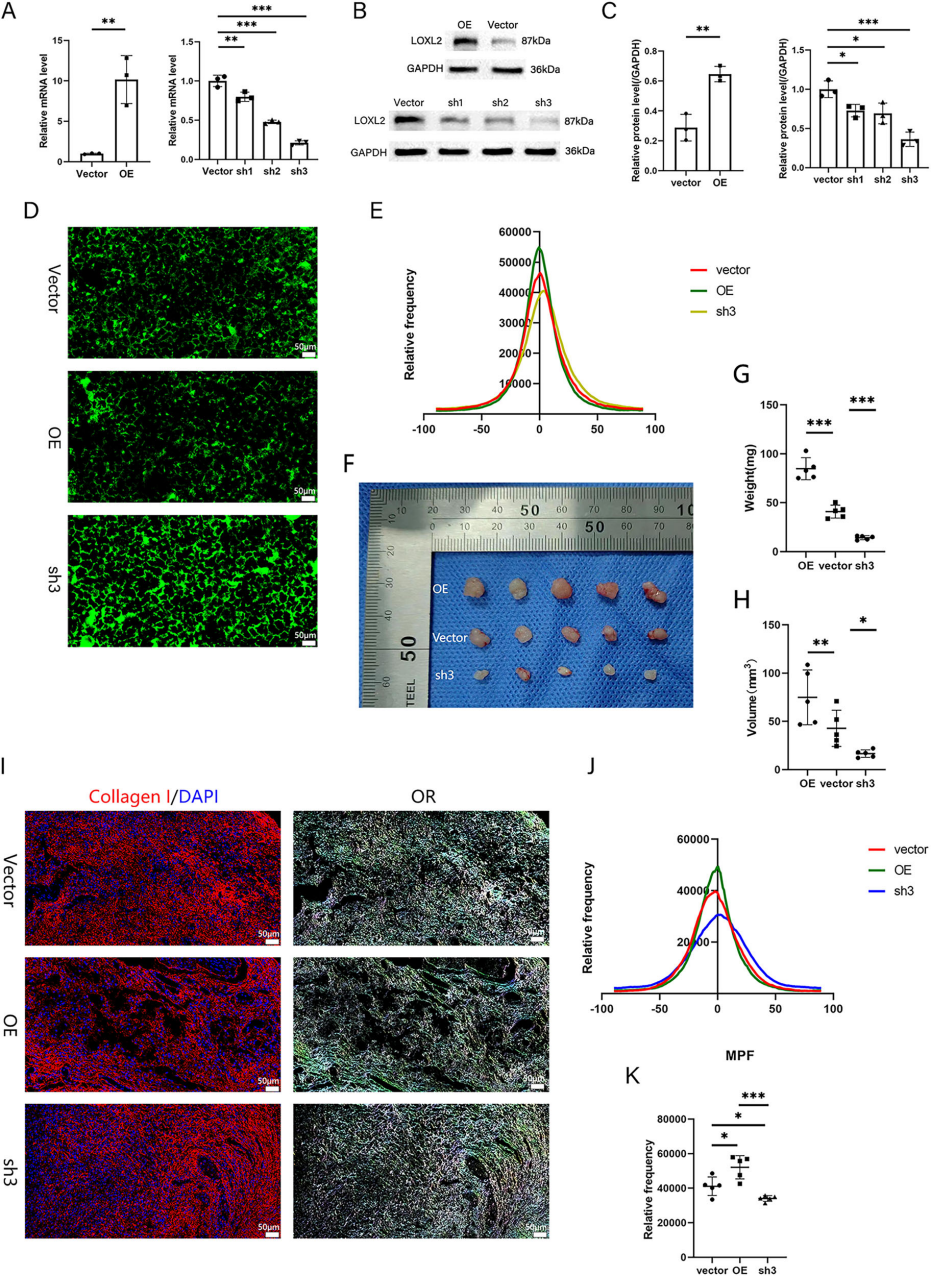
LOXL2 in PSCs increases linear ECM alignment and accelerates PDAC progression. **A** qPCR analysis of LOXL2 expression in the Vector-LOXL2.HPSCs, OE-LOXL2.HPSCs, and sh-LOXL2.HPSCs (n = 3 biological replicates). Representative (**B**) and quantitative (**C**) WB results of LOXL2 expression in Vector-LOXL2.HPSCs, OE-LOXL2.HPSCs, and sh-LOXL2.HPSCs (n = 3 biological replicates). **D** ECM produced by Vector-LOXL2.HPSCs, OE-LOXL2.HPSCs, and sh-LOXL2.HPSCs. **E** Analysis and comparison of linear ECM alignment between Vector-LOXL2.HPSCs, OE-LOXL2.HPSCs, and sh-LOXL2.HPSCs. **F**–**H** Comparison of tumor volume and weight between Vector-LOXL2.HPSC+MIA PaCa-2 tumors, OE-LOXL2.HPSC+MIA PaCa-2 tumors, and sh3-LOXL2.HPSC+MIA PaCa-2 tumors (n = 5 mice per group). **I**–**K** Comparison of linear ECM alignment between Vector-LOXL2.HPSC+MIA PaCa-2 tumors, OE-LOXL2.HPSC+MIA PaCa-2 tumors, and sh3-LOXL2.HPSC + MIA PaCa-2 tumors. Linear ECM alignment in tumor tissues was assessed by IF staining for COL I (n = 5 mice per group). (*p < 0.05, **p < 0.01, ***p < 0.001).

To further validate the role of LOXL2 in the remodeling of linear ECM alignment in PSCs, we established sh-LOXL2.HPSCs and OE-LOXL2.HPSCs (Fig. 4A–C). CDM assays revealed that LOXL2 overexpression in PSCs increased linear ECM alignment compared with that in the corresponding control groups (Fig. 4D, E). Subcutaneous coimplantation experiments further confirmed that, compared with the control group (Vector-LOXL2.HPSCs+MIA PaCa-2 group), the LOXL2-OE group (OE-LOXL2.HPSCs+MIA PaCa-2 group) exhibited significant tumor-promoting effects (Fig. 4F–H), with tumors displaying greater linear ECM alignment (Fig. 4I–K). Compared to the control group, the LOXL2-OE group showed no significant increase in COL I and αSMA expression, while LOXL2 expression was elevated (Fig. S6A–D). Multiplex IF staining of tumor tissues revealed that the total number of PSCs remained unchanged, but the number and the proportion of LOXL2^+^ PSCs were higher in the LOXL2-OE group (Fig. S6E–H). In contrast, sh3-LOXL2.HPSCs produced effects opposite to those observed in OE-LOXL2.HPSCs in both in vitro and in vivo experiments (Fig. 4D–K, Fig. S6A–H). Together, MMA induces LOXL2 expression in PSCs, increasing linear ECM alignment and accelerating tumor progression.

Furthermore, LOXL2 knockdown in HPSCs significantly inhibited MMA-induced LOXL2 upregulation (Fig. S7A, B). Subcutaneous coimplantation experiments further demonstrated that LOXL2 knockdown suppressed the tumor-promoting effects of MMA-induced HPSCs (Fig. S7C–E) and reduced linear ECM alignment (Fig. S7J–L). Compared to the MMA-induced group (MMA.Vector-LOXL2.HPSCs+MIA PaCa-2 group), the LOXL2-KD/MMA group (MMA.sh3-LOXL2.HPSCs+MIA PaCa-2) showed significantly lower LOXL2 expression in tumor tissues, with no significant increase in COL I and αSMA expression (Fig. S7F–I). Multiplex IF staining revealed no statistical difference in overall PSC infiltration among groups, but the number and proportion of LOXL2^+^ PSCs were significantly reduced in the LOXL2-KD/MMA group (Fig. S7M–P), highlighting LOXL2 as a key effector in MMA-induced ECM remodeling.

To further validate these findings and explore clinical translation, we tested the LOXL2 inhibitor [(2-Chloropyridin-4-yl)methanamine hydrochloride]. Although this inhibitor did not affect MMA-induced LOXL2 upregulation (Fig. S8A, B), CDM assays demonstrated that it effectively suppressed MMA-enhanced linear ECM alignment (Fig. S8C, D). In subcutaneous coimplantation experiments, the MMA/LOXL2i-treated group (MMA.HPSCs+MIA PaCa-2+LOXL2i) exhibited reduced tumor-promoting effects (Fig. S8E–G) and weaker linear ECM alignment (Fig. S8J–L) compared to the MMA-induced group (MMA.HPSCs+MIA PaCa-2+saline), while LOXL2 expression remained unchanged between the two groups (Fig. S8H, I). Multiplex IF staining of tumor tissues showed that LOXL2i did not impact MMA-induced LOXL2^+^ PSC infiltration, with no significant difference in total PSC numbers between the MMA-induced group and MMA/LOXL2i-treated group (Fig. S8M–P). Similar results were obtained in vivo using another pancreatic cancer cell line, Capan-2 (Fig. S9A–L). These findings suggest that LOXL2i suppresses MMA-induced linear ECM alignment and tumor progression without altering LOXL2 expression in PSCs. This further confirms LOXL2 as a critical effector in MMA-driven ECM remodeling and supports targeting LOXL2^+^ PSCs as a potential therapeutic strategy.

### LOXL2^+^ PSCs increase linear ECM alignment in the aged pancreas and accelerate PDAC progression

By analyzing the GSE84133 scRNA-seq dataset, PSCs were screened through cluster analysis and further divided into seven clusters. Among them (Fig. S10A, B), Cluster 0 was significantly enriched in inflammatory signaling pathways, including IL-17, TNF, and NF-κB, suggesting its critical role in inflammatory responses and immune regulation. Clusters 1, 4, and 5 were primarily involved in ECM remodeling, whereas Cluster 2 was associated with endocrine regulation, oxidative stress, and vascular function. Cluster 3 was linked to immune response and angiogenesis, while Cluster 6 was mainly responsible for maintaining pancreatic endocrine homeostasis (Tables S8, S9). Notably, in aged pancreatic samples, the proportions of Clusters 1 and 5 were significantly elevated, whereas Cluster 4 was predominantly enriched in young samples (Fig. S10C). Although both Clusters 1 and 5 contributed to ECM remodeling, they exhibited distinct molecular signatures and expression patterns. Cluster 1 primarily exhibited high expression of ECM structural proteins, whereas Cluster 5 not only expressed matrix metalloproteinase (MMP) family members but also uniquely upregulated key ECM crosslinking molecules, including TGFBI, LOX, LOXL2/3, and PLOD3. These findings suggest that Clusters 1 and 5 have distinct functional roles in ECM remodeling. Additionally, Clusters 5 exhibited high LOXL2 expression (Fig. S10D). Combined with our RNA-seq results, the GO analysis results revealed that the main enriched molecular functions in subgroup 5 were associated with ECM-related gene sets (Fig. S10E), indicating consistency between MMA.HPSCs and C5.PSCs in terms of effects on ECM remodeling. GSEA confirmed the similarity in the gene expression phenotypes between C5.PSCs and MMA.HPSCs (Fig. S10F), suggesting the existence of a PSC subgroup exhibiting high LOXL2 expression with gene expression phenotypes and molecular functions similar to those of PSCs with MMA-induced LOXL2 expression in the normal pancreas.

**Fig. 5:**
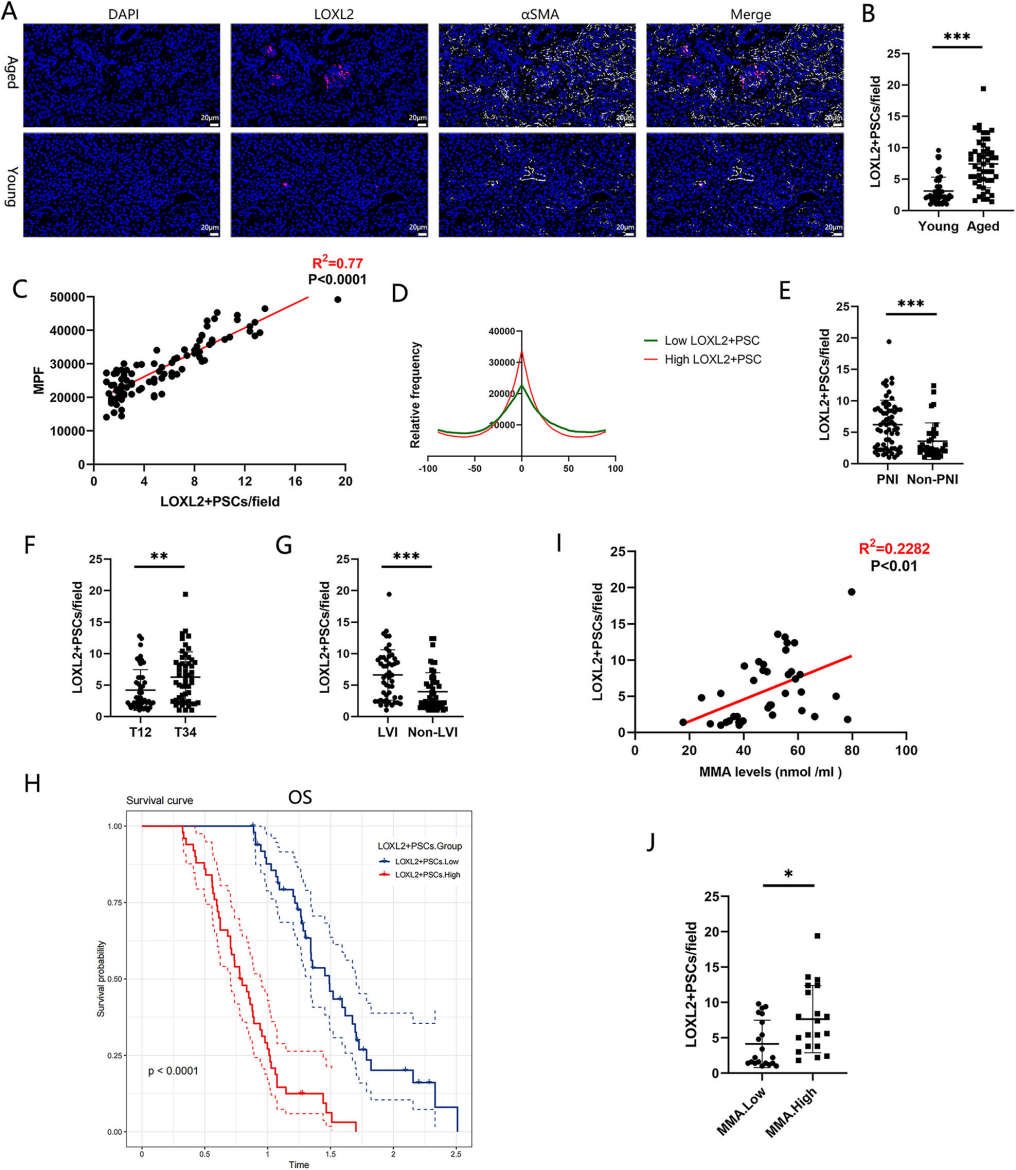
LOXL2^+^ PSCs increase linear alignment of the pancreatic ECM and accelerate PDAC progression. **A** Multiplex IF staining of normal pancreatic tissues from aged and young individuals. **B** Counting of cells in (**A**). **C** Correlation analysis between linear ECM alignment and LOXL2^+^ PSC infiltration in normal human pancreatic tissues. **D** Comparison of linear ECM alignment between the high LOXL2^+^ PSC infiltration group and the low LOXL2^+^ PSC infiltration group. **E** Comparison of LOXL2^+^ PSC infiltration between the PNI-positive and PNI-negative groups. **F** Comparison of LOXL2^+^ PSC infiltration between the T1/T2 and T3/T4 groups. **G** Comparison of LOXL2^+^ PSC infiltration between the LVI-positive and LVI-negative groups. **H** OS of the high LOXL2^+^ PSC infiltration group and the low LOXL2^+^ PSC infiltration group. **I** Correlation analysis between serum MMA levels in PDAC patients and LOXL2^+^ PSC infiltration in normal pancreatic tissues. **J** Comparison of LOXL2^+^ PSC infiltration between the MMA.High and MMA.Low groups. (*p < 0.05, **p < 0.01, ***p < 0.001).

To determine whether LOXL2^+^ PSCs influence linear ECM alignment, we performed multiplex IF staining on paraffin-embedded samples of normal pancreatic tissue to assess the infiltration of LOXL2^+^ PSCs. Compared with the young group, the aged group exhibited significantly greater LOXL2^+^ PSC infiltration (Fig. 5A, B), with linear ECM alignment in pancreatic tissue positively correlated with the number of infiltrating LOXL2^+^ PSCs (Fig. 5C). In the median method, patients were divided into high LOXL2^+^ PSC infiltration and low LOXL2^+^ PSC infiltration groups, with greater linear ECM alignment observed in the high LOXL2^+^ PSC infiltration group (Fig. 5D). These results indicated that LOXL2^+^ PSCs is associated with the increase in linear ECM alignment. The pathological information of the tumor tissues corresponding to 100 adjacent normal pancreatic tissues was analyzed. The number of infiltrating LOXL2^+^ PSCs was greater in the PNI-positive group (Fig. 5E), T3/T4 group (Fig. 5F), and LVI-positive group (Fig. 5G). Moreover, the high LOXL2^+^ PSC infiltration group had shorter OS than the low LOXL2^+^ PSC infiltration group (Fig. 5H). These findings were consistent with the pathological characteristics of the aged group and the high linear ECM alignment group. Serum MMA levels were positively correlated with LOXL2^+^ PSC infiltration in normal pancreatic tissues (Fig. 5I), with the MMA.High group showed significantly higher LOXL2^+^ PSC infiltration compared to the MMA.Low group (Fig. 5J). Therefore, these results indicate that MMA increases LOXL2^+^ PSC infiltration in the aged pancreas, which increases linear ECM alignment and promotes tumor progression.

Building on these findings, we further investigated whether the observed effects of LOXL2^+^ PSCs infiltration and linear ECM alignment have long-term clinical consequences. In our previous study, the PDAC patient cohort from Zhongda Hospital underwent only short-term follow-up (≤3 years). To assess the long-term impact, we conducted long-term follow-up (≥5 years) and collected data on OS and DFS. Our analysis revealed that patients in the aged group (Fig. S11A, B), MPF.High group (Fig. S11C, D), MMA.High group (Fig. S11E, F), and LOXL2^+^ PSCs.High group (Fig. S11G, H) all exhibited shorter OS and DFS. These findings suggest that the effects of MMA—increased LOXL2^+^ PSC infiltration and enhanced linear ECM alignment—not only contribute to short-term tumor progression but also correlate with long-term poor prognosis in PDAC patients.

### MMA upregulates LOXL2 transcription via KLF10/SP1 transcriptional complex in PSCs

**Fig. 6:**
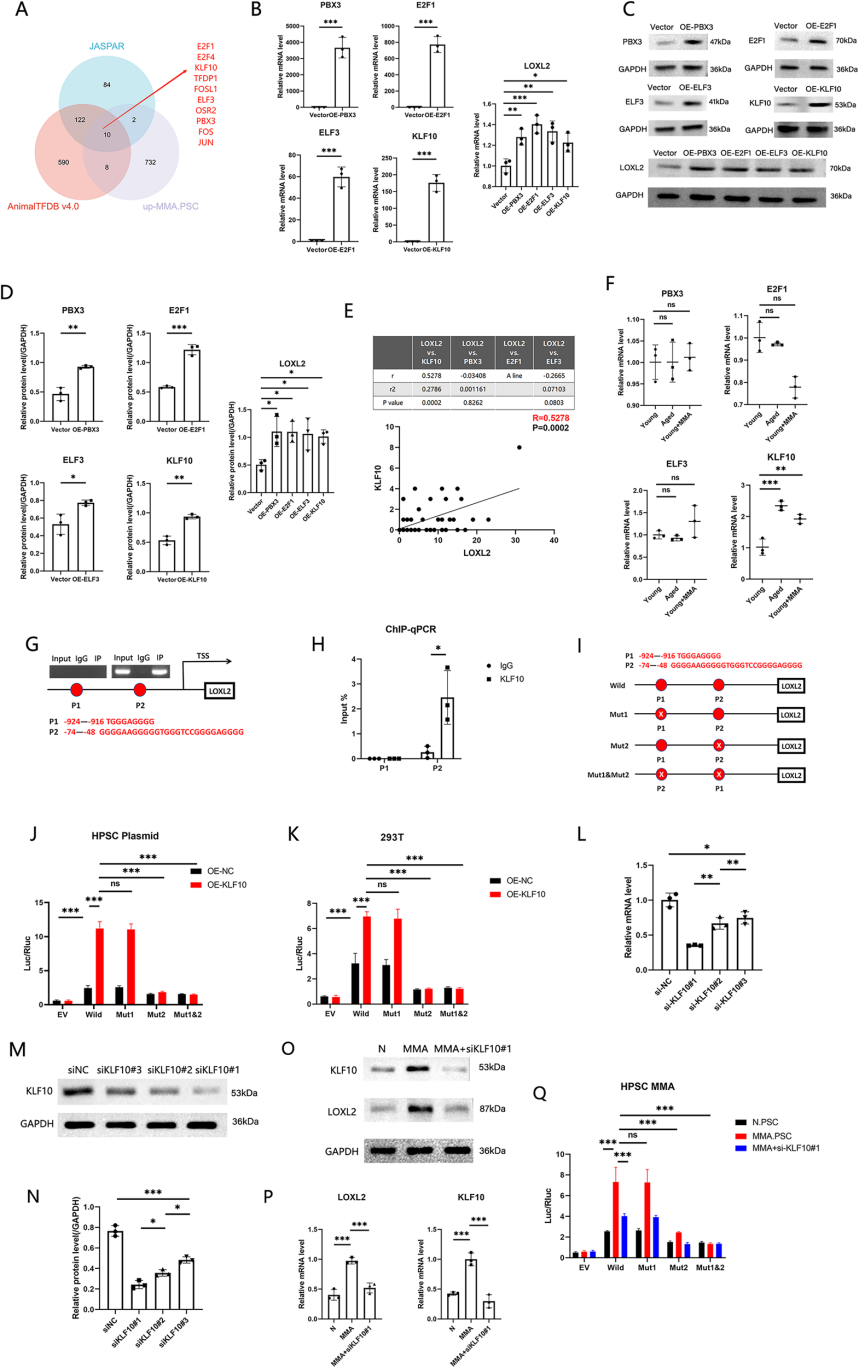
MMA upregulates KLF10 to promote LOXL2 expression. **A** Prediction of potential TFs for LOXL2 from AnimalTFDB 4.0 and the JASPAR database. These potential TFs were intersected with upregulated genes in MMA.HPSCs to identify overlapping genes. **B** qPCR analysis of TF and LOXL2 expression in HPSCs after transfection with the pcDNA-PBX3, pcDNA-E2F1, pcDNA-ELF3, or pcDNA-KLF10 plasmid. (n = 3 biological replicates). **C** Representative WB results and (**D**) quantification of TF and LOXL2 expression in HPSCs after transfection with the pcDNA-PBX3, pcDNA-E2F1, pcDNA-ELF3, or pcDNA-KLF10 plasmids. (n = 3 biological replicates). **E** Correlation analysis of PBX3, KLF10, E2F1, and ELF3 expression in C5.PSCs. **F** qPCR analysis of PBX3, E2F1, KLF10, and ELF3 expression in Young.PSCs, Old.PSCs and Young.PSCs+MMA. (n = 3 biological replicates). **G** Pattern of KLF10 binding sites in the LOXL2 promoter region. **H** ChIP‒qPCR analysis of KLF10 binding sites in the LOXL2 promoter region. (n = 3 biological replicates). **I** Schematic diagram of the plasmid containing the full-length LOXL2 promoter with the wild-type and point mutant sequences. **J** A dual-luciferase reporter assay was performed to evaluate the activation of the LOXL2 promoter in the pcDNA-KLF10.HPSC and control groups. (n = 3 biological replicates). **K** A dual-luciferase reporter assay was used to evaluate the activation of the LOXL2 promoter in the pcDNA-KLF10.293T and control groups. (n = 3 biological replicates). **L** qPCR analysis of the efficiency of siKLF10 knockdown. (n = 3 biological replicates). Representative WB results (**M**) and quantification of KLF10 expression (**N**) after siKLF10 transfection. (n = 3 biological replicates). Representative WB results (**O**) and quantification of KLF10 and LOXL2 expression (**P**) in the MMA.HPSC, MMA.HPSC+siKLF10#1, and control groups. (n = 3 biological replicates). **Q** A dual-luciferase reporter assay was used to evaluate the activation of the LOXL2 promoter in the MMA.HPSC, MMA.HPSC+siKLF10#1, and control groups. (n = 3 biological replicates). (*p < 0.05, **p < 0.01, ***p < 0.001).

To investigate the mechanism underlying the MMA-induced upregulation of LOXL2 in PSCs, we screened for potential transcription factors for LOXL2 and identified 10 potential transcription factors for LOXL2 that were upregulated in PSCs after MMA treatment (Fig. 6A). Among the 10 transcription factors with the highest scores predicted by AnimalTFDB 4.0 and the JASPAR database on the basis of site score and site quantity, four potential transcription factors regulating LOXL2 were ultimately identified: pre-B-cell leukemia transcription factor 3 (PBX3), E2F transcription factor 1 (E2F1), E74-like factor 3 (ELF3), and Kruppel-like factor 10 (KLF10) (Tables S10, S11). After the construction of overexpression plasmids and the transfection of these plasmids into HPSCs, qPCR and WB analyses revealed that PBX3, E2F1, ELF3, and KLF10 promoted LOXL2 expression (Fig. 6B–D). Correlation analysis using C5.PSCs from the GSE84133 scRNA-seq dataset showed that the expression of only KLF10 was positively correlated with LOXL2 expression in this cell subgroup (Fig. 6E). In addition, compared with PSCs from young individuals, PSCs from aged individuals presented upregulation of KLF10 expression, with no difference in PBX3, E2F1, or ELF3 expression. Additionally, after MMA treatment, KLF10 expression was elevated in Young.PSCs, while E2F1 expression was decreased, and PBX3 and ELF3 expression levels remained unchanged (Fig. 6F). Therefore, we selected KLF10 as the upstream transcription factor for further study.

KLF10 has two potential binding sites in the LOXL2 promoter region (Fig. 6G). CHIP–qPCR (Fig. 6H) revealed that KLF10 bound to the P2 site in the LOXL2 promoter region but not to the P1 site. Dual-luciferase reporter assays were performed with plasmids containing the wild-type LOXL2 upstream promoter region (–2000 bp to +100 bp) and point mutants of this region (Fig. 6I), and dual-luciferase reporter assays conducted with HPSCs revealed that KLF10 bound to the P2 site in the LOXL2 promoter region but not to the P1 site (Fig. 6J). Dual-luciferase reporter assays conducted with the 293T cell line further confirmed these findings (Fig. 6K). These results indicate that KLF10 interacts with the P2 site in the LOXL2 promoter region, promoting LOXL2 expression.

To further confirm that the increase in LOXL2 expression in MMA-induced PSCs is due to the upregulation of KLF10 expression and its binding to the P2 site in the LOXL2 promoter region, we constructed siRNA to knock down KLF10 expression (Fig. 6L–N). WB analysis confirmed that both KLF10 and LOXL2 expression were significantly increased following MMA induction, and that KLF10 knockdown led to a marked decrease in LOXL2 expression (Fig. 6O–P). Dual-luciferase reporter assays conducted with MMA-treated HPSCs showed that MMA induction enhanced the activation of the LOXL2 promoter, an effect that was abolished upon deletion of the P2 site (Fig. 6Q). Additionally, knockdown of KLF10 in MMA-treated HPSCs significantly reduced the activation of the LOXL2 promoter, with a more pronounced reduction observed after mutation of the P2 site (Fig. 6Q). These results indicate that the upregulation of LOXL2 expression in MMA-induced PSCs is mediated through the upregulation of KLF10 expression and its binding to the P2 site in the LOXL2 promoter region.

**Fig. 7:**
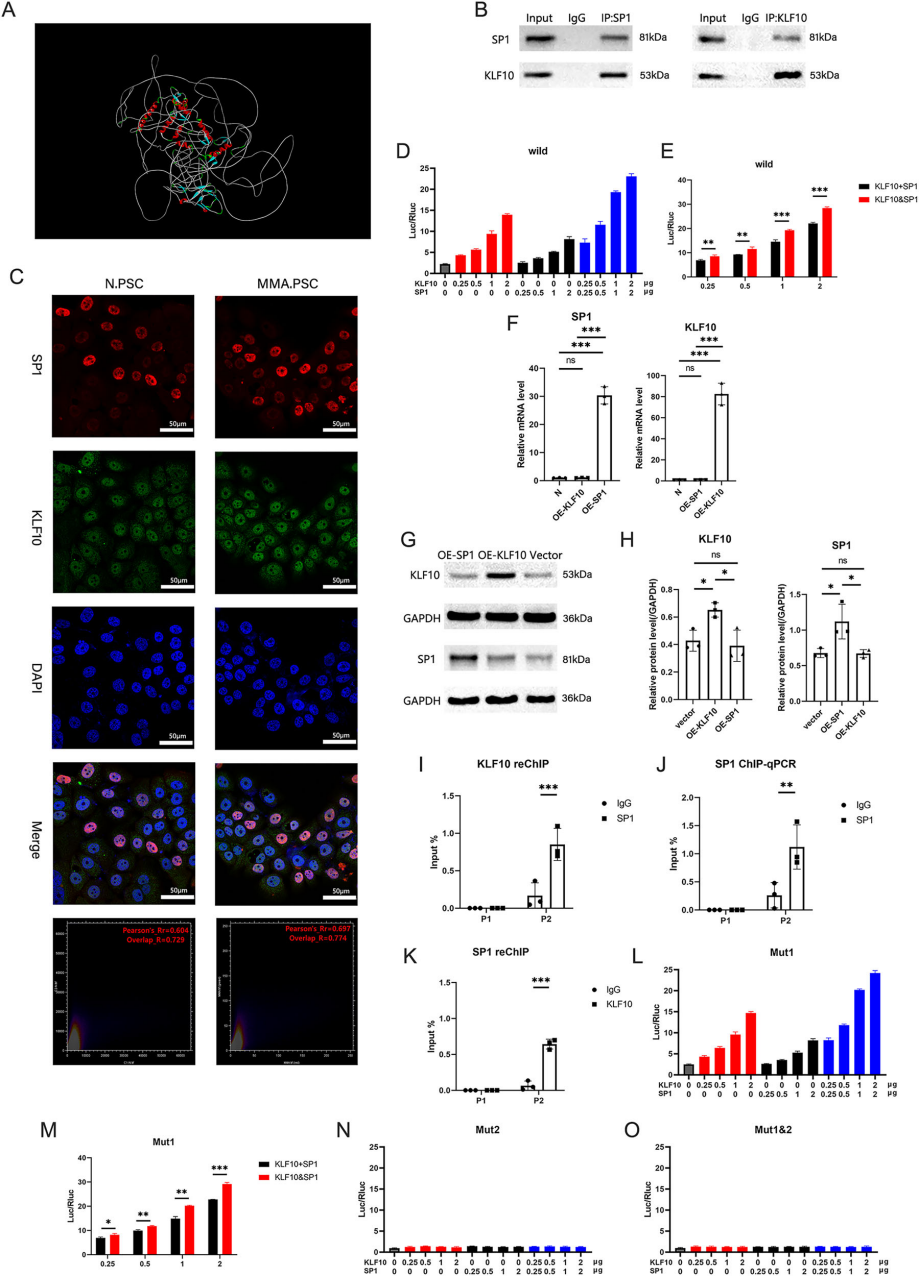
The KLF10/SP1 complex in PSCs promotes LOXL2 expression. **A** Prediction of the binding between KLF10 and SP1 by molecular docking analysis. **B** Co-immunoprecipitation (co-IP) results showing the interaction between KLF10 and SP1. **C** Multiplex IF staining results demonstrating the colocalization of KLF10 and SP1 in MMA-induced HPSCs and uninduced HPSCs. SP1 (red), KLF10 (green). **D** A dual-luciferase reporter assay was performed with the wild-type LOXL2 promoter plasmid to evaluate LOXL2 promoter activation in pcDNA-KLF10.HPSCs, pcDNA-SP1.HPSCs, and pcDNA-KLF10/SP1.HPSCs. (n = 3 biological replicates). **E** Comparison of luciferase activity in pcDNA-KLF10-, pcDNA-SP1-, and pcDNA-KLF10/SP1-transfected cells. (n = 3 biological replicates). **F** qPCR analysis of SP1 and KLF10 expression in HPSCs after transfection with the pcDNA-SP1 or pcDNA-KLF10 plasmid. (n = 3 biological replicates). Representative WB results (**G**) and quantification (**H**) of SP1 and KLF10 expression in HPSCs after transfection with the pcDNA-SP1 or pcDNA-KLF10 plasmid. (n = 3 biological replicates). **I** KLF10 reChIP‒qPCR analysis of KLF10/SP1 complex binding at the LOXL2 promoter region. (n = 3 biological replicates). **J** SP1-ChIP‒qPCR analysis of SP1 binding sites in the LOXL2 promoter region. (n = 3 biological replicates). **K** SP1-reChIP‒qPCR analysis of KLF10/SP1 complex binding at the LOXL2 promoter region. (n = 3 biological replicates). **L** A dual-luciferase reporter assay was performed with the Mut1.LOXL2 promoter plasmid to evaluate LOXL2 promoter activation in pcDNA-KLF10.HPSCs, pcDNA-SP1.HPSCs, and pcDNA-KLF10/SP1.HPSCs. (n = 3 biological replicates). **M** Comparison of luciferase activity in pcDNA-KLF10-, pcDNA-SP1-, and pcDNA-KLF10/SP1-transfected cells. (n = 3 biological replicates). **N** A dual-luciferase reporter assay was performed using the Mut2.LOXL2 promoter plasmid to evaluate LOXL2 promoter activation in pcDNA-KLF10. HPSCs, pcDNA-SP1.HPSCs, and pcDNA-KLF10/SP1.HPSCs. (n = 3 biological replicates). **O** A dual-luciferase reporter assay was performed with the Mut1&2.LOXL2 promoter plasmid to evaluate LOXL2 promoter activation in pcDNA-KLF10.HPSCs, pcDNA-SP1.HPSCs, and pcDNA-KLF10/SP1.HPSCs. (n = 3 biological replicates). (*p < 0.05, **p < 0.01, ***p < 0.001).

The transcription factor SP1 can form a complex with KLF4 [18] or KLF6 [19], and these complexes can exert synergistic effects, resulting in more effective promotion of downstream gene transcription than either transcription factor alone. Molecular docking analysis demonstrated that KLF10 and SP1 can form a complex (Fig. 7A). The presence of the KLF10/SP1 complex in PSCs was subsequently confirmed by Co-IP (Fig. 7B). IF staining colocalization analysis revealed that MMA increased the nuclear colocalization of KLF10 and SP1 in HPSCs (Fig. 7C), indicating that MMA promoted the formation of the KLF10/SP1 complex in the nucleus. Dual-luciferase reporter assays revealed that the luciferase activity resulting from the cotransfection of KLF10 and SP1 was greater than the sum resulting from the transfections of each individually (Fig. 7D, E), suggesting stronger activation of the LOXL2 promoter by the combined effect of KLF10 and SP1 than by each transcription factor alone. Additionally, the overexpression of either SP1 or KLF10 alone in HPSCs did not alter the expression of the other gene (Fig. 7F–H), indicating that the stronger activation of the LOXL2 promoter by cotransfection is due to the synergistic action of the KLF10/SP1 complex. KLF10-reChIP–qPCR analysis revealed that KLF10 binds to the P2 site in the LOXL2 promoter region as part of a complex with SP1 (Fig. 7I). Similarly, SP1 pull-down followed by ChIP‒qPCR and reCHIP‒qPCR revealed that SP1 binds to the P2 site in the LOXL2 promoter as part of a complex with KLF10 and that SP1 does not bind to the P1 site (Fig. 7J, K). Analysis of synergism via a dual-luciferase reporter assay also revealed that the KLF10/SP1 complex synergistically activated the LOXL2 promoter when the P2 site was intact but not when it was mutated, whereas the presence of the P1 site had no effect on the synergistic action of the KLF10/SP1 complex (Fig. 7L–O). Therefore, these results indicate that MMA induction promotes the formation of the KLF10/SP1 complex, which binds to the P2 site in the LOXL2 promoter region in PSCs, thereby increasing LOXL2 expression.

## Discussion

The ECM exhibits significant heterogeneity, encompassing both biochemical and physical properties. ECM heterogeneity manifests spatially across organs and temporally within the same organ over time [3, 20]. Studies have reported that focal fibrosis and atrophy of pancreatic lobules are commonly observed in the pancreas of aged individuals [21]; however, whether linear ECM alignment changes with aging remains unexplored. Our study reveals that linear ECM alignment increases with aging in normal pancreatic tissue. ECM can regulate tumor cell functions through various bioactive proteins, such as cytokines, by paracrine signaling [22, 23]. Additionally, ECM influences tumor cell adhesion, migration, proliferation, and survival through interactions with cell surface receptors like integrins [24]. The physical properties of the ECM, such as stiffness, have also been shown to have a profound impact on tumor cell behavior. Stiffer ECM is often associated with more aggressive tumor phenotypes, as mechanical signals perceived by cells alter the cytoskeleton and signaling pathways [25]. Our study demonstrates that another physical property of ECM, namely linear ECM alignment, is also related to tumor progression. Through both in vitro and in vivo experiments, combined with clinical data, we show that increasing linear ECM alignment can promote tumor progression. Future experiments using genetically engineered mice with LOXL2-specific knockout in PSCs, or spontaneous pancreatic cancer mouse models, may provide more direct evidence of how increasing linear ECM alignment contributes to tumor progression. These experiments could help identify novel therapeutic strategies targeting linear ECM alignment in PDAC. We also find that increased linear ECM alignment in both the adjacent normal pancreas and tumor tissues, elevated serum MMA levels, and increased LOXL2^+^ PSC accumulation in the adjacent normal pancreas are all closely associated with the establishment of an immunosuppressive microenvironment (Fig. S12A–E, Fig. S13A–D). This is consistent with previous findings that both MMA [26] and the enhanced linear ECM alignment [8] contribute to immunosuppressive effects.

PSCs are a specialized type of fibroblast that play crucial roles in maintaining the ECM, immune function, and the synthesis of factors related to digestion [9, 10]. PSCs are a specialized type of fibroblast that play crucial roles in maintaining the ECM, immune function, and the synthesis of factors related to digestion. PSCs exhibit plasticity, and their phenotype can be altered by various factors such as pro-inflammatory cytokines, growth factors, fatty acid esters, and endotoxins, which in turn affect their biological functions. For example, TGF-β activates stellate cells, transforming them into activated stellate cells that subsequently remodel the ECM by synthesizing abundant ECM components such as collagen, fibronectin, laminin, and hyaluronic acid, thereby influencing tumor progression [9, 27]. In this study, we identified a novel PSC phenotype—LOXL2^+^ PSCs induced by MMA—which increases linear ECM alignment and accelerates PDAC progression. This discovery not only deepens our understanding of how age-related changes in the ECM influence tumor progression but also suggests that LOXL2^+^ PSCs could serve as a potential therapeutic target in PDAC. A limitation of our study is that we only used shRNA to downregulate LOXL2 expression or applied enzymatic inhibitors of LOXL2 in PSCs, without exploring pharmacological agents that directly target MMA. Future studies should focus on developing or screening inhibitors to evaluate whether MMA possesses therapeutic potential. Additionally, MMA efficiently enters cells in vivo due to lipid encapsulation, enabling low concentrations (1–100 μM) to promote epithelial-to-mesenchymal transition (EMT) in tumors. In contrast, in vitro experiments require higher concentrations (5–10 mM) due to the lack of lipid-mediated uptake [17]. Therefore, in our experiments, we used 5 mM MMA, which is non-toxic to the cells but still sufficient to induce the desired effects. Moving forward, MMA, as a new metabolic target, could not only be targeted directly but also serve as a new therapeutic target by interfering with its entry into cells. This could provide a dual strategy for disrupting MMA’s role in PDAC progression.

Collectively, our findings not only explained the poorer prognosis observed in aged pancreatic cancer patients but also revealed that MMA can promote LOXL2 expression in PSCs, increasing linear ECM alignment, and accelerate the progression of PDAC (Graphical Abstract 1).

## Supplementary information

Supplementary Tables S1–S11

Supplementary Figures S1–S13

Original Western blots

## Data Availability

The data that support the findings of this study are available from the corresponding author upon reasonable request.
