# Supplementary material for: MMA-induced LOXL2+ PSCs promote linear ECM alignment in the aging pancreas leading to pancreatic cancer progression
Source: Cell Death Dis. 2025 May 27;16(1):419. doi: 10.1038/s41419-025-07751-5 (PMC12116754; doi:10.1038/s41419-025-07751-5)
Supplement: Supplementary file 1 — Supplementary Tables S1–S11 [file 41419_2025_7751_MOESM1_ESM.docx]

**Table S1.The collection criteria of SEER database.**

| {Age at Diagnosis.Age recode with <1 year olds} = '20-24 years','25-29 years','30-34 years','35-39 years','40-44 years','45-49 years','50-54 years','55-59 years','60-64 years','65-69 years','70-74 years','75-79 years','80-84 years','85+ years' |
| --- |
| {Race, Sex, Year Dx.Sex} = ' Male',' Female' |
| {Race, Sex, Year Dx.Sex} = ' Male',' Female' |
| {Race, Sex, Year Dx.Year of diagnosis} = '2000—2019','2000','2001','2002','2003','2004','2005','2006','2007','2008','2009','2010','2011','2012','2013','2014','2015','2016','2017','2018','2019' |
| {Race, Sex, Year Dx.Race recode (W, B, AI, API)} = 'White','Black','American Indian/Alaska Native','Asian or Pacific Islander' |
| {Site and Morphology.Primary Site - labeled} = 'C25.0-Head of pancreas','C25.1-Body of pancreas','C25.2-Tail of pancreas' |
| {Site and Morphology.Behavior code ICD-O-3} = 'Malignant' |
| {Site and Morphology.Histology recode - broad groupings} = '8010-8049: epithelial neoplasms, NOS','8140-8389: adenomas and adenocarcinomas','8500-8549: ductal and lobular neoplasms' |
| {Site and Morphology.Grade (thru 2017)} = 'Well differentiated; Grade I','Moderately differentiated; Grade II','Poorly differentiated; Grade III','Undifferentiated; anaplastic; Grade IV' |
| {Stage - 7th edition.Derived AJCC T, 7th ed (2010-2015)} = 'T0','Ta','Tis','Tispu','Tispd','T1','T1mic','T1a','T1a(s)','T1a(m)','T1a1','T1a2','T1b','T1b(s)','T1b(m)','T1b1','T1b2','T1c','T1d','T1NOS(s)','T1NOS(m)','T1NOS','T2','T2(s)','T2(m)','T2a','T2a1','T2a2','T2aNOS','T2b','T2c','T2d','T2NOS','T3','T3(s)','T3(m)','T3a','T3b','T3c','T3d','T3NOS','T4','T4a','T4a(s)','T4a(m)','T4b','T4b(s)','T4b(m)','T4c','T4d','T4e','T4NOS(s)','T4NOS(m)','T4NOS','T1aNOS','T1bNOS |
| {Stage - 7th edition.Derived AJCC N, 7th ed (2010-2015)} = 'N0','N0(i)','N0(i+)','N0(mol)','N0(mol+)','N1','N1a','N1b','N1c','N1mi','N1NOS','N2','N2a','N2b','N2c','N2NOS','N3','N3a','N3b','N3c','N3NOS','N4' |
| {Stage - 7th edition.Derived AJCC M, 7th ed (2010-2015)} ='M0','M0(i+)','M1','M1a','M1b','M1c','M1d','M1NOS' |
| {Stage - 7th edition.Derived AJCC Stage Group, 7th ed (2010-2015)} = '0','0a','0is','I','INOS','IA','IANOS','IA1','IA2','IB','IBNOS','IB1','IB2','IC','IS','IEA','IEB','IE','ISA','ISB','II','IINOS','IIA','IIANOS','IIA1','IIA2','IIB','IIC','IIEA','IIEB','IIE','IISA','IISB','IIS','IIESA','IIESB','IIES','III','IIINOS','IIIA','IIIB','IIIC','IIIC1','IIIC2','IIIEA','IIIEB','IIIE','IIISA','IIISB','IIIS','IIIESA','IIIESB','IIIES','IV','IVNOS','IVA','IVA1','IVA2','IVB','IVC' |
| {Cause of Death (COD) and Follow-up.Survival months} != 'Unknown' |
| {Cause of Death (COD) and Follow-up.Vital status recode (study cutoff used)} = 'Alive','Dead' |

**Table S2. Clinicopathologic characteristics of PDAC patients in SEER database.**

| Parameters | Cases (n) | (%) |
| --- | --- | --- |
| Age |  |  |
| Young | 5964 | 41.88 |
| Old | 8278 | 58.12 |
| Sex |  |  |
| Male | 7441 | 52.25 |
| Female | 6801 | 47.75 |
| Race |  |  |
| white | 11505 | 80.78 |
| black | 1494 | 10.49 |
| others | 1243 | 8.73 |
| Location |  |  |
| head | 9632 | 67.63 |
| body | 1969 | 13.83 |
| tail | 2641 | 18.54 |
| Grade |  |  |
| G1 | 3030 | 21.28 |
| G2 | 5857 | 41.12 |
| G3 | 5043 | 35.41 |
| G4 | 312 | 2.19 |
| Stage_T |  |  |
| T1 | 1319 | 9.26 |
| T2 | 2675 | 18.78 |
| T3 | 8387 | 58.89 |
| T4 | 1861 | 13.07 |
| Stage_N |  |  |
| NO | 7098 | 49.84 |
| N1 | 7144 | 50.16 |
| Stage_M |  |  |
| MO | 11019 | 77.37 |
| M1 | 3223 | 22.63 |

**Table S3. Clinicopathologic characteristics of PDAC patients in Zhongda Hospital.**

| Parameters | Cases (n) | (%) |
| --- | --- | --- |
| Age |  |  |
| Young | 50 | 0.35 |
| Old | 50 | 0.35 |
| Grade |  |  |
| G1 | 21 | 0.15 |
| G2 | 56 | 0.39 |
| G3 | 23 | 0.16 |
| Stage_T |  |  |
| T1 | 17 | 0.12 |
| T2 | 31 | 0.22 |
| T3 | 35 | 0.25 |
| T4 | 17 | 0.12 |
| Stage_N |  |  |
| NO | 31 | 0.22 |
| N1 | 69 | 0.48 |
| LVI |  |  |
| LVI | 50 | 0.35 |
| Non-LVI | 50 | 0.35 |
| PNI |  |  |
| PNI | 65 | 0.46 |
| Non-PNI | 35 | 0.25 |

**Table S4. Antibodies.**

| **Primary antibodies** | | | | |
| --- | --- | --- | --- | --- |
| **Name** | **Manufacturer** | **Catalogue number** | **Type** | **Usage** |
| Collagen I | Abcam | ab138492 | monoclonal | IF1:500 |
| α-SMA | Abcam | ab124964 | monoclonal | IF1:500 |
| LOXL2 | Abcam | ab96233 | polyclonal | IF1:500 |
| KLF10 | Abcam | ab184182 | monoclonal | WB1:1000 |
| SP1 | Abcam | ab231778 | monoclonal | WB1:1000 |
| E2F1 | Abcam | ab314311 | monoclonal | WB1:1000 |
| ELF3(ESE1) | Abcam | ab133621 | monoclonal | WB1:1000 |
| PBX3 | Abcam | ab109173 | monoclonal | WB1:1000 |
| LOXL2 | Abcam | ab260046 | monoclonal | WB1:1000 |
| α-SMA | Abcam | ab124964 | monoclonal | WB1:20000 |
| Collagen I | Proteintech | 67288-1-Ig | monoclonal | WB1:10000 |
| GAPDH | Abcam | ab181602 | monoclonal | WB1:10000 |
| KLF10 | Thermo Fisher | PA5-28873 | polyclonal | CHIP:10μl/IP |
| SP1 | Abcam | ab231778 | monoclonal | CHIP:10μl/IP |
| KLF10 | Thermo Fisher | PA5-28873 | polyclonal | CO-IP:7μl/IP |
| SP1 | Abcam | ab231778 | monoclonal | CO-IP:8μl/IP |
| CD163 | Abcam | ab182422 | monoclonal | IF1:100 |
| CD4 | Abcam | ab133616 | monoclonal | IF1:100 |
| CD8 alpha | Abcam | ab237709 | monoclonal | IF1:100 |
| **Secondary antibodies** | | | | |
| **Manufacturer** | **Catalogue number** | **Usage** |  |  |
| Servicebio | GB23303 | WB1:10000 |  |  |
| Servicebio | GB21303 | IF 1:300 |  |  |
| Servicebio | GB25303 | IF 1:300 |  |  |

**Table S5. Primers.**

| **RT-qPCR** | |
| --- | --- |
| Name | Primer sequences |
| human-GAPDH-F | TGACATCAAGAAGGTGGTGAAGCAG |
| human-GAPDH-R | GTGTCGCTGTTGAAGTCAGAGGAG |
| human-LOXL2-F | TGAAGAATGTCACCTGCGAGAATGG |
| human-LOXL2-R | TTGCTCTGGCTTGTACGCTTTCC |
| human-KLF10-F | GCCAGCATCCTCAACTATCAGAAC |
| human-KLF10-R | GACACAGCGGCACATGGTATG |
| human-ELF3-F | TGGAGAAGAACAAGTACGACGCAAG |
| hunman-ELF3-R | AGACCAGACGCAGCTCCTCAAG |
| human-E2F1-F | TGCCAAGAAGTCCAAGAACCACATC |
| human-E2F1-R | TGTCGGAGGTCCTGGGTCAAC |
| human-PBX3-F | CAGCATCACAGTGTCACAGGTATCC |
| human-PBX3-R | TGGGCGAATTGGTCTGGTTGTTC |
| human-SP1-F | CAAGCCCAAACAATCACCTTAGCC |
| human-SP1-R | TGGAGGAGAGTTGAGCAGCATTC |
| human-COL1A1-F | TGGCAAAGAAGGCGGCAAAGG |
| human-COL1A1-R | AGGAGCACCAGCAGGACCATC |
| human-COL1A2-F | CCGTGGCAGTGATGGAAGTGTG |
| human-COL1A2-R | GCAGGACCAGCGTTACCAACAG |
| human-ACTA2-F | CTTCGTTACTACTGCTGAGCGTGAG |
| human-ACTA2-R | CCCATCAGGCAACTCGTAACTCTTC |
| **CHIP-qPCR** | |
| Name | Primer sequences |
| human-LOXL2.P1-Sense | ATCTCCCGAGGAAAGGC |
| human-LOXL2.P1-Anti-sense | AAGCAAGCACCAAGCGTA |
| human-LOXL2.P2-Sense | GGAATGTCCAGGTGAAAGTCT |
| human-LOXL2.P2-Anti-sense | AGTCCCAAGGAGGCAGC |

**Table S6.siKLF10 sequence.**

|  | **sense（5'-3'）** | **antisense（5'-3'）** |
| --- | --- | --- |
| siKLF10#1 | GGCAGAUGUUGAUGAGAAATT | UUUCUCAUCAACAUCUGCCTT |
| siKLF10#2 | CCAGCAUCCUCAACUAUCATT | UGAUAGUUGAGGAUGCUGGTT |
| siKLF10#3 | CAGCCAAGAAGCUACCAAATT | UUUGGUAGCUUCUUGGCUGTT |
| siNC | UUCUCCGAACGUGUCACGUTT | ACGUGACACGUUCGGAGAATT |

**Table S7**

| **Only GOBP_COLLAGEN_FIBRIL_ORGANIZATION** | **Only Matrisome** | **Only MMA.PSC up** | **GOBP_COLLAGEN_FIBRIL_ORGANIZATION AND Matrisome NOT MMA.PSC up** | **GOBP_COLLAGEN_FIBRIL_ORGANIZATION AND MMA.PSC up NOT Matrisome** | **Matrisome AND MMA.PSC up NOT GOBP_COLLAGEN_FIBRIL_ORGANIZATION** | **GOBP_COLLAGEN_FIBRIL_ORGANIZATION AND Matrisome AND MMA.PSC up** |
| --- | --- | --- | --- | --- | --- | --- |
| CRTAP | PXDNL | AATK | EMILIN1 | TLL2 | COL6A1 | COL2A1 |
| P3H4 | SSPO | ABCG2 | COL1A1 |  | ZP3 | COL11A2 |
| ADAMTS7 | LAMB4 | ABCG5 | COL1A2 |  | IGFBP5 | LOXL2 |
| ADAMTS14 | VWA3A | ABHD14A | COL3A1 |  | CCN3 | P4HA1 |
| CYP1B1 | OTOL1 | ACKR3 | COL5A1 |  | RELN |  |
| EXT1 | COL6A6 | ACP3 | COL5A2 |  | LAMB3 |  |
| FOXC1 | COL6A5 | ACP5 | COL11A1 |  | LAMC2 |  |
| FOXC2 | MATN2 | ACSL5 | COL12A1 |  | ECM1 |  |
| COLGALT2 | AGRN | ACTR3B | COL13A1 |  | CTHRC1 |  |
| VPS33B | VWA5A | ADAMTS17 | COMP |  | VWCE |  |
| GREM1 | CCN1 | ADAMTSL4 | CHADL |  | COL20A1 |  |
| LOXL1 | NTN3 | ADCY3 | AEBP1 |  | PCOLCE2 |  |
| MIR29B1 | NCAN | ADI1 | DPT |  | ITIH4 |  |
| MMP11 | LAMA5 | ADM | FMOD |  | ANGPTL4 |  |
| NF1 | MATN3 | ADRB1 | OPTC |  |  |  |
| DDR2 | CHAD | AGBL3 | EFEMP2 |  |  |  |
| ATP7A | COCH | AGMAT | ANXA2 |  |  |  |
| RB1 | EDIL3 | AHSA1 | LOX |  |  |  |
| FKBP10 | SRPX2 | AK1 | LUM |  |  |  |
| VIPAS39 | KERA | AKR1C1 | COL5A3 |  |  |  |
| SFRP2 | SLIT1 | AKR1C2 | SERPINF2 |  |  |  |
| SCX | SLIT3 | AKR7A3 | TNXB |  |  |  |
| BMP1 | CILP | ALDOA | COL14A1 |  |  |  |
| TGFB2 | TECTA | ALDOC | PXDN |  |  |  |
| TGFBR1 | CCN5 | ALG3 | LOXL4 |  |  |  |
| TLL1 | ECM2 | ALPG | LOXL3 |  |  |  |
| TNXA | SLIT2 | ALPP | SERPINH1 |  |  |  |
| COLGALT1 | CCN4 | AMIGO3 | PLOD3 |  |  |  |
| ADAMTS12 | CCN6 | ANG |  |  |  |  |
| ADAMTS3 | PAPLN | ANKRD13B |  |  |  |  |
| ADAMTS2 | MATN4 | AOC2 |  |  |  |  |
|  | NTN1 | AOC3 |  |  |  |  |
|  | LGI1 | APLN |  |  |  |  |
|  | COL4A1 | APOE |  |  |  |  |
|  | FGA | AQP1 |  |  |  |  |
|  | FGB | AQP11 |  |  |  |  |
|  | FGG | ARF4 |  |  |  |  |
|  | LRG1 | ARHGAP44 |  |  |  |  |
|  | FN1 | ARID3C |  |  |  |  |
|  | BGLAP | ARL14EPL |  |  |  |  |
|  | VTN | ARL4A |  |  |  |  |
|  | VWF | ARMCX5-GPRASP2 |  |  |  |  |
|  | DCN | ARNTL |  |  |  |  |
|  | LAMB1 | ARRDC4 |  |  |  |  |
|  | THBS1 | ARSG |  |  |  |  |
|  | MGP | ARSL |  |  |  |  |
|  | COL4A2 | ASB9 |  |  |  |  |
|  | IGFBP1 | ASIC3 |  |  |  |  |
|  | SPARC | ATAD3A |  |  |  |  |
|  | SRGN | ATAD3B |  |  |  |  |
|  | SPP1 | ATG9B |  |  |  |  |
|  | HAPLN1 | ATIC |  |  |  |  |
|  | LAMC1 | ATP1B2 |  |  |  |  |
|  | COL6A2 | ATP6V0D2 |  |  |  |  |
|  | COL6A3 | B3GAT3 |  |  |  |  |
|  | VCAN | BAMBI |  |  |  |  |
|  | PRG2 | BARX2 |  |  |  |  |
|  | NID1 | BATF3 |  |  |  |  |
|  | ELN | BBS5 |  |  |  |  |
|  | ACAN | BCL2L10 |  |  |  |  |
|  | IGFBP3 | BCS1L |  |  |  |  |
|  | IGFBP2 | BEX5 |  |  |  |  |
|  | OGN | BICD1 |  |  |  |  |
|  | COL9A1 | BMP6 |  |  |  |  |
|  | BGN | BOD1 |  |  |  |  |
|  | IBSP | BOP1 |  |  |  |  |
|  | MATN1 | BPIFA2 |  |  |  |  |
|  | IGFBP4 | BUD23 |  |  |  |  |
|  | FBLN1 | C12orf76 |  |  |  |  |
|  | ANOS1 | C17orf99 |  |  |  |  |
|  | LAMA2 | C19orf71 |  |  |  |  |
|  | IGFBP6 | C1QBP |  |  |  |  |
|  | TNC | C1orf210 |  |  |  |  |
|  | COL8A2 | C1orf216 |  |  |  |  |
|  | LAMA1 | C20orf204 |  |  |  |  |
|  | COL8A1 | C20orf27 |  |  |  |  |
|  | CCN2 | C2CD2L |  |  |  |  |
|  | COL4A5 | C3orf14 |  |  |  |  |
|  | THBS2 | C3orf33 |  |  |  |  |
|  | THBS4 | C7orf57 |  |  |  |  |
|  | FBN1 | C8orf44-SGK3 |  |  |  |  |
|  | FBN2 | C9orf78 |  |  |  |  |
|  | IGFALS | CA12 |  |  |  |  |
|  | COL15A1 | CA2 |  |  |  |  |
|  | COL18A1 | CA9 |  |  |  |  |
|  | THBS3 | CACYBP |  |  |  |  |
|  | PRELP | CAD |  |  |  |  |
|  | COL4A4 | CALHM6 |  |  |  |  |
|  | MFAP2 | CAMK2N1 |  |  |  |  |
|  | MFAP1 | CAND2 |  |  |  |  |
|  | MFAP3 | CARD17 |  |  |  |  |
|  | MFAP4 | CARD18 |  |  |  |  |
|  | LAMB2 | CARMIL2 |  |  |  |  |
|  | ZP1 | CARS2 |  |  |  |  |
|  | SRPX | CC2D2B |  |  |  |  |
|  | TNFAIP6 | CCDC113 |  |  |  |  |
|  | FBLN2 | CCDC34 |  |  |  |  |
|  | HSPG2 | CCDC78 |  |  |  |  |
|  | COL4A3 | CCDC86 |  |  |  |  |
|  | COL7A1 | CCL26 |  |  |  |  |
|  | COL10A1 | CCNB1IP1 |  |  |  |  |
|  | ZP2 | CCNQ |  |  |  |  |
|  | COL16A1 | CCT5 |  |  |  |  |
|  | BSPH1 | CD163L1 |  |  |  |  |
|  | MFGE8 | CD52 |  |  |  |  |
|  | SPOCK1 | CD68 |  |  |  |  |
|  | FGL1 | CD70 |  |  |  |  |
|  | EFEMP1 | CDA |  |  |  |  |
|  | ZP4 | CDC123 |  |  |  |  |
|  | MMRN1 | CDC26 |  |  |  |  |
|  | DMP1 | CDC45 |  |  |  |  |
|  | MFAP5 | CDCA7 |  |  |  |  |
|  | COL4A6 | CDHR3 |  |  |  |  |
|  | COL9A3 | CDK20 |  |  |  |  |
|  | COL9A2 | CENPS-CORT |  |  |  |  |
|  | NID2 | CERS2 |  |  |  |  |
|  | FGL2 | CFAP20DC |  |  |  |  |
|  | GAS6 | CFAP298 |  |  |  |  |
|  | SPARCL1 | CHCHD3 |  |  |  |  |
|  | LTBP1 | CHORDC1 |  |  |  |  |
|  | LTBP2 | CHST11 |  |  |  |  |
|  | COL19A1 | CHST7 |  |  |  |  |
|  | POSTN | CISD3 |  |  |  |  |
|  | PCOLCE | CLCN2 |  |  |  |  |
|  | TGFBI | CLEC7A |  |  |  |  |
|  | ADIPOQ | CLK1 |  |  |  |  |
|  | IGFBP7 | CLMP |  |  |  |  |
|  | LAMA4 | CLN6 |  |  |  |  |
|  | LAMA3 | CLU |  |  |  |  |
|  | IMPG1 | CMSS1 |  |  |  |  |
|  | COL24A1 | CNBD2 |  |  |  |  |
|  | RSPO4 | CNBP |  |  |  |  |
|  | RSPO1 | CNFN |  |  |  |  |
|  | COL28A1 | CNIH2 |  |  |  |  |
|  | SVEP1 | CNNM1 |  |  |  |  |
|  | FNDC1 | CNTNAP3C |  |  |  |  |
|  | VWA3B | COA6 |  |  |  |  |
|  | FBLN7 | COPRS |  |  |  |  |
|  | VWA2 | COQ10A |  |  |  |  |
|  | INTS6L | COQ8A |  |  |  |  |
|  | EYS | COTL1 |  |  |  |  |
|  | VWA5B1 | COX16 |  |  |  |  |
|  | FNDC7 | CPA4 |  |  |  |  |
|  | CDCP2 | CPLX1 |  |  |  |  |
|  | EGFLAM | CPNE7 |  |  |  |  |
|  | VWA1 | CRADD |  |  |  |  |
|  | PODNL1 | CREG2 |  |  |  |  |
|  | POMZP3 | CRLS1 |  |  |  |  |
|  | CRELD2 | CRPPA |  |  |  |  |
|  | VIT | CRYAB |  |  |  |  |
|  | NPNT | CRYZL2P-SEC16B |  |  |  |  |
|  | RSPO2 | CSF2 |  |  |  |  |
|  | IGSF10 | CSMD3 |  |  |  |  |
|  | GLDN | CSTF2 |  |  |  |  |
|  | THSD4 | CT83 |  |  |  |  |
|  | OTOG | CTPS1 |  |  |  |  |
|  | KCP | CTSV |  |  |  |  |
|  | FBN3 | CTU2 |  |  |  |  |
|  | PODN | CXCL3 |  |  |  |  |
|  | ABI3BP | CXCR4 |  |  |  |  |
|  | HAPLN4 | CYB561D2 |  |  |  |  |
|  | FRAS1 | CYP1A1 |  |  |  |  |
|  | COL23A1 | CYP24A1 |  |  |  |  |
|  | CILP2 | CYP27B1 |  |  |  |  |
|  | SBSPON | CYP39A1 |  |  |  |  |
|  | COL27A1 | CYP4F11 |  |  |  |  |
|  | LGI2 | CYP4F3 |  |  |  |  |
|  | LGI4 | CYP4V2 |  |  |  |  |
|  | LGI3 | DAW1 |  |  |  |  |
|  | VWDE | DCAF13 |  |  |  |  |
|  | LTBP4 | DCK |  |  |  |  |
|  | VWA5B2 | DCLK1 |  |  |  |  |
|  | BMPER | DCTD |  |  |  |  |
|  | HMCN2 | DDIT3 |  |  |  |  |
|  | COL22A1 | DDIT4 |  |  |  |  |
|  | NDNF | DDN |  |  |  |  |
|  | FNDC8 | DEDD2 |  |  |  |  |
|  | ZPLD1 | DHDH |  |  |  |  |
|  | SNED1 | DHODH |  |  |  |  |
|  | NTN5 | DHRS9 |  |  |  |  |
|  | TSPEAR | DHX37 |  |  |  |  |
|  | TSKU | DKC1 |  |  |  |  |
|  | OIT3 | DKK4 |  |  |  |  |
|  | IGFBPL1 | DNAAF4 |  |  |  |  |
|  | SPOCK2 | DNAH17 |  |  |  |  |
|  | TNR | DNAJA1 |  |  |  |  |
|  | NELL1 | DNAJA3 |  |  |  |  |
|  | PRG4 | DNAJA4 |  |  |  |  |
|  | COL26A1 | DNAJB1 |  |  |  |  |
|  | EMID1 | DNAJB4 |  |  |  |  |
|  | ELSPBP1 | DPF1 |  |  |  |  |
|  | NTNG2 | DPF3 |  |  |  |  |
|  | BCAN | DPM2 |  |  |  |  |
|  | CRELD1 | DRAP1 |  |  |  |  |
|  | COL21A1 | DSCC1 |  |  |  |  |
|  | TECTB | DUSP1 |  |  |  |  |
|  | HMCN1 | DUSP2 |  |  |  |  |
|  | HAPLN3 | DYDC2 |  |  |  |  |
|  | INTS14 | E2F1 |  |  |  |  |
|  | AMELX | E2F4 |  |  |  |  |
|  | AMELY | EBNA1BP2 |  |  |  |  |
|  | NELL2 | EEF1A2 |  |  |  |  |
|  | EPYC | EEF1AKMT1 |  |  |  |  |
|  | OMD | EEF1AKMT3 |  |  |  |  |
|  | SPOCK3 | EEF1E1 |  |  |  |  |
|  | SPON2 | EID3 |  |  |  |  |
|  | ASPN | EIF2B3 |  |  |  |  |
|  | COL25A1 | EIF3B |  |  |  |  |
|  | EMILIN2 | EIF3C |  |  |  |  |
|  | RSPO3 | ELF3 |  |  |  |  |
|  | IMPG2 | EMC7 |  |  |  |  |
|  | TINAGL1 | ENO2 |  |  |  |  |
|  | NYX | ENOX1 |  |  |  |  |
|  | HAPLN2 | ENPP2 |  |  |  |  |
|  | CRISPLD2 | ENTR1 |  |  |  |  |
|  | CRISPLD1 | EPDR1 |  |  |  |  |
|  | SMOC2 | EPHX1 |  |  |  |  |
|  | SMOC1 | EPHX4 |  |  |  |  |
|  | MMRN2 | ESYT3 |  |  |  |  |
|  | NTN4 | EWSR1 |  |  |  |  |
|  | SPON1 | EXOSC3 |  |  |  |  |
|  | AMBN | EXOSC5 |  |  |  |  |
|  | ESM1 | FABP5 |  |  |  |  |
|  | MEPE | FAM104B |  |  |  |  |
|  | MXRA5 | FAM174B |  |  |  |  |
|  | LTBP3 | FAM186B |  |  |  |  |
|  | EMILIN3 | FAM216A |  |  |  |  |
|  | CRIM1 | FAM222A |  |  |  |  |
|  | DSPP | FAM228B |  |  |  |  |
|  | FBLN5 | FAM234B |  |  |  |  |
|  | DMBT1 | FAM25A |  |  |  |  |
|  | TINAG | FAM81A |  |  |  |  |
|  | COL17A1 | FAM81B |  |  |  |  |
|  | TNN | FARSB |  |  |  |  |
|  | COLQ | FBLIM1 |  |  |  |  |
|  | NTNG1 | FBXO39 |  |  |  |  |
|  | PRG3 | FCRLB |  |  |  |  |
|  | VWA7 | FGF11 |  |  |  |  |
|  | LAMC3 | FGFR1 |  |  |  |  |
|  | ANXA1 | FGFR4 |  |  |  |  |
|  | ANXA5 | FKBP4 |  |  |  |  |
|  | ANXA6 | FMO3 |  |  |  |  |
|  | ANXA7 | FOLR3 |  |  |  |  |
|  | ANXA9 | FOS |  |  |  |  |
|  | ANXA11 | FOSL1 |  |  |  |  |
|  | CSPG4 | FRMD3 |  |  |  |  |
|  | CLEC14A | FTH1 |  |  |  |  |
|  | COLEC12 | FTL |  |  |  |  |
|  | FREM1 | FUNDC2 |  |  |  |  |
|  | FREM2 | FUS |  |  |  |  |
|  | ITIN1 | FXN |  |  |  |  |
|  | LGALS3 | G0S2 |  |  |  |  |
|  | LGALS4 | GADD45A |  |  |  |  |
|  | MUC2 | GADD45B |  |  |  |  |
|  | PLXDC2 | GADD45G |  |  |  |  |
|  | PLXNB2 | GALNT18 |  |  |  |  |
|  | SEMA3C | GAREM2 |  |  |  |  |
|  | SFTPA1 | GAST |  |  |  |  |
|  | SFTPB | GCNT3 |  |  |  |  |
|  | SFTPD | GCSH |  |  |  |  |
|  | ADAM10 | GDF15 |  |  |  |  |
|  | ADAM19 | GDPD3 |  |  |  |  |
|  | ADAMTS1 | GJB7 |  |  |  |  |
|  | ADAMTSL1 | GLIS3 |  |  |  |  |
|  | ADAMTSL5 | GLS2 |  |  |  |  |
|  | AMBP | GPAT3 |  |  |  |  |
|  | CPN2 | GPD1 |  |  |  |  |
|  | CTSB | GPNMB |  |  |  |  |
|  | ELANE | GPR160 |  |  |  |  |
|  | F13A1 | GPR50 |  |  |  |  |
|  | HTRA1 | GPR89B |  |  |  |  |
|  | ITIH1 | GPX2 |  |  |  |  |
|  | ITIH2 | GTF2A2 |  |  |  |  |
|  | ITIH3 | GYG1 |  |  |  |  |
|  | ITIH5 | GZMB |  |  |  |  |
|  | LOXL | H1-3 |  |  |  |  |
|  | MMP9 | H1-4 |  |  |  |  |
|  | PIG | H2AC13 |  |  |  |  |
|  | PLOD1 | H2AC14 |  |  |  |  |
|  | PZP | H2AC17 |  |  |  |  |
|  | SERPINA1 | H2AC18 |  |  |  |  |
|  | SERPINA3 | H2AC19 |  |  |  |  |
|  | SERPINC1 | H2AC20 |  |  |  |  |
|  | SERPING1 | H2AC21 |  |  |  |  |
|  | TGM2 | H2AJ |  |  |  |  |
|  | TGM3 | H2BC11 |  |  |  |  |
|  | HCFC1 | H2BC14 |  |  |  |  |
|  | IL6 | H2BC18 |  |  |  |  |
|  | INSI5 | H2BC21 |  |  |  |  |
|  | PF4 | H2BC4 |  |  |  |  |
|  | S100A11 | H2BC5 |  |  |  |  |
|  | S100A13 | H2BU1 |  |  |  |  |
|  | EGFL7 | H3C10 |  |  |  |  |
|  | CD109 | H3C13 |  |  |  |  |
|  | F2 | H3C2 |  |  |  |  |
|  | LEPREL2 | H3C4 |  |  |  |  |
|  | LOX1 | H4C9 |  |  |  |  |
|  | PLG | HAUS7 |  |  |  |  |
|  | PLOD2 | HBA1 |  |  |  |  |
|  | SERPINALA | HBA2 |  |  |  |  |
|  | SERPINA3K | HDHD5 |  |  |  |  |
|  | SERPINB1 | HHEX |  |  |  |  |
|  | SERPINE2 | HILPDA |  |  |  |  |
|  | SERPINF1 | HM13 |  |  |  |  |
|  | LGALS1 | HNMT |  |  |  |  |
|  | LMAN1 | HOXA10 |  |  |  |  |
|  | S100A10 | HOXB2 |  |  |  |  |
|  | S100A4 | HOXC9 |  |  |  |  |
|  | S100A6 | HSD11B2 |  |  |  |  |
|  | TGFB1 | HSD17B3 |  |  |  |  |
|  |  | HSD17B6 |  |  |  |  |
|  |  | HSF2BP |  |  |  |  |
|  |  | HSPA1A |  |  |  |  |
|  |  | HSPA1B |  |  |  |  |
|  |  | HSPA2 |  |  |  |  |
|  |  | HSPA6 |  |  |  |  |
|  |  | HSPA8 |  |  |  |  |
|  |  | HSPH1 |  |  |  |  |
|  |  | HTR7 |  |  |  |  |
|  |  | HYPK |  |  |  |  |
|  |  | ID2 |  |  |  |  |
|  |  | ID3 |  |  |  |  |
|  |  | IDH3A |  |  |  |  |
|  |  | IER5 |  |  |  |  |
|  |  | IFT27 |  |  |  |  |
|  |  | IL17RB |  |  |  |  |
|  |  | IL1R2 |  |  |  |  |
|  |  | IL1RL1 |  |  |  |  |
|  |  | IL23A |  |  |  |  |
|  |  | IL27RA |  |  |  |  |
|  |  | IL6R |  |  |  |  |
|  |  | IMP4 |  |  |  |  |
|  |  | INSC |  |  |  |  |
|  |  | INTS10 |  |  |  |  |
|  |  | IPO4 |  |  |  |  |
|  |  | IQCE |  |  |  |  |
|  |  | ISM1 |  |  |  |  |
|  |  | ISOC1 |  |  |  |  |
|  |  | ITGA5 |  |  |  |  |
|  |  | ITGA7 |  |  |  |  |
|  |  | ITGAE |  |  |  |  |
|  |  | ITGB1BP1 |  |  |  |  |
|  |  | ITGB7 |  |  |  |  |
|  |  | ITPR1 |  |  |  |  |
|  |  | IZUMO4 |  |  |  |  |
|  |  | JCAD |  |  |  |  |
|  |  | JHY |  |  |  |  |
|  |  | JUN |  |  |  |  |
|  |  | KARS1 |  |  |  |  |
|  |  | KAT2A |  |  |  |  |
|  |  | KCNC4 |  |  |  |  |
|  |  | KCTD13 |  |  |  |  |
|  |  | KCTD7 |  |  |  |  |
|  |  | KHDC1L |  |  |  |  |
|  |  | KHDRBS3 |  |  |  |  |
|  |  | KIAA1549L |  |  |  |  |
|  |  | KIF17 |  |  |  |  |
|  |  | KIF26B |  |  |  |  |
|  |  | KISS1R |  |  |  |  |
|  |  | KLF10 |  |  |  |  |
|  |  | KLF2 |  |  |  |  |
|  |  | KLHL25 |  |  |  |  |
|  |  | KLHL35 |  |  |  |  |
|  |  | KLK7 |  |  |  |  |
|  |  | KPNA7 |  |  |  |  |
|  |  | KREMEN2 |  |  |  |  |
|  |  | KRT14 |  |  |  |  |
|  |  | KRT16 |  |  |  |  |
|  |  | KRT86 |  |  |  |  |
|  |  | LAMTOR5 |  |  |  |  |
|  |  | LCA5L |  |  |  |  |
|  |  | LDHA |  |  |  |  |
|  |  | LDLRAD3 |  |  |  |  |
|  |  | LEF1 |  |  |  |  |
|  |  | LGALS7B |  |  |  |  |
|  |  | LHFPL5 |  |  |  |  |
|  |  | LHX6 |  |  |  |  |
|  |  | LOC107985678 |  |  |  |  |
|  |  | LONRF3 |  |  |  |  |
|  |  | LPAR3 |  |  |  |  |
|  |  | LRATD1 |  |  |  |  |
|  |  | LRRC15 |  |  |  |  |
|  |  | LRWD1 |  |  |  |  |
|  |  | LSM12 |  |  |  |  |
|  |  | LTB4R |  |  |  |  |
|  |  | LTB4R2 |  |  |  |  |
|  |  | LY6D |  |  |  |  |
|  |  | LY6K |  |  |  |  |
|  |  | LYAR |  |  |  |  |
|  |  | MAGEB2 |  |  |  |  |
|  |  | MAGIX |  |  |  |  |
|  |  | MANEAL |  |  |  |  |
|  |  | MAOB |  |  |  |  |
|  |  | MAP1LC3B2 |  |  |  |  |
|  |  | MAP2K1 |  |  |  |  |
|  |  | MAP2K4 |  |  |  |  |
|  |  | MARCKSL1 |  |  |  |  |
|  |  | MARS2 |  |  |  |  |
|  |  | MCM2 |  |  |  |  |
|  |  | MCM3 |  |  |  |  |
|  |  | MCM4 |  |  |  |  |
|  |  | MCM6 |  |  |  |  |
|  |  | MCRIP1 |  |  |  |  |
|  |  | MEAF6 |  |  |  |  |
|  |  | MED20 |  |  |  |  |
|  |  | MEIS3 |  |  |  |  |
|  |  | METAP1D |  |  |  |  |
|  |  | METTL21A |  |  |  |  |
|  |  | MEX3B |  |  |  |  |
|  |  | MFNG |  |  |  |  |
|  |  | MFSD12 |  |  |  |  |
|  |  | MICOS10-NBL1 |  |  |  |  |
|  |  | MINDY4 |  |  |  |  |
|  |  | MIPEP |  |  |  |  |
|  |  | MIX23 |  |  |  |  |
|  |  | MLLT11 |  |  |  |  |
|  |  | MMP7 |  |  |  |  |
|  |  | MOK |  |  |  |  |
|  |  | MORN2 |  |  |  |  |
|  |  | MORN4 |  |  |  |  |
|  |  | MPP1 |  |  |  |  |
|  |  | MPPE1 |  |  |  |  |
|  |  | MRC2 |  |  |  |  |
|  |  | MRPL18 |  |  |  |  |
|  |  | MRPL3 |  |  |  |  |
|  |  | MRPL39 |  |  |  |  |
|  |  | MRPS23 |  |  |  |  |
|  |  | MRPS26 |  |  |  |  |
|  |  | MRPS35 |  |  |  |  |
|  |  | MSANTD3 |  |  |  |  |
|  |  | MSMB |  |  |  |  |
|  |  | MT1X |  |  |  |  |
|  |  | MTARC1 |  |  |  |  |
|  |  | MTHFD1L |  |  |  |  |
|  |  | MTHFS |  |  |  |  |
|  |  | MTRNR2L8 |  |  |  |  |
|  |  | MUC13 |  |  |  |  |
|  |  | MXRA8 |  |  |  |  |
|  |  | MYH3 |  |  |  |  |
|  |  | MYLK |  |  |  |  |
|  |  | NAAA |  |  |  |  |
|  |  | NAPSA |  |  |  |  |
|  |  | NARS2 |  |  |  |  |
|  |  | NAT10 |  |  |  |  |
|  |  | NDRG1 |  |  |  |  |
|  |  | NECAB2 |  |  |  |  |
|  |  | NECAP1 |  |  |  |  |
|  |  | NETO2 |  |  |  |  |
|  |  | NFIL3 |  |  |  |  |
|  |  | NGRN |  |  |  |  |
|  |  | NMB |  |  |  |  |
|  |  | NME2 |  |  |  |  |
|  |  | NOC2L |  |  |  |  |
|  |  | NOL10 |  |  |  |  |
|  |  | NOLC1 |  |  |  |  |
|  |  | NOP16 |  |  |  |  |
|  |  | NPHP4 |  |  |  |  |
|  |  | NPIPA2 |  |  |  |  |
|  |  | NPL |  |  |  |  |
|  |  | NPR3 |  |  |  |  |
|  |  | NPRL3 |  |  |  |  |
|  |  | NQO1 |  |  |  |  |
|  |  | NR1D1 |  |  |  |  |
|  |  | NRG1 |  |  |  |  |
|  |  | NSMF |  |  |  |  |
|  |  | NUBP1 |  |  |  |  |
|  |  | NUDC |  |  |  |  |
|  |  | NUDT15 |  |  |  |  |
|  |  | NXPH4 |  |  |  |  |
|  |  | ODC1 |  |  |  |  |
|  |  | OSBP2 |  |  |  |  |
|  |  | OSBPL5 |  |  |  |  |
|  |  | OSBPL6 |  |  |  |  |
|  |  | OSR2 |  |  |  |  |
|  |  | PABPC1L |  |  |  |  |
|  |  | PACC1 |  |  |  |  |
|  |  | PACRGL |  |  |  |  |
|  |  | PAQR6 |  |  |  |  |
|  |  | PAQR9 |  |  |  |  |
|  |  | PARP16 |  |  |  |  |
|  |  | PARVG |  |  |  |  |
|  |  | PASD1 |  |  |  |  |
|  |  | PBX3 |  |  |  |  |
|  |  | PCDHAC1 |  |  |  |  |
|  |  | PCOTH |  |  |  |  |
|  |  | PCP2 |  |  |  |  |
|  |  | PDCL3 |  |  |  |  |
|  |  | PDE2A |  |  |  |  |
|  |  | PDE6G |  |  |  |  |
|  |  | PDK1 |  |  |  |  |
|  |  | PDSS1 |  |  |  |  |
|  |  | PDXP |  |  |  |  |
|  |  | PEA15 |  |  |  |  |
|  |  | PEMT |  |  |  |  |
|  |  | PFDN2 |  |  |  |  |
|  |  | PFKFB3 |  |  |  |  |
|  |  | PGF |  |  |  |  |
|  |  | PHEX |  |  |  |  |
|  |  | PHKA1 |  |  |  |  |
|  |  | PIGX |  |  |  |  |
|  |  | PIK3AP1 |  |  |  |  |
|  |  | PIM1 |  |  |  |  |
|  |  | PINLYP |  |  |  |  |
|  |  | PINX1 |  |  |  |  |
|  |  | PITPNM2 |  |  |  |  |
|  |  | PLAT |  |  |  |  |
|  |  | PLCB2 |  |  |  |  |
|  |  | PLEKHG4 |  |  |  |  |
|  |  | PLIN2 |  |  |  |  |
|  |  | PLXND1 |  |  |  |  |
|  |  | PMAIP1 |  |  |  |  |
|  |  | PMP22 |  |  |  |  |
|  |  | PNO1 |  |  |  |  |
|  |  | POLR3G |  |  |  |  |
|  |  | POPDC3 |  |  |  |  |
|  |  | PORCN |  |  |  |  |
|  |  | POU3F1 |  |  |  |  |
|  |  | PPCDC |  |  |  |  |
|  |  | PPFIA4 |  |  |  |  |
|  |  | PPID |  |  |  |  |
|  |  | PPIL1 |  |  |  |  |
|  |  | PPP1R14C |  |  |  |  |
|  |  | PPP1R15A |  |  |  |  |
|  |  | PPP1R3C |  |  |  |  |
|  |  | PREX1 |  |  |  |  |
|  |  | PRKAR1B |  |  |  |  |
|  |  | PRKCE |  |  |  |  |
|  |  | PRMT3 |  |  |  |  |
|  |  | PRPS1 |  |  |  |  |
|  |  | PSAT1 |  |  |  |  |
|  |  | PSEN2 |  |  |  |  |
|  |  | PSG1 |  |  |  |  |
|  |  | PSG2 |  |  |  |  |
|  |  | PSG4 |  |  |  |  |
|  |  | PSG6 |  |  |  |  |
|  |  | PSMC3IP |  |  |  |  |
|  |  | PTCH2 |  |  |  |  |
|  |  | PTDSS1 |  |  |  |  |
|  |  | PTGES3 |  |  |  |  |
|  |  | PTGS1 |  |  |  |  |
|  |  | PTX3 |  |  |  |  |
|  |  | PUS1 |  |  |  |  |
|  |  | QDPR |  |  |  |  |
|  |  | QRICH2 |  |  |  |  |
|  |  | QSOX2 |  |  |  |  |
|  |  | RAB38 |  |  |  |  |
|  |  | RAB7B |  |  |  |  |
|  |  | RABEPK |  |  |  |  |
|  |  | RAMP1 |  |  |  |  |
|  |  | RBM14 |  |  |  |  |
|  |  | RBM19 |  |  |  |  |
|  |  | RERG |  |  |  |  |
|  |  | RGS2 |  |  |  |  |
|  |  | RGS4 |  |  |  |  |
|  |  | RINL |  |  |  |  |
|  |  | RNASE7 |  |  |  |  |
|  |  | RND1 |  |  |  |  |
|  |  | RNF183 |  |  |  |  |
|  |  | RNF187 |  |  |  |  |
|  |  | RNF32 |  |  |  |  |
|  |  | ROPN1L |  |  |  |  |
|  |  | RPA2 |  |  |  |  |
|  |  | RPF2 |  |  |  |  |
|  |  | RPIA |  |  |  |  |
|  |  | RPL22L1 |  |  |  |  |
|  |  | RPP40 |  |  |  |  |
|  |  | RRAD |  |  |  |  |
|  |  | RRAGD |  |  |  |  |
|  |  | RRP12 |  |  |  |  |
|  |  | RSRP1 |  |  |  |  |
|  |  | RUVBL1 |  |  |  |  |
|  |  | RWDD4 |  |  |  |  |
|  |  | RXYLT1 |  |  |  |  |
|  |  | S100P |  |  |  |  |
|  |  | S1PR1 |  |  |  |  |
|  |  | SAMD1 |  |  |  |  |
|  |  | SBDS |  |  |  |  |
|  |  | SBSN |  |  |  |  |
|  |  | SCFD2 |  |  |  |  |
|  |  | SCIN |  |  |  |  |
|  |  | SCLY |  |  |  |  |
|  |  | SDHAF3 |  |  |  |  |
|  |  | SELENOP |  |  |  |  |
|  |  | SEMA3G |  |  |  |  |
|  |  | SERPINB7 |  |  |  |  |
|  |  | SFXN4 |  |  |  |  |
|  |  | SH2D1B |  |  |  |  |
|  |  | SH2D2A |  |  |  |  |
|  |  | SH3BP5 |  |  |  |  |
|  |  | SHC3 |  |  |  |  |
|  |  | SHC4 |  |  |  |  |
|  |  | SHLD3 |  |  |  |  |
|  |  | SIGMAR1 |  |  |  |  |
|  |  | SIRPB2 |  |  |  |  |
|  |  | SLAMF9 |  |  |  |  |
|  |  | SLBP |  |  |  |  |
|  |  | SLC13A3 |  |  |  |  |
|  |  | SLC16A2 |  |  |  |  |
|  |  | SLC17A7 |  |  |  |  |
|  |  | SLC19A1 |  |  |  |  |
|  |  | SLC25A15 |  |  |  |  |
|  |  | SLC25A20 |  |  |  |  |
|  |  | SLC25A4 |  |  |  |  |
|  |  | SLC25A45 |  |  |  |  |
|  |  | SLC29A1 |  |  |  |  |
|  |  | SLC34A3 |  |  |  |  |
|  |  | SLC35E4 |  |  |  |  |
|  |  | SLC35G1 |  |  |  |  |
|  |  | SLC38A5 |  |  |  |  |
|  |  | SLC39A3 |  |  |  |  |
|  |  | SLC5A1 |  |  |  |  |
|  |  | SLC5A6 |  |  |  |  |
|  |  | SLC6A12 |  |  |  |  |
|  |  | SLC6A8 |  |  |  |  |
|  |  | SLC9A9 |  |  |  |  |
|  |  | SMAD9 |  |  |  |  |
|  |  | SMCO4 |  |  |  |  |
|  |  | SMIM10 |  |  |  |  |
|  |  | SMIM20 |  |  |  |  |
|  |  | SMIM4 |  |  |  |  |
|  |  | SMKR1 |  |  |  |  |
|  |  | SMS |  |  |  |  |
|  |  | SMYD2 |  |  |  |  |
|  |  | SNAI1 |  |  |  |  |
|  |  | SNRPA1 |  |  |  |  |
|  |  | SNRPD1 |  |  |  |  |
|  |  | SORCS3 |  |  |  |  |
|  |  | SOWAHD |  |  |  |  |
|  |  | SPATA5 |  |  |  |  |
|  |  | SPATA6 |  |  |  |  |
|  |  | SPG21 |  |  |  |  |
|  |  | SPHK1 |  |  |  |  |
|  |  | SPIN2B |  |  |  |  |
|  |  | SPINDOC |  |  |  |  |
|  |  | SPRR2E |  |  |  |  |
|  |  | SRM |  |  |  |  |
|  |  | SRPRB |  |  |  |  |
|  |  | SRSF1 |  |  |  |  |
|  |  | STAC |  |  |  |  |
|  |  | STC1 |  |  |  |  |
|  |  | STC2 |  |  |  |  |
|  |  | STK39 |  |  |  |  |
|  |  | STRAP |  |  |  |  |
|  |  | SUMO4 |  |  |  |  |
|  |  | SUN3 |  |  |  |  |
|  |  | SURF2 |  |  |  |  |
|  |  | SWI5 |  |  |  |  |
|  |  | SYTL3 |  |  |  |  |
|  |  | TBX1 |  |  |  |  |
|  |  | TBXAS1 |  |  |  |  |
|  |  | TCOF1 |  |  |  |  |
|  |  | TCTA |  |  |  |  |
|  |  | TECPR2 |  |  |  |  |
|  |  | TERT |  |  |  |  |
|  |  | TEX13C |  |  |  |  |
|  |  | TEX30 |  |  |  |  |
|  |  | TFCP2L1 |  |  |  |  |
|  |  | TFDP1 |  |  |  |  |
|  |  | TFPI2 |  |  |  |  |
|  |  | THNSL2 |  |  |  |  |
|  |  | TIGD6 |  |  |  |  |
|  |  | TIMM10B |  |  |  |  |
|  |  | TM4SF19 |  |  |  |  |
|  |  | TMCC2 |  |  |  |  |
|  |  | TMEM132B |  |  |  |  |
|  |  | TMEM150A |  |  |  |  |
|  |  | TMEM158 |  |  |  |  |
|  |  | TMEM242 |  |  |  |  |
|  |  | TMEM25 |  |  |  |  |
|  |  | TMEM251 |  |  |  |  |
|  |  | TMEM38A |  |  |  |  |
|  |  | TMEM38B |  |  |  |  |
|  |  | TMEM70 |  |  |  |  |
|  |  | TMEM74B |  |  |  |  |
|  |  | TMEM9B |  |  |  |  |
|  |  | TMSB15A |  |  |  |  |
|  |  | TNF |  |  |  |  |
|  |  | TNFAIP8L3 |  |  |  |  |
|  |  | TNFRSF10D |  |  |  |  |
|  |  | TNFRSF8 |  |  |  |  |
|  |  | TNFSF9 |  |  |  |  |
|  |  | TNNT2 |  |  |  |  |
|  |  | TOMM34 |  |  |  |  |
|  |  | TOMM40 |  |  |  |  |
|  |  | TOMM6 |  |  |  |  |
|  |  | TOP1MT |  |  |  |  |
|  |  | TPBG |  |  |  |  |
|  |  | TPD52L1 |  |  |  |  |
|  |  | TRAP1 |  |  |  |  |
|  |  | TREML2 |  |  |  |  |
|  |  | TRIM9 |  |  |  |  |
|  |  | TRMT1 |  |  |  |  |
|  |  | TRMT61A |  |  |  |  |
|  |  | TRPV1 |  |  |  |  |
|  |  | TSEN2 |  |  |  |  |
|  |  | TTC27 |  |  |  |  |
|  |  | TTC36 |  |  |  |  |
|  |  | TUSC3 |  |  |  |  |
|  |  | TXNIP |  |  |  |  |
|  |  | TYRO3 |  |  |  |  |
|  |  | UBB |  |  |  |  |
|  |  | UBC |  |  |  |  |
|  |  | UBE2D4 |  |  |  |  |
|  |  | UBE2F |  |  |  |  |
|  |  | UBE2V1 |  |  |  |  |
|  |  | UBQLN4 |  |  |  |  |
|  |  | UBTF |  |  |  |  |
|  |  | UHRF1 |  |  |  |  |
|  |  | UNG |  |  |  |  |
|  |  | UPK2 |  |  |  |  |
|  |  | UPP1 |  |  |  |  |
|  |  | USP47 |  |  |  |  |
|  |  | VARS1 |  |  |  |  |
|  |  | VDAC1 |  |  |  |  |
|  |  | VGF |  |  |  |  |
|  |  | WDR12 |  |  |  |  |
|  |  | WDR4 |  |  |  |  |
|  |  | WDR61 |  |  |  |  |
|  |  | WDR76 |  |  |  |  |
|  |  | WFDC3 |  |  |  |  |
|  |  | WHRN |  |  |  |  |
|  |  | WNT10B |  |  |  |  |
|  |  | XKR9 |  |  |  |  |
|  |  | XYLB |  |  |  |  |
|  |  | ZBTB8B |  |  |  |  |
|  |  | ZDHHC11 |  |  |  |  |
|  |  | ZFAND2A |  |  |  |  |
|  |  | ZHX1-C8orf76 |  |  |  |  |
|  |  | ZMYND12 |  |  |  |  |
|  |  | ZNF365 |  |  |  |  |
|  |  | ZNF586 |  |  |  |  |
|  |  | ZNF692 |  |  |  |  |
|  |  | ZPR1 |  |  |  |  |

**Table S8.Top 10 enriched GO-BP pathways for each PSC subgroup**

| GO-BP TOP 10 | | | | | | |  |  |
| --- | --- | --- | --- | --- | --- | --- | --- | --- |
| Cluster0 |  |  |  |  | | | | |
| **ID** | **Description** | **GeneRatio** | | | **pvalue** | **geneID** | |  |
| GO:0032496 | response to lipopolysaccharide | 18/113 | | | 1.36031E-12 | CSF3/CXCL8/CXCL5/ICAM1/CD68/CXCL1/IL6/CXCL3/ADM/MGST1/PTGES/CXCL2/PTGS2/NFKBIA/GJA1/TNFAIP3/THBD/AKAP12 | |  |
| GO:0002237 | response to molecule of bacterial origin | 18/113 | | | 3.96965E-12 | CSF3/CXCL8/CXCL5/ICAM1/CD68/CXCL1/IL6/CXCL3/ADM/MGST1/PTGES/CXCL2/PTGS2/NFKBIA/GJA1/TNFAIP3/THBD/AKAP12 | |  |
| GO:0071216 | cellular response to biotic stimulus | 12/113 | | | 2.85272E-08 | CSF3/CXCL8/CXCL5/ICAM1/CD68/CXCL1/IL6/CXCL3/CXCL2/NFKBIA/TNFAIP3/DDIT3 | |  |
| GO:0071222 | cellular response to lipopolysaccharide | 11/113 | | | 4.88981E-08 | CSF3/CXCL8/CXCL5/ICAM1/CD68/CXCL1/IL6/CXCL3/CXCL2/NFKBIA/TNFAIP3 | |  |
| GO:0071219 | cellular response to molecule of bacterial origin | 11/113 | | | 9.49885E-08 | CSF3/CXCL8/CXCL5/ICAM1/CD68/CXCL1/IL6/CXCL3/CXCL2/NFKBIA/TNFAIP3 | |  |
| GO:1904018 | positive regulation of vasculature development | 11/113 | | | 1.68866E-07 | CXCL8/HMOX1/FGF2/SOD2/VEGFA/ADM/ANXA1/CYP1B1/PTGS2/HK2/F3 | |  |
| GO:0051384 | response to glucocorticoid | 9/113 | | | 2.48278E-07 | ICAM1/IL6/ADM/ANXA1/PTGS2/DUSP1/SERPINF1/PAPPA/FOSL1 | |  |
| GO:0033138 | positive regulation of peptidyl-serine phosphorylation | 8/113 | | | 2.96473E-07 | CSF3/IL11/CD44/VEGFA/IL6/CAMK1/PTGS2/FNIP2 | |  |
| GO:0061041 | regulation of wound healing | 9/113 | | | 3.12009E-07 | FGF2/SERPINB2/PDPN/ANXA1/GJA1/TNFAIP3/THBD/F3/C1QTNF1 | |  |
| GO:1901342 | regulation of vasculature development | 14/113 | | | 4.28082E-07 | CXCL8/HMOX1/FGF2/SOD2/VEGFA/IL6/ADM/ANXA1/CYP1B1/PTGS2/TNFAIP3/HK2/SERPINF1/F3 | |  |
|  |  |  | | |  |  | |  |
| Cluster1 |  |  | | |  |  | |  |
| **ID** | **Description** | **GeneRatio** | | | **pvalue** | **geneID** | |  |
| GO:0030198 | extracellular matrix organization | 41/190 | | | 8.32727E-30 | MMP11/COL1A2/COL3A1/COL1A1/FN1/COL18A1/BMP1/FMOD/TIMP2/SULF2/NOTCH1/COL5A1/LUM/PXDN/THBS1/COL6A3/COL8A1/ITGA11/COL16A1/CTSK/AEBP1/ITGA1/COL15A1/COL12A1/COMP/COL10A1/COL5A2/HTRA1/MMP14/LRP1/SFRP2/ECM2/LAMB1/LTBP3/GAS6/ADAMTS2/SULF1/MFAP2/COLGALT1/FAP/ANTXR1 | |  |
| GO:0043062 | extracellular structure organization | 41/190 | | | 9.21603E-30 | MMP11/COL1A2/COL3A1/COL1A1/FN1/COL18A1/BMP1/FMOD/TIMP2/SULF2/NOTCH1/COL5A1/LUM/PXDN/THBS1/COL6A3/COL8A1/ITGA11/COL16A1/CTSK/AEBP1/ITGA1/COL15A1/COL12A1/COMP/COL10A1/COL5A2/HTRA1/MMP14/LRP1/SFRP2/ECM2/LAMB1/LTBP3/GAS6/ADAMTS2/SULF1/MFAP2/COLGALT1/FAP/ANTXR1 | |  |
| GO:0030199 | collagen fibril organization | 15/190 | | | 5.31877E-18 | MMP11/COL1A2/COL3A1/COL1A1/FMOD/COL5A1/LUM/PXDN/AEBP1/COL12A1/COMP/COL5A2/SFRP2/ADAMTS2/COLGALT1 | |  |
| GO:0032963 | collagen metabolic process | 15/190 | | | 4.8702E-13 | MMP11/COL1A2/COL1A1/MRC2/COL5A1/CTSB/TGFB3/CYGB/CTSK/COL15A1/WNT4/MMP14/P3H1/ADAMTS2/FAP | |  |
| GO:0031589 | cell-substrate adhesion | 23/190 | | | 1.83638E-12 | COL3A1/COL1A1/FN1/THY1/NOTCH1/THBS1/COL8A1/ITGA11/COL16A1/JUP/ITGA1/WNT4/MMP14/LRP1/ECM2/LAMB1/GAS6/EPHA3/ANTXR1/SORBS3/LDB1/MAP4K4/DOCK1 | |  |
| GO:0001503 | ossification | 24/190 | | | 4.45024E-12 | COL1A2/COL1A1/BMP1/MRC2/NOTCH1/IGFBP5/TGFB3/ITGA11/CTSK/WNT4/MGP/COMP/COL5A2/MMP14/DHRS3/EGR2/ANKH/SFRP2/FAM20C/LTBP3/INPPL1/CTHRC1/CLEC11A/SNAI1 | |  |
| GO:0010810 | regulation of cell-substrate adhesion | 16/190 | | | 6.66008E-10 | COL1A1/FN1/THY1/NOTCH1/THBS1/COL8A1/COL16A1/JUP/WNT4/MMP14/LRP1/ECM2/EPHA3/LDB1/MAP4K4/DOCK1 | |  |
| GO:0007044 | cell-substrate junction assembly | 11/190 | | | 4.71736E-09 | FN1/THY1/THBS1/COL16A1/WNT4/MMP14/LRP1/EPHA3/LDB1/CD151/MAP4K4 | |  |
| GO:0150115 | cell-substrate junction organization | 11/190 | | | 6.49494E-09 | FN1/THY1/THBS1/COL16A1/WNT4/MMP14/LRP1/EPHA3/LDB1/CD151/MAP4K4 | |  |
| GO:0007160 | cell-matrix adhesion | 15/190 | | | 1.23513E-08 | COL3A1/FN1/THY1/THBS1/ITGA11/COL16A1/JUP/ITGA1/WNT4/MMP14/LRP1/ECM2/EPHA3/LDB1/MAP4K4 | |  |
|  |  |  | | |  |  | |  |
| Cluster2 |  |  | | |  |  | |  |
| **ID** | **Description** | **GeneRatio** | | | **pvalue** | **geneID** | |  |
| GO:0003018 | vascular process in circulatory system | 7/59 | | | 2.06195E-06 | EDNRB/EDNRA/GPR4/ANGPT1/AVPR1A/ADRA2C/PTP4A3 | |  |
| GO:0032355 | response to estradiol | 6/59 | | | 5.09555E-06 | TFPI/NQO1/POSTN/NR2F2/CD4/TXNIP | |  |
| GO:0045444 | fat cell differentiation | 6/59 | | | 9.09555E-05 | ADIRF/FABP4/GRK5/WNT5A/CCDC3/LPL | |  |
| GO:0042542 | response to hydrogen peroxide | 5/59 | | | 9.26925E-05 | TRPC6/NQO1/HGF/PDE8A/TXNIP | |  |
| GO:0031644 | regulation of nervous system process | 5/59 | | | 9.88399E-05 | EDNRB/HGF/AVPR1A/TNFRSF21/ADRA2C | |  |
| GO:0042310 | vasoconstriction | 4/59 | | | 0.000103539 | EDNRB/EDNRA/AVPR1A/ADRA2C | |  |
| GO:0051480 | regulation of cytosolic calcium ion concentration | 7/59 | | | 0.000119972 | EDNRB/TRPC6/EDNRA/GPR4/WNT5A/AVPR1A/CD4 | |  |
| GO:0097756 | negative regulation of blood vessel diameter | 4/59 | | | 0.0001513 | EDNRB/EDNRA/AVPR1A/ADRA2C | |  |
| GO:0040013 | negative regulation of locomotion | 7/59 | | | 0.000230414 | IFITM1/IL24/ADAMTS9/ATP1B2/WNT5A/NR2F2/BST2 | |  |
| GO:0051271 | negative regulation of cellular component movement | 7/59 | | | 0.000241222 | IFITM1/IL24/ADAMTS9/ATP1B2/WNT5A/NR2F2/BST2 | |  |
|  |  |  | | |  |  | |  |
| Cluster3 |  |  | | |  |  | |  |
| **ID** | **Description** | **GeneRatio** | | | **pvalue** | **geneID** | |  |
| GO:0070482 | response to oxygen levels | 6/19 | | | 1.77268E-06 | HILPDA/NAMPT/EPAS1/ADM/CDKN1A/CXCR4 | |  |
| GO:1901342 | regulation of vasculature development | 6/19 | | | 3.43603E-06 | SOD2/GPR4/ADM/CXCL8/ADAMTS9/CXCR4 | |  |
| GO:0036293 | response to decreased oxygen levels | 5/19 | | | 2.6522E-05 | HILPDA/NAMPT/EPAS1/ADM/CXCR4 | |  |
| GO:0045765 | regulation of angiogenesis | 5/19 | | | 3.94013E-05 | GPR4/ADM/CXCL8/ADAMTS9/CXCR4 | |  |
| GO:0009266 | response to temperature stimulus | 4/19 | | | 7.59573E-05 | ADM/CDKN1A/NFKBIA/CXCR4 | |  |
| GO:1904018 | positive regulation of vasculature development | 4/19 | | | 7.85161E-05 | SOD2/ADM/CXCL8/CXCR4 | |  |
| GO:0007568 | aging | 4/19 | | | 0.000254374 | SOD2/NAMPT/ADM/CDKN1A | |  |
| GO:0032496 | response to lipopolysaccharide | 4/19 | | | 0.000303038 | IL24/ADM/CXCL8/NFKBIA | |  |
| GO:0006516 | glycoprotein catabolic process | 2/19 | | | 0.000332257 | NEU4/ADAMTS9 | |  |
| GO:0002237 | response to molecule of bacterial origin | 4/19 | | | 0.000386083 | IL24/ADM/CXCL8/NFKBIA | |  |
|  |  |  | | |  |  | |  |
| Cluster4 |  |  | | |  |  | |  |
| **ID** | **Description** | **GeneRatio** | | | **pvalue** | **geneID** | |  |
| GO:0030198 | extracellular matrix organization | 14/72 | | | 2.45482E-10 | DPT/COL6A3/SFRP2/CYP1B1/CREB3L1/TGFBI/SULF1/NID2/COL15A1/LOXL3/ADAMTS12/ADTRP/FBN1/HTRA1 | |  |
| GO:0043062 | extracellular structure organization | 14/72 | | | 2.53738E-10 | DPT/COL6A3/SFRP2/CYP1B1/CREB3L1/TGFBI/SULF1/NID2/COL15A1/LOXL3/ADAMTS12/ADTRP/FBN1/HTRA1 | |  |
| GO:0090287 | regulation of cellular response to growth factor stimulus | 10/72 | | | 2.70493E-07 | SFRP2/CREB3L1/NREP/SULF1/MYOF/MSX1/ADAMTS12/ATP2B4/FBN1/HTRA1 | |  |
| GO:0090288 | negative regulation of cellular response to growth factor stimulus | 7/72 | | | 6.01515E-06 | SFRP2/CREB3L1/SULF1/ADAMTS12/ATP2B4/FBN1/HTRA1 | |  |
| GO:0030510 | regulation of BMP signaling pathway | 5/72 | | | 2.81542E-05 | SFRP2/SULF1/MSX1/FBN1/HTRA1 | |  |
| GO:0071772 | response to BMP | 6/72 | | | 4.38201E-05 | SFRP2/SULF1/MSX1/ADAMTS12/FBN1/HTRA1 | |  |
| GO:0071773 | cellular response to BMP stimulus | 6/72 | | | 4.38201E-05 | SFRP2/SULF1/MSX1/ADAMTS12/FBN1/HTRA1 | |  |
| GO:0050680 | negative regulation of epithelial cell proliferation | 6/72 | | | 5.50211E-05 | SFRP2/SULF1/MAGED1/DLG1/SCG2/CCL2 | |  |
| GO:0030336 | negative regulation of cell migration | 8/72 | | | 5.51484E-05 | SFRP2/CYP1B1/SULF1/BCR/ATP2B4/ADTRP/SRGAP1/CCL2 | |  |
| GO:0030199 | collagen fibril organization | 4/72 | | | 5.74029E-05 | DPT/SFRP2/CYP1B1/LOXL3 | |  |
|  |  |  | | |  |  | |  |
| Cluster5 |  |  | | |  |  | |  |
| **ID** | **Description** | **GeneRatio** | | | **pvalue** | **geneID** | |  |
| GO:0030198 | extracellular matrix organization | 37/144 | | | 6.02765E-30 | COL7A1/FN1/COL12A1/TGFBI/SERPINE1/LRP1/LOXL2/COL5A1/COL6A3/ANXA2/MMP2/TGFB1/COL1A2/MMP1/VCAN/FAP/MMP3/ITGA2/FBN1/COL5A2/ITGA5/COL8A1/MMP10/COL15A1/P4HA1/THBS1/FOXF2/FSCN1/LOX/MFAP2/PLOD3/ADAM12/GREM1/ADAMTS6/LOXL3/HSPG2/PDGFRA | |  |
| GO:0043062 | extracellular structure organization | 37/144 | | | 6.61146E-30 | COL7A1/FN1/COL12A1/TGFBI/SERPINE1/LRP1/LOXL2/COL5A1/COL6A3/ANXA2/MMP2/TGFB1/COL1A2/MMP1/VCAN/FAP/MMP3/ITGA2/FBN1/COL5A2/ITGA5/COL8A1/MMP10/COL15A1/P4HA1/THBS1/FOXF2/FSCN1/LOX/MFAP2/PLOD3/ADAM12/GREM1/ADAMTS6/LOXL3/HSPG2/PDGFRA | |  |
| GO:0030199 | collagen fibril organization | 11/144 | | | 3.12372E-13 | COL12A1/LOXL2/COL5A1/ANXA2/COL1A2/COL5A2/P4HA1/LOX/PLOD3/GREM1/LOXL3 | |  |
| GO:0032963 | collagen metabolic process | 12/144 | | | 6.28764E-11 | MRC2/COL5A1/MMP2/TGFB1/COL1A2/MMP1/FAP/MMP3/ITGA2/MMP10/COL15A1/PLOD3 | |  |
| GO:0001706 | endoderm formation | 9/144 | | | 3.22451E-10 | COL7A1/FN1/COL12A1/COL5A1/MMP2/COL5A2/ITGA5/COL8A1/DUSP1 | |  |
| GO:0035987 | endodermal cell differentiation | 8/144 | | | 1.93471E-09 | COL7A1/FN1/COL12A1/COL5A1/MMP2/COL5A2/ITGA5/COL8A1 | |  |
| GO:0007369 | gastrulation | 13/144 | | | 2.26628E-09 | COL7A1/FN1/COL12A1/COL5A1/MMP2/GJA1/ITGA2/COL5A2/ITGA5/COL8A1/POGLUT1/MYADM/DUSP1 | |  |
| GO:0001704 | formation of primary germ layer | 11/144 | | | 3.6398E-09 | COL7A1/FN1/COL12A1/COL5A1/MMP2/GJA1/ITGA2/COL5A2/ITGA5/COL8A1/DUSP1 | |  |
| GO:0007492 | endoderm development | 9/144 | | | 8.93892E-09 | COL7A1/FN1/COL12A1/COL5A1/MMP2/COL5A2/ITGA5/COL8A1/DUSP1 | |  |
| GO:0030574 | collagen catabolic process | 7/144 | | | 6.35173E-08 | MRC2/MMP2/MMP1/FAP/MMP3/MMP10/COL15A1 | |  |
|  |  |  | | |  |  | |  |
| Cluster6 |  |  | | |  |  | |  |
| **ID** | **Description** | **GeneRatio** | | | **pvalue** | **geneID** | |  |
| GO:0009914 | hormone transport | 40/365 | | | 6.04365E-21 | NEUROD1/FOXA2/NKX6-1/KCNJ11/PTPRN/EDN3/CACNA2D2/ISL1/G6PC2/VGF/VAMP8/CHGA/PDX1/RIMS2/RFX6/SCG5/ABCC8/ICA1/STX1A/UCN3/SNAP25/GCK/SLC30A8/PTPRN2/BAIAP3/EFNA5/TTR/SYT7/CPE/RPH3AL/MAFA/PCLO/ABAT/SLC7A8/ADCYAP1/SPP1/CACNA1A/RAB11FIP1/SLC25A4/CAMK2G | |  |
| GO:0046879 | hormone secretion | 38/365 | | | 1.36483E-19 | NEUROD1/FOXA2/NKX6-1/KCNJ11/PTPRN/EDN3/CACNA2D2/ISL1/G6PC2/VGF/VAMP8/CHGA/PDX1/RIMS2/RFX6/SCG5/ABCC8/ICA1/STX1A/UCN3/SNAP25/GCK/SLC30A8/PTPRN2/BAIAP3/EFNA5/SYT7/CPE/RPH3AL/MAFA/PCLO/ABAT/ADCYAP1/SPP1/CACNA1A/RAB11FIP1/SLC25A4/CAMK2G | |  |
| GO:0030073 | insulin secretion | 32/365 | | | 1.71482E-19 | NEUROD1/FOXA2/NKX6-1/KCNJ11/PTPRN/CACNA2D2/ISL1/G6PC2/VGF/CHGA/PDX1/RIMS2/RFX6/ABCC8/ICA1/STX1A/UCN3/SNAP25/GCK/SLC30A8/PTPRN2/BAIAP3/EFNA5/SYT7/RPH3AL/MAFA/PCLO/ABAT/ADCYAP1/CACNA1A/SLC25A4/CAMK2G | |  |
| GO:0009306 | protein secretion | 45/365 | | | 3.12774E-19 | NEUROD1/FOXA2/NKX6-1/KCNJ11/PTPRN/CACNA2D2/ISL1/G6PC2/RAB3B/VGF/CHGA/PDX1/RIMS2/RFX6/SORL1/ABCC8/ICA1/STX1A/SCAMP5/UCN3/SNAP25/GCK/PARD6A/CADM1/LLGL2/SLC30A8/SYT4/PTPRN2/BAIAP3/RAB3C/EFNA5/SYT7/RPH3AL/PAM/SCG2/MAFA/PCLO/ABAT/HDAC9/ADCYAP1/CACNA1A/EZR/RAB11FIP1/SLC25A4/CAMK2G | |  |
| GO:0035592 | establishment of protein localization to extracellular region | 45/365 | | | 3.4058E-19 | NEUROD1/FOXA2/NKX6-1/KCNJ11/PTPRN/CACNA2D2/ISL1/G6PC2/RAB3B/VGF/CHGA/PDX1/RIMS2/RFX6/SORL1/ABCC8/ICA1/STX1A/SCAMP5/UCN3/SNAP25/GCK/PARD6A/CADM1/LLGL2/SLC30A8/SYT4/PTPRN2/BAIAP3/RAB3C/EFNA5/SYT7/RPH3AL/PAM/SCG2/MAFA/PCLO/ABAT/HDAC9/ADCYAP1/CACNA1A/EZR/RAB11FIP1/SLC25A4/CAMK2G | |  |
| GO:0071692 | protein localization to extracellular region | 45/365 | | | 6.14232E-19 | NEUROD1/FOXA2/NKX6-1/KCNJ11/PTPRN/CACNA2D2/ISL1/G6PC2/RAB3B/VGF/CHGA/PDX1/RIMS2/RFX6/SORL1/ABCC8/ICA1/STX1A/SCAMP5/UCN3/SNAP25/GCK/PARD6A/CADM1/LLGL2/SLC30A8/SYT4/PTPRN2/BAIAP3/RAB3C/EFNA5/SYT7/RPH3AL/PAM/SCG2/MAFA/PCLO/ABAT/HDAC9/ADCYAP1/CACNA1A/EZR/RAB11FIP1/SLC25A4/CAMK2G | |  |
| GO:0030072 | peptide hormone secretion | 34/365 | | | 7.40636E-19 | NEUROD1/FOXA2/NKX6-1/KCNJ11/PTPRN/EDN3/CACNA2D2/ISL1/G6PC2/VGF/CHGA/PDX1/RIMS2/RFX6/ABCC8/ICA1/STX1A/UCN3/SNAP25/GCK/SLC30A8/PTPRN2/BAIAP3/EFNA5/SYT7/CPE/RPH3AL/MAFA/PCLO/ABAT/ADCYAP1/CACNA1A/SLC25A4/CAMK2G | |  |
| GO:0046883 | regulation of hormone secretion | 30/365 | | | 8.30157E-15 | NEUROD1/FOXA2/NKX6-1/KCNJ11/EDN3/CACNA2D2/ISL1/G6PC2/VAMP8/CHGA/PDX1/RFX6/SCG5/ABCC8/ICA1/STX1A/UCN3/SNAP25/GCK/SLC30A8/BAIAP3/EFNA5/SYT7/RPH3AL/ABAT/ADCYAP1/SPP1/CACNA1A/RAB11FIP1/SLC25A4 | |  |
| GO:0050708 | regulation of protein secretion | 34/365 | | | 1.18531E-14 | NEUROD1/FOXA2/NKX6-1/KCNJ11/CACNA2D2/ISL1/G6PC2/CHGA/PDX1/RFX6/SORL1/ABCC8/ICA1/STX1A/SCAMP5/UCN3/SNAP25/GCK/PARD6A/CADM1/LLGL2/SLC30A8/SYT4/BAIAP3/EFNA5/SYT7/RPH3AL/PAM/ABAT/HDAC9/CACNA1A/EZR/RAB11FIP1/SLC25A4 | |  |
| GO:0002791 | regulation of peptide secretion | 35/365 | | | 2.15522E-14 | NEUROD1/FOXA2/NKX6-1/KCNJ11/CACNA2D2/ISL1/G6PC2/CHGA/PDX1/RFX6/SORL1/ABCC8/ICA1/STX1A/SCAMP5/UCN3/SNAP25/GCK/PARD6A/CADM1/LLGL2/SLC30A8/SYT4/BAIAP3/EFNA5/SYT7/RPH3AL/PAM/ABAT/HDAC9/ADCYAP1/CACNA1A/EZR/RAB11FIP1/SLC25A4 | |  |

**Table S9.Top 10 enriched KEGG pathways for each PSC subgroup**

| KEGG TOP 10 | | | | |
| --- | --- | --- | --- | --- |
| Cluster0 |  |  |  |  |
| **ID** | **Description** | **GeneRatio** | **pvalue** | **geneID** |
| hsa04657 | IL-17 signaling pathway | 12/79 | 3.27894E-11 | CSF3/CXCL8/CXCL5/CXCL1/IL6/CXCL3/CXCL2/PTGS2/NFKBIA/TNFAIP3/MMP1/FOSL1 |
| hsa04668 | TNF signaling pathway | 11/79 | 6.84842E-09 | CXCL5/ICAM1/CXCL1/IL6/CXCL3/CXCL2/PTGS2/NFKBIA/TNFAIP3/MAP3K8/MAP2K3 |
| hsa05323 | Rheumatoid arthritis | 10/79 | 8.89084E-09 | CXCL8/IL11/CXCL5/ICAM1/VEGFA/CXCL1/IL6/CXCL3/CXCL2/MMP1 |
| hsa05417 | Lipid and atherosclerosis | 12/79 | 4.01517E-07 | CXCL8/SOD2/ICAM1/CXCL1/IL6/CXCL3/CXCL2/NFKBIA/POU2F2/MMP1/DDIT3/MAP2K3 |
| hsa04064 | NF-kappa B signaling pathway | 8/79 | 3.9146E-06 | CXCL8/ICAM1/CXCL1/CXCL3/CXCL2/PTGS2/NFKBIA/TNFAIP3 |
| hsa05167 | Kaposi sarcoma-associated herpesvirus infection | 10/79 | 8.64792E-06 | CXCL8/FGF2/ICAM1/VEGFA/CXCL1/IL6/CXCL3/CXCL2/PTGS2/NFKBIA |
| hsa05134 | Legionellosis | 6/79 | 9.52777E-06 | CXCL8/CXCL1/IL6/CXCL3/CXCL2/NFKBIA |
| hsa04060 | Cytokine-cytokine receptor interaction | 11/79 | 6.18133E-05 | CSF3/IL33/INHBA/CXCL8/IL11/CXCL5/CXCL1/IL6/CXCL3/CXCL2/IL32 |
| hsa01232 | Nucleotide metabolism | 6/79 | 0.000104528 | UPP1/PNP/AMPD3/ADK/AK4/NT5E |
| hsa04061 | Viral protein interaction with cytokine and cytokine receptor | 6/79 | 0.000256251 | CXCL8/CXCL5/CXCL1/IL6/CXCL3/CXCL2 |
|  |  |  |  |  |
| Cluster1 |  |  |  |  |
| **ID** | **Description** | **GeneRatio** | **pvalue** | **geneID** |
| hsa04974 | Protein digestion and absorption | 12/96 | 1.12659E-09 | COL1A2/COL3A1/COL1A1/COL18A1/COL5A1/COL6A3/COL8A1/COL16A1/COL15A1/COL12A1/COL10A1/COL5A2 |
| hsa04512 | ECM-receptor interaction | 9/96 | 4.44098E-07 | COL1A2/COL1A1/FN1/THBS1/COL6A3/ITGA11/ITGA1/COMP/LAMB1 |
| hsa04820 | Cytoskeleton in muscle cells | 13/96 | 1.16436E-06 | COL1A2/COL3A1/COL1A1/FN1/COL5A1/THBS1/COL6A3/ITGA11/PDLIM2/JUP/ITGA1/COMP/COL5A2 |
| hsa04510 | Focal adhesion | 10/96 | 6.57255E-05 | COL1A2/COL1A1/FN1/THBS1/COL6A3/ITGA11/ITGA1/COMP/LAMB1/DOCK1 |
| hsa04142 | Lysosome | 8/96 | 8.75822E-05 | CTSB/CTSK/NPC2/NAGLU/CTSF/CTSZ/GAA/HEXA |
| hsa05165 | Human papillomavirus infection | 12/96 | 0.000241259 | COL1A2/COL1A1/FN1/NOTCH1/THBS1/COL6A3/ITGA11/ITGA1/WNT4/COMP/LAMB1/CHD4 |
| hsa04360 | Axon guidance | 8/96 | 0.000838142 | UNC5B/WNT4/SLIT2/EPHA3/PLXNB2/ENAH/EFNB1/NFATC2 |
| hsa05146 | Amoebiasis | 6/96 | 0.000855996 | COL1A2/COL3A1/COL1A1/FN1/TGFB3/LAMB1 |
| hsa05144 | Malaria | 4/96 | 0.002047518 | THBS1/TGFB3/COMP/LRP1 |
| hsa04933 | AGE-RAGE signaling pathway in diabetic complications | 5/96 | 0.004727628 | COL1A2/COL3A1/COL1A1/FN1/TGFB3 |
|  |  |  |  |  |
| Cluster2 |  |  |  |  |
| **ID** | **Description** | **GeneRatio** | **pvalue** | **geneID** |
| hsa04022 | cGMP-PKG signaling pathway | 6/40 | 9.02446E-05 | ADCY3/EDNRB/TRPC6/EDNRA/ATP1B2/ADRA2C |
| hsa04020 | Calcium signaling pathway | 7/40 | 0.000122742 | ADCY3/EDNRB/HGF/EDNRA/CYSLTR2/PDE1A/AVPR1A |
| hsa05032 | Morphine addiction | 4/40 | 0.000721669 | ADCY3/GRK5/PDE1A/PDE8A |
| hsa04916 | Melanogenesis | 4/40 | 0.001067231 | ADCY3/EDNRB/WNT6/WNT5A |
| hsa04918 | Thyroid hormone synthesis | 3/40 | 0.004617809 | ADCY3/GPX3/ATP1B2 |
| hsa04934 | Cushing syndrome | 4/40 | 0.005075007 | ADCY3/WNT6/WNT5A/PDE8A |
| hsa05225 | Hepatocellular carcinoma | 4/40 | 0.00702002 | NQO1/HGF/WNT6/WNT5A |
| hsa05208 | Chemical carcinogenesis - reactive oxygen species | 4/40 | 0.018464954 | NDUFA4L2/NQO1/HGF/NDUFB1 |
| hsa00230 | Purine metabolism | 3/40 | 0.019805653 | ADCY3/PDE1A/PDE8A |
| hsa04270 | Vascular smooth muscle contraction | 3/40 | 0.022329839 | ADCY3/EDNRA/AVPR1A |
|  |  |  |  |  |
| Cluster3 |  |  |  |  |
| **ID** | **Description** | **GeneRatio** | **pvalue** | **geneID** |
| hsa05163 | Human cytomegalovirus infection | 4/13 | 0.000247398 | CXCL8/CDKN1A/NFKBIA/CXCR4 |
| hsa04061 | Viral protein interaction with cytokine and cytokine receptor | 3/13 | 0.00036926 | IL24/CXCL8/CXCR4 |
| hsa05161 | Hepatitis B | 3/13 | 0.001533515 | CXCL8/CDKN1A/NFKBIA |
| hsa05219 | Bladder cancer | 2/13 | 0.001582883 | CXCL8/CDKN1A |
| hsa04621 | NOD-like receptor signaling pathway | 3/13 | 0.002344158 | NAMPT/CXCL8/NFKBIA |
| hsa04062 | Chemokine signaling pathway | 3/13 | 0.002488483 | CXCL8/NFKBIA/CXCR4 |
| hsa05167 | Kaposi sarcoma-associated herpesvirus infection | 3/13 | 0.002600297 | CXCL8/CDKN1A/NFKBIA |
| hsa05134 | Legionellosis | 2/13 | 0.002936004 | CXCL8/NFKBIA |
| hsa05417 | Lipid and atherosclerosis | 3/13 | 0.003426251 | SOD2/CXCL8/NFKBIA |
| hsa05166 | Human T-cell leukemia virus 1 infection | 3/13 | 0.003749438 | CDKN1A/ETS2/NFKBIA |
|  |  |  |  |  |
| Cluster4 |  |  |  |  |
| **ID** | **Description** | **GeneRatio** | **pvalue** | **geneID** |
| hsa04820 | Cytoskeleton in muscle cells | 7/42 | 9.61179E-05 | SDC2/COL6A3/SGCD/NID2/FHL1/TPM2/FBN1 |
| hsa00920 | Sulfur metabolism | 2/42 | 0.000966763 | PAPSS1/SELENBP1 |
| hsa00980 | Metabolism of xenobiotics by cytochrome P450 | 3/42 | 0.006127275 | CYP1B1/HSD11B1/AKR7A2 |
| hsa04610 | Complement and coagulation cascades | 3/42 | 0.008255652 | C7/F2R/F2RL2 |
| hsa04974 | Protein digestion and absorption | 3/42 | 0.013340564 | COL6A3/SLC1A5/COL15A1 |
| hsa04611 | Platelet activation | 3/42 | 0.021631712 | F2R/LCP2/PTGS1 |
| hsa05144 | Malaria | 2/42 | 0.023346222 | SDC2/CCL2 |
| hsa05135 | Yersinia infection | 3/42 | 0.02738969 | PYCARD/LCP2/CCL2 |
| hsa00140 | Steroid hormone biosynthesis | 2/42 | 0.034776817 | CYP1B1/HSD11B1 |
| hsa04261 | Adrenergic signaling in cardiomyocytes | 3/42 | 0.036221879 | CREB3L1/TPM2/ATP2B4 |
|  |  |  |  |  |
| Cluster5 |  |  |  |  |
| **ID** | **Description** | **GeneRatio** | **pvalue** | **geneID** |
| hsa04820 | Cytoskeleton in muscle cells | 14/82 | 2.22385E-08 | FN1/THBS2/COL5A1/COL6A3/SSPN/COL1A2/VCAN/ITGA2/FBN1/COL5A2/ITGA5/THBS1/ENO2/HSPG2 |
| hsa04512 | ECM-receptor interaction | 8/82 | 1.48251E-06 | FN1/THBS2/COL6A3/COL1A2/ITGA2/ITGA5/THBS1/HSPG2 |
| hsa04510 | Focal adhesion | 11/82 | 2.38786E-06 | FN1/THBS2/FLNB/COL6A3/COL1A2/ITGA2/ITGA5/VEGFC/EMP1/THBS1/PDGFRA |
| hsa04974 | Protein digestion and absorption | 8/82 | 5.19331E-06 | COL7A1/COL12A1/COL5A1/COL6A3/COL1A2/COL5A2/COL8A1/COL15A1 |
| hsa04933 | AGE-RAGE signaling pathway in diabetic complications | 7/82 | 3.87132E-05 | FN1/SERPINE1/MMP2/TGFB1/FOXO1/COL1A2/VEGFC |
| hsa05165 | Human papillomavirus infection | 12/82 | 5.13121E-05 | FN1/THBS2/COL6A3/FOXO1/COL1A2/ITGA2/ITGA5/THBS1/TBPL2/TCF7L1/PPP2R3C/TCF7 |
| hsa05412 | Arrhythmogenic right ventricular cardiomyopathy | 6/82 | 0.00013733 | SSPN/GJA1/ITGA2/ITGA5/TCF7L1/TCF7 |
| hsa05215 | Prostate cancer | 6/82 | 0.000281496 | FOXO1/MMP3/TCF7L1/TCF7/ZEB1/PDGFRA |
| hsa04350 | TGF-beta signaling pathway | 6/82 | 0.000475486 | LTBP1/TGFB1/FBN1/NBL1/THBS1/GREM1 |
| hsa05205 | Proteoglycans in cancer | 8/82 | 0.000575814 | FN1/MMP2/TGFB1/COL1A2/ITGA2/ITGA5/THBS1/HSPG2 |
|  |  |  |  |  |
| Cluster6 |  |  |  |  |
| **ID** | **Description** | **GeneRatio** | **pvalue** | **geneID** |
| hsa04950 | Maturity onset diabetes of the young | 10/167 | 1.79764E-11 | NEUROD1/FOXA2/NKX6-1/PAX6/NKX2-2/PDX1/RFX6/GCK/MAFA/MNX1 |
| hsa04911 | Insulin secretion | 12/167 | 6.29189E-08 | CAMK2B/KCNJ11/PDX1/RIMS2/ABCC8/STX1A/SNAP25/GCK/FXYD2/PCLO/ADCYAP1/CAMK2G |
| hsa04930 | Type II diabetes mellitus | 6/167 | 0.000234018 | KCNJ11/PDX1/ABCC8/GCK/MAFA/CACNA1A |
| hsa04530 | Tight junction | 11/167 | 0.000361612 | CLDN3/CLDN7/PARD6A/MYH14/LLGL2/PPP2R2B/SLC9A3R1/TUBA4A/EPB41L4B/TJP2/EZR |
| hsa04728 | Dopaminergic synapse | 9/167 | 0.000855982 | CAMK2B/GNG4/KIF5C/DDC/CALY/PPP2R2B/GNAO1/CACNA1A/CAMK2G |
| hsa04940 | Type I diabetes mellitus | 5/167 | 0.001217109 | GAD2/PTPRN/ICA1/PTPRN2/CPE |
| hsa04727 | GABAergic synapse | 7/167 | 0.001416748 | GNG4/GAD2/GABRB3/ABAT/GLS2/GNAO1/CACNA1A |
| hsa00650 | Butanoate metabolism | 4/167 | 0.00153188 | GAD2/ABAT/BDH1/HMGCLL1 |
| hsa00250 | Alanine, aspartate and glutamate metabolism | 4/167 | 0.004980401 | GAD2/NAT8L/ABAT/GLS2 |
| hsa05130 | Pathogenic Escherichia coli infection | 10/167 | 0.005047138 | CLDN3/PAK3/CLDN7/MYH14/MYO5B/SLC9A3R1/TUBA4A/CYFIP2/EZR/BAIAP2 |

**Table S10.JASPARA database predicts the top 10 transcription factors regulating LOXL2.**

| **JASPAR** | | | | | | | | |
| --- | --- | --- | --- | --- | --- | --- | --- | --- |
| **Matrix ID** | **Name** | **Score** | **Relative score** | **Sequence ID** | **Start** | **End** | **Strand** | **Predicted sequence** |
| [MA0024.2](https://jaspar.elixir.no/matrix/MA0024.2) | MA0024.2.E2F1 | 14.3382435 | 0.983736635 | NC_000008.11:c23406120-23404021 | 1790 | 1800 | + | CGGGCGCGAGG |
| [MA1511.2](https://jaspar.elixir.no/matrix/MA1511.2) | MA1511.2.KLF10 | 12.249779 | 0.933626659 | NC_000008.11:c23406120-23404021 | 1945 | 1953 | + | GGGGAGGGG |
| [MA1114.2](https://jaspar.elixir.no/matrix/MA1114.2) | MA1114.2.PBX3 | 12.142219 | 0.870249162 | NC_000008.11:c23406120-23404021 | 136 | 146 | + | TGAGTGAAAGC |
| [MA0470.1](https://jaspar.elixir.no/matrix/MA0470.1) | MA0470.1.E2F4 | 11.991917 | 0.934715037 | NC_000008.11:c23406120-23404021 | 1791 | 1801 | + | GGGCGCGAGGA |
| [MA1114.1](https://jaspar.elixir.no/matrix/MA1114.1) | MA1114.1.PBX3 | 11.6743355 | 0.83889864 | NC_000008.11:c23406120-23404021 | 133 | 149 | + | AGCTGAGTGAAAGCTCA |
| [MA0640.3](https://jaspar.elixir.no/matrix/MA0640.3) | MA0640.3.ELF3 | 9.987969 | 0.883629596 | NC_000008.11:c23406120-23404021 | 1096 | 1104 | - | CACTCCCTG |
| [MA1511.2](https://jaspar.elixir.no/matrix/MA1511.2) | MA1511.2.KLF10 | 9.950808 | 0.895457608 | NC_000008.11:c23406120-23404021 | 1934 | 1942 | + | GGGGTGGGT |
| [MA0640.2](https://jaspar.elixir.no/matrix/MA0640.2) | MA0640.2.ELF3 | 9.885749 | 0.846975687 | NC_000008.11:c23406120-23404021 | 1533 | 1546 | - | TCCCTTTTCCTGTA |
| [MA0640.2](https://jaspar.elixir.no/matrix/MA0640.2) | MA0640.2.ELF3 | 9.804783 | 0.845450967 | NC_000008.11:c23406120-23404021 | 1094 | 1107 | - | CTCCACTCCCTGAG |
| [MA0640.3](https://jaspar.elixir.no/matrix/MA0640.3) | MA0640.3.ELF3 | 9.755575 | 0.878742309 | NC_000008.11:c23406120-23404021 | 1381 | 1389 | - | TCCTTCCTG |

**Table S11.AnimalTFDB v4 database predicts the top 10 transcription factors regulating LOXL2.**

| **AnimalTFDB v4.0** | | | | | | | | |
| --- | --- | --- | --- | --- | --- | --- | --- | --- |
| **TF** | **Query** | **Start** | **Stop** | **Strand** | **Score** | **Pvalue** | **Qvalue** | **Mached Sequence** |
| KLF10 | NC_000008.11:c23406120-23404021 | 1933 | 1954 | - | 19.2449 | 6.39E-08 | 0.000229 | ACCCCTCCCCGGACCCACCCCC |
| KLF10 | NC_000008.11:c23406120-23404021 | 1929 | 1943 | - | 18.0714 | 3.20E-07 | 0.00111 | GACCCACCCCCTTCC |
| E2F1 | NC_000008.11:c23406120-23404021 | 1790 | 1800 | + | 15.9508 | 1.17E-06 | 0.00375 | CGGGCGCGAGG |
| E2F1 | NC_000008.11:c23406120-23404021 | 1790 | 1800 | + | 15.9508 | 1.17E-06 | 0.00375 | CGGGCGCGAGG |
| KLF10 | NC_000008.11:c23406120-23404021 | 1933 | 1943 | - | 14.4286 | 8.15E-06 | 0.0306 | GACCCACCCCC |
| KLF10 | NC_000008.11:c23406120-23404021 | 1892 | 1906 | - | 14.4082 | 6.44E-06 | 0.0111 | GCCCGGCCTCCGGCC |
| OSR2 | NC_000008.11:c23406120-23404021 | 455 | 470 | - | 13.7982 | 8.66E-06 | 0.0326 | GTCCCAGCTACTCGGG |
| E2F1 | NC_000008.11:c23406120-23404021 | 1786 | 1800 | + | 13.6327 | 1.26E-05 | 0.0355 | AACCCGGGCGCGAGG |
| E2F1 | NC_000008.11:c23406120-23404021 | 1786 | 1800 | + | 13.6327 | 1.26E-05 | 0.0355 | AACCCGGGCGCGAGG |
| KLF10 | NC_000008.11:c23406120-23404021 | 1945 | 1953 | + | 13.3708 | 1.99E-05 | 0.0699 | GGGGAGGGG |
