# Supplementary material for: MMA-induced LOXL2+ PSCs promote linear ECM alignment in the aging pancreas leading to pancreatic cancer progression
Source: Cell Death Dis. 2025 May 27;16(1):419. doi: 10.1038/s41419-025-07751-5 (PMC12116754; doi:10.1038/s41419-025-07751-5)
Supplement: Supplementary file 2 — Supplementary Figures S1–S13 [file 41419_2025_7751_MOESM2_ESM.pdf]

A

		Univariate analysis			Multivariate analysis		
		HR	HR(95%CI)	pvalue	HR	HR(95%CI)	pvalue
Age							
	Young	1.00					
	Aged	1.57	1.51-1.63	<0.001	1.52	1.47-1.58	<0.001
Sex							
	Male	1.00					
	Female	0.95	0.92-0.99	0.01	0.95	0.91-0.98	0.01
Race							
	White	1.00					
	Black	1.05	0.99-1.11	0.13	1.14	1.08-1.21	<0.001
	Others	0.87	0.81-0.93	<0.001	0.93	0.87-1.00	0.04
Location							
	Head	1.00					
	Body	0.85	0.81-0.90	<0.001	0.93	0.87-0.98	0.01
	Tail	0.59	0.56-0.62	<0.001	0.70	0.66-0.74	<0.001
Grade							
	G1	1.00					
	G2	2.65	2.50-2.81	<0.001	2.10	2-2.2	<0.001
	G3	4.22	3.98-4.48	<0.001	3.00	2.9-3.2	<0.001
	G4	3.91	3.43-4.45	<0.001	2.60	2.3-3	<0.001
Stage_T							
	T1	1.00					
	T2	3.19	2.89-3.52	<0.001	2.10	1.9-2.3	<0.001
	T3	3.93	3.58-4.31	<0.001	2.10	1.9-2.3	<0.001
	T4	6.94	6.28-7.68	<0.001	3.20	2.9-3.5	<0.001
Stage_N							
	N0	1.00					
	N1	1.36	1.31-1.41	<0.001	1.10	1-1.1	<0.001
Stage_M							
	M0	1.00					
	M1	2.65	2.54-2.77	<0.001	2.50	2.4-2.6	<0.001

B

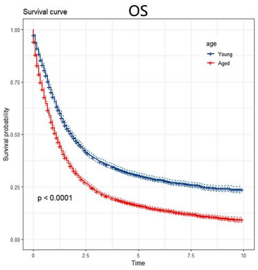

D

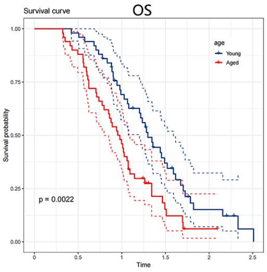

C

	T12	T34	
Aged	1576	4856	
Young	1386	3201	
			P < 0.0001
	G12	G34	
Aged	4120	2312	
Young	3221	1366	
			P < 0.0001
	N0	N1	
Aged	3146	3286	
Young	2188	2399	
			P=0.21

E

	T12	T34		
Young	31	19		
Aged	17	33		
			P=0.005	
	LVI	Non-LVI		
Young	17	33		
Aged	33	17		
			P=0.001	
	PNI	Non-PNI		
Young	25	25		
Aged	40	10		
			P=0.002	
	N0	N1		
Young	12	38		
Aged	19	31		
			P=0.13	
	G1	G2	G3	
Young	16	28	6	
Aged	5	28	17	
				P=0.004

Figure S1:The Aged is an independent adverse prognostic factor in pancreatic cancer patients. (A) Univariate and multivariate Cox analyses of aged and young PDAC patients in the SEER database. (B) OS of aged and young PDAC patients in the SEER database. (C) Comparison of pathological features between aged and young PDAC patients in the SEER database via the chi-square test. (D) OS of aged and young PDAC patients at Zhongda Hospital. (E) Comparison of pathological features between aged and young PDAC patients at Zhongda Hospital via the chi-square test.

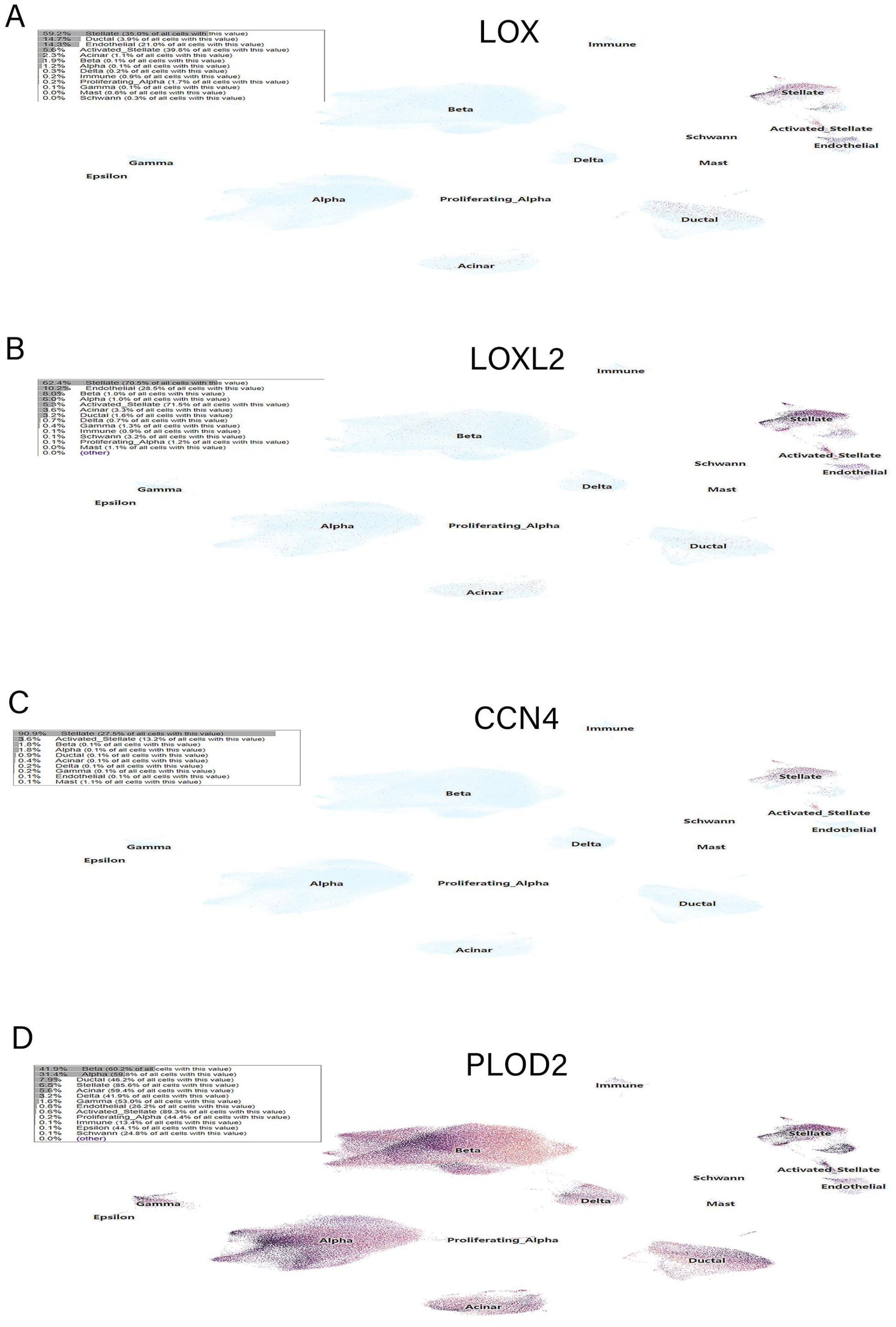

Figure S2: The UCSC Cell Browser scRNA-seq dataset showing the expression of linear ECM alignment-related genes in various normal pancreatic cell lines.(A).LOX. (B).LOXL2. (C).CCN4. (D).PLOD2.

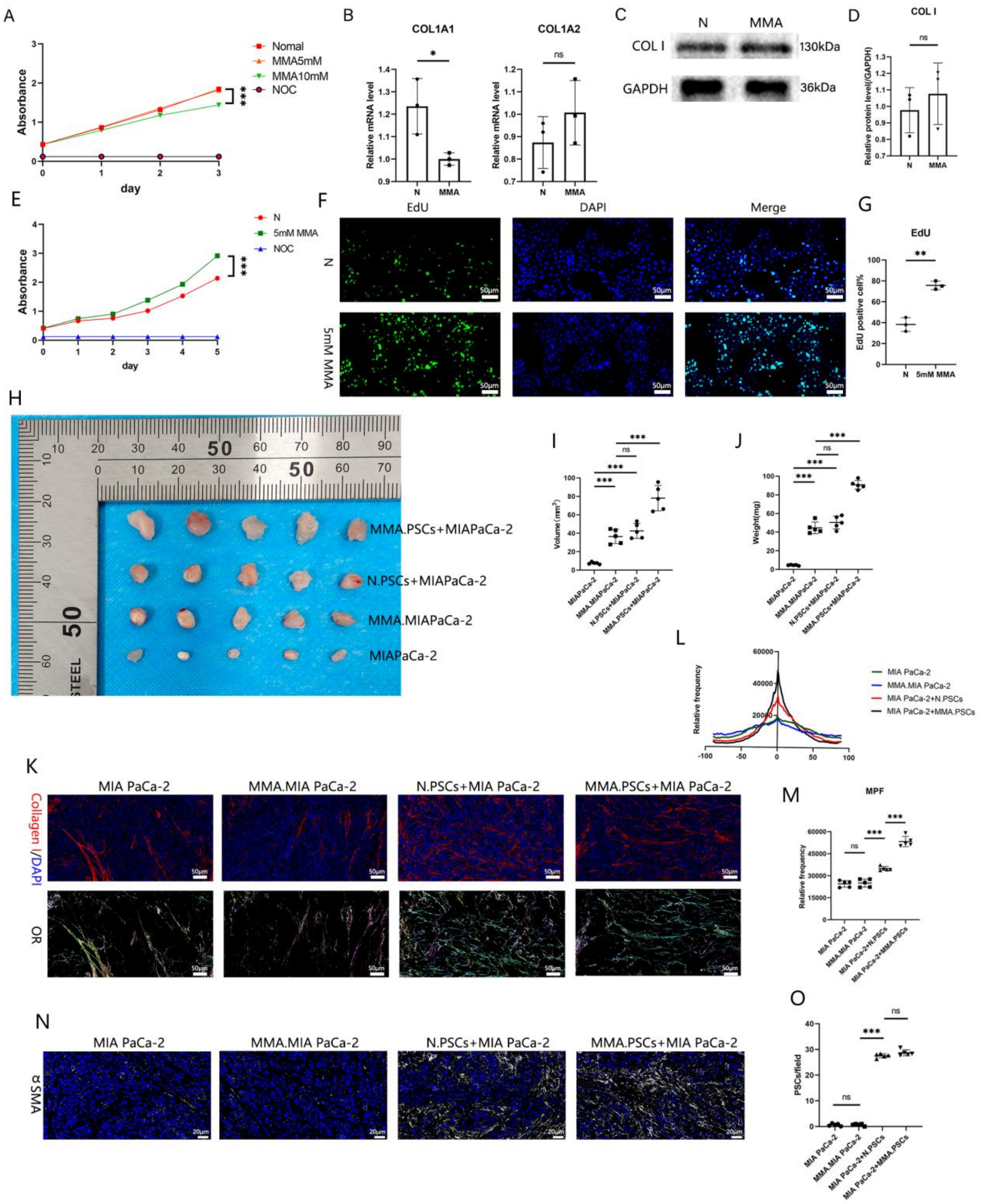

Figure S3:MMA-induced PSCs to increase linear ECM alignment. (A) A CCK-8 assay was performed to evaluate the viability of HPSCs treated with MMA at different concentrations and for different durations (n=5 biological replicates). (B) qPCR analysis of COL1A1 and COL1A2 expression in the N.HPSCs and MMA.HPSCs (n=3 biological replicates). Representative (C) and quantitative (D) WB results of COL I expression in the N.HPSCs and MMA.HPSCs (n=3 biological

replicates) .(E) A CCK-8 assay was performed to evaluate the proliferative capacity of MIA PaCa-2 treated with 5mM MMA.Treatment with DEPC water was used as a control. (n=3 biological replicates).(F) EdU assay was performed to evaluate the proliferative capacity of MIA PaCa-2 cells treated with 5 mM MMA. Treatment with DEPC water was used as a control.(n = 3 biological replicates).(G) Number of EdU positive cells in (F). (H-J) Comparison of tumor volume and weight between MIA PaCa-2 tumors,MMA+MIA PaCa-2 tumors,MMA.HPSC+MIA PaCa-2 tumors and N.HPSC+MIA PaCa-2 tumors (n=5 mice per group). (K-M) Comparison of linear ECM alignment between MIA PaCa-2 tumors,MMA+MIA PaCa-2 tumors,MMA.HPSC+MIA PaCa-2 tumors and N.HPSC+MIA PaCa-2 tumors. Linear ECM alignment in tumor tissues was assessed via IF staining for COL I (n=5 mice per group).(N) IF staining of αSMA in the MIA PaCa-2 tumors,MMA+MIA PaCa-2 tumors,MMA.HPSC+MIA PaCa-2 tumors and N.HPSC+MIA PaCa-2 tumors. αSMA (white) (n=5 mice per group). (O) Number of αSMA stained cells (PSCs) in (N).

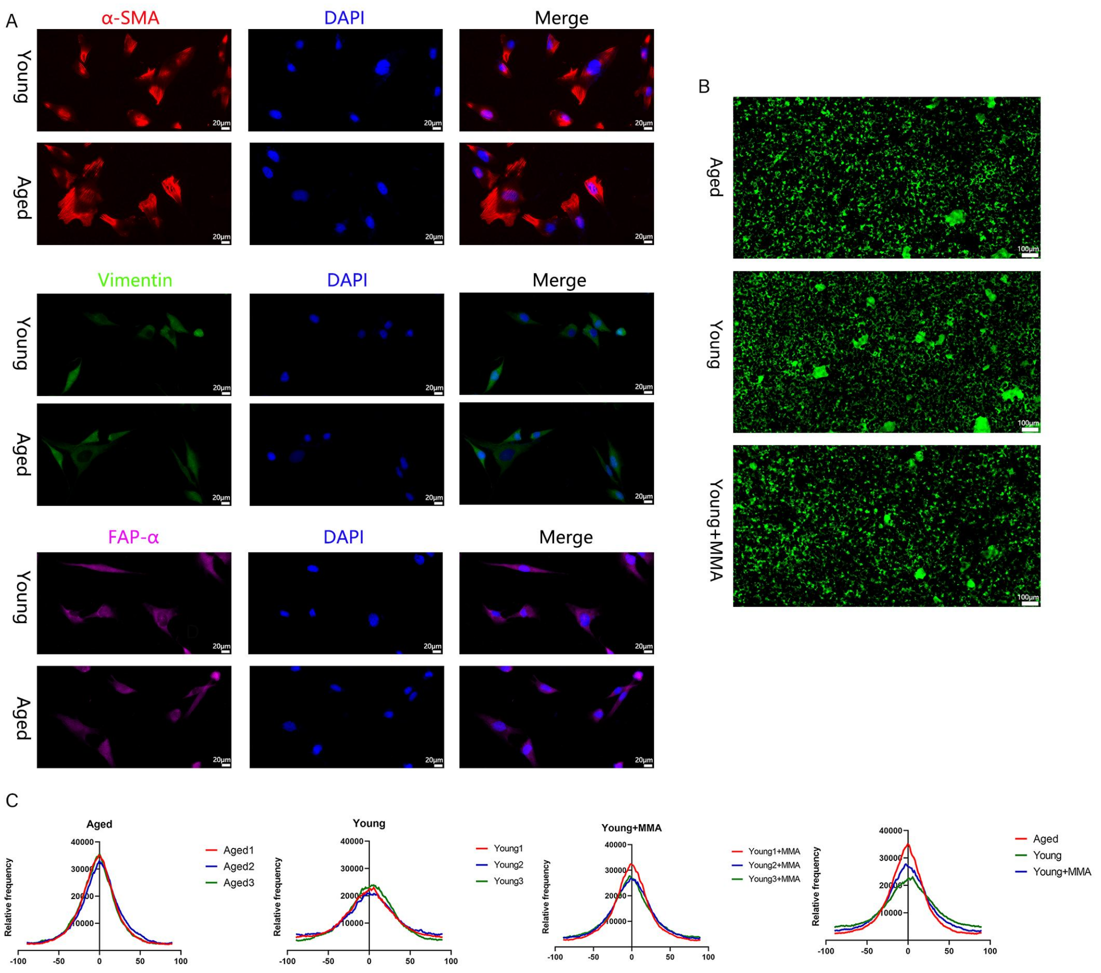

Figure S4:MMA induces LOXL2 expression in Aged.PSCs to increase linear ECM alignment. (A) IF staining of αSMA,Vimentin,FAP-α in the Aged.PSC and Young.PSC groups. (B) ECM production in the Aged.PSC, Young.PSC, and MMA.Young.PSC groups (n=3 biological replicates). (C) Comparison of linear ECM alignment as measured in (B). (*p<0.05, **p<0.01, ***p<0.001)

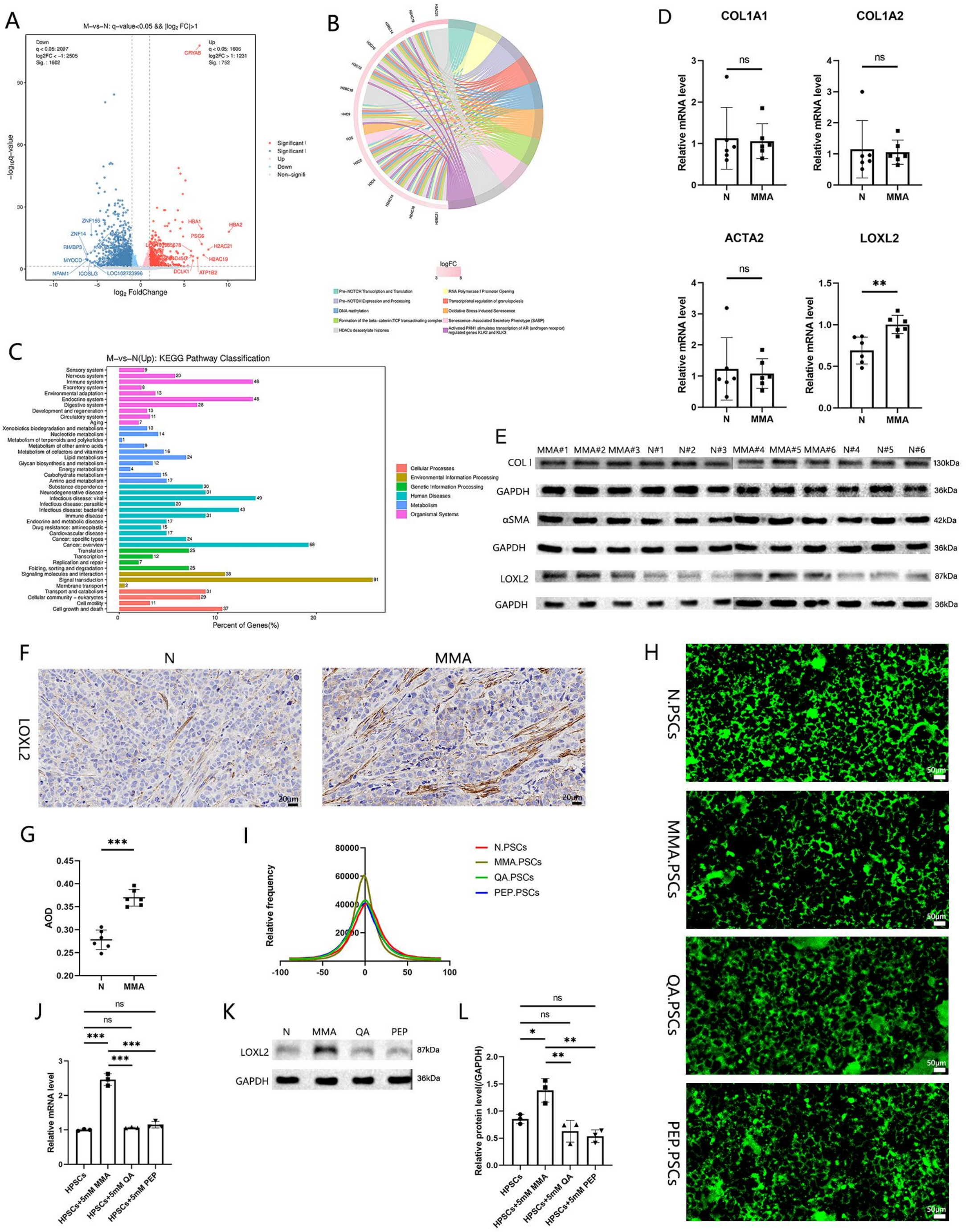

Figure S5:MMA induces LOXL2 expression in PSCs to increase linear ECM alignment. (A) Volcano plot showing the DEGs between MMA.HPSCs and control HPSCs. (B) Reactome analyses based on the genes whose expression was upregulated in MMA.HPSCs. (C) KEGG analysis based on the genes whose expression was upregulated in MMA.HPSCs.(D) qPCR analysis of COL1A1,COL1A2,ACTA2 and

LOXL2 expression in the MMA.HPSC+MIA PaCa-2 tumors and N.HPSC+MIA PaCa-2 tumors (n=6 mice per group). (E) WB results of COL I, α SMA,LOXL2 expression in the MMA.HPSC+MIA PaCa-2 tumors and N.HPSC+MIA PaCa-2 tumors (n=6 mice per group). (F-G) IHC analysis of LOXL2 expression in the MMA.HPSC+MIA PaCa-2 tumors and N.HPSC+MIA PaCa-2 tumors (n=6 mice per group). (H) ECM production in HPSCs treated with with 5 mM MMA, 5 mM QA, and 5 mM PEP,separately (n=3 biological replicates). (I) Comparison of linear ECM alignment as measured in (H). (J) qPCR analysis of LOXL2 expression in HPSCs treated with with 5 mM MMA, 5 mM QA, and 5 mM PEP,separately (n=3 biological replicates). Representative (K) and quantitative (L) WB results of LOXL2 expression in HPSCs treated with with 5 mM MMA, 5 mM QA, and 5 mM PEP,separately (n=3 biological replicates).(*$p<0.05$, **$p<0.01$, ***$p<0.001$)

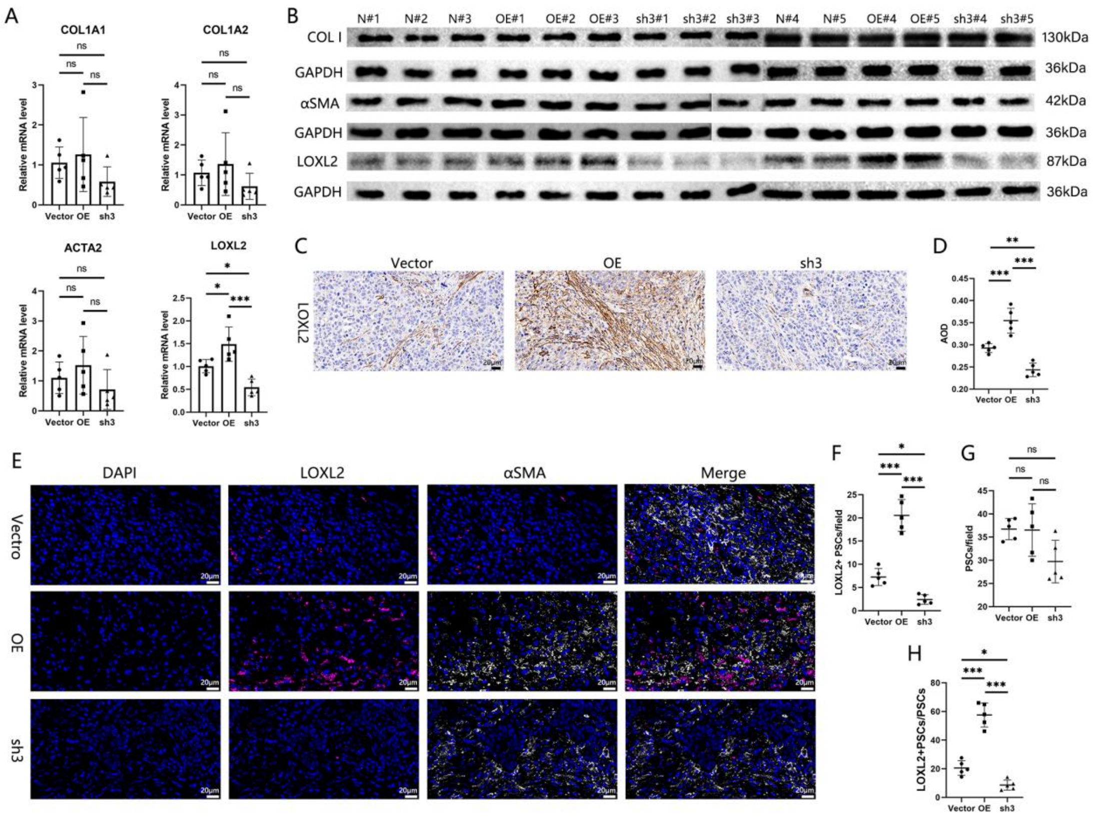

Figure S6: LOXL2 in PSCs increases linear ECM alignment and accelerates PDAC progression. (A) qPCR analysis of COL1A1,COL1A2,ACTA2 and LOXL2 expression in the Vector.HPSC+MIA PaCa-2 tumors,OE-LOXL2.HPSC+MIA PaCa-2 tumors and sh-LOXL2.HPSC+MIA PaCa-2 tumors (n=5 mice per group). (B) WB results of COL I, αSMA,LOXL2 expression in the Vector-LOXL2.HPSC+MIA PaCa-2 tumors,OE-LOXL2.HPSC+MIA PaCa-2 tumors and sh-LOXL2.HPSC+MIA PaCa-2 tumors (n=5 mice per group). (C-D) IHC analysis of LOXL2 expression in the Vector-LOXL2.HPSC+MIA PaCa-2 tumors,OE-LOXL2.HPSC+MIA PaCa-2 tumors and sh-LOXL2.HPSC+MIA PaCa-2 tumors (n=5 mice per group). (E) Multiplex IF staining of the Vector-LOXL2.HPSC+MIA PaCa-2 tumors,OE-LOXL2.HPSC+MIA PaCa-2 tumors and sh-LOXL2.HPSC+MIA PaCa-2 tumors. αSMA (white), LOXL2 (pink) (n=5 mice per group). (F) Number of

double-stained cells ($LOXL2^{+}$PSCs) in (E). (G) Number of αSMA stained cells (PSCs) in (E). (H) The proportion of $LOXL2^{+}$ PSCs among the total PSCs in (E).(*$p<0.05$, **$p<0.01$, ***$p<0.001$)

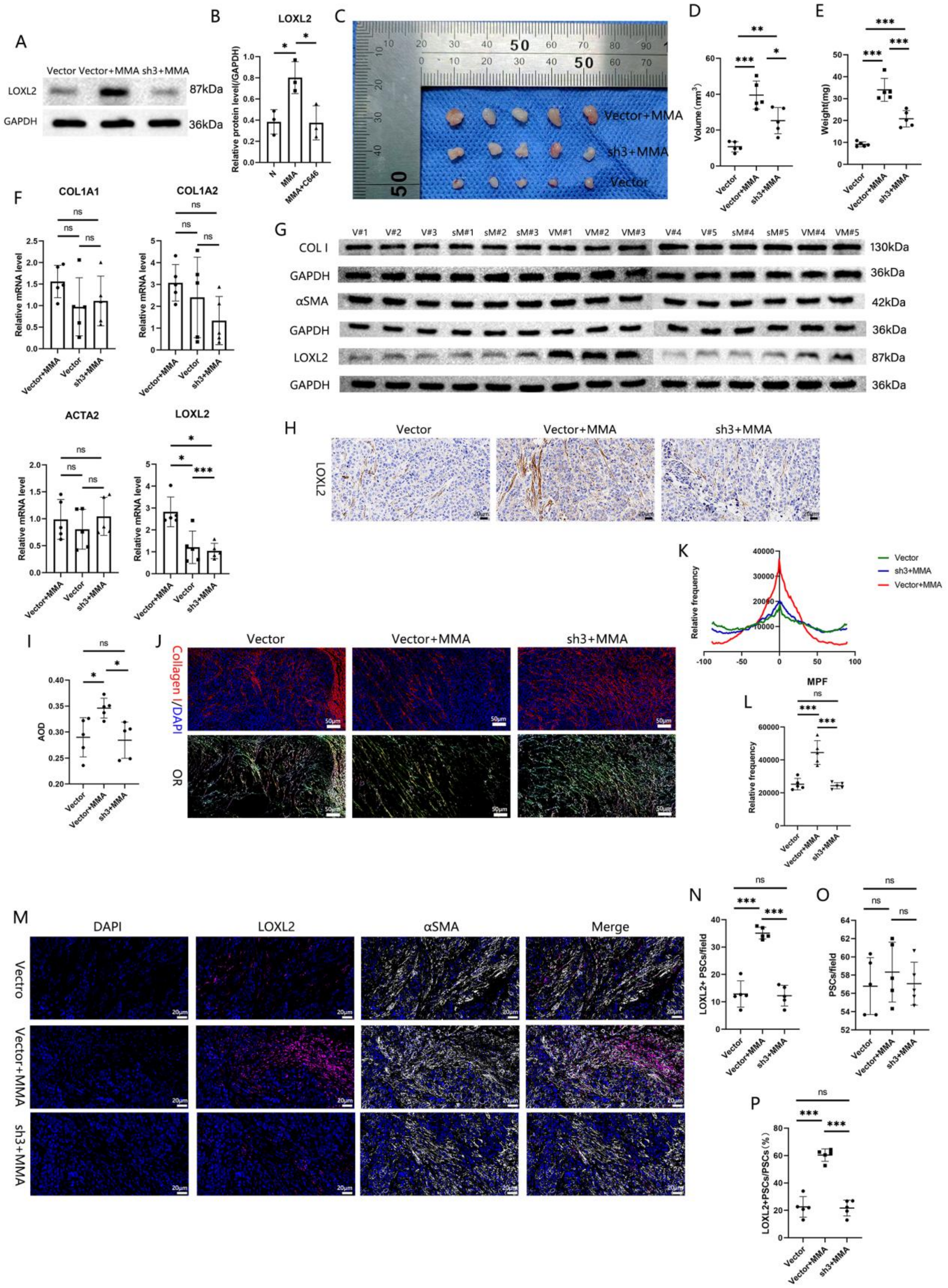

Figure S7:LOXL2 deficiency in PSCs inhibits MMA-induced linear ECM alignment and PDAC progression. Representative (A) and quantitative (B) WB results of LOXL2 expression in the Vector-LOXL2.HPSC,

Vector-LOXL2.HPSC+MMA, sh3-LOXL2.HPSCs+MMA (n=3 biological replicates).(C-E) Comparison of tumor volume and weight between the Vector-LOXL2.HPSC+MIA PaCa-2 tumors, MMA.Vector-LOXL2.HPSC+MIA PaCa-2 tumors and MMA.sh3-LOXL2.HPSC+MIA PaCa-2 tumors (n=5 mice per group). MMA.Vector-LOXL2.HPSC+MIA PaCa-2 tumors: Vector-LOXL2.HPSC cells were induced with 5 mM MMA for three days and then co-injected with MIA PaCa-2 cells to form tumors.MMA.sh3-LOXL2.HPSC+MIA PaCa-2 tumors: sh3-LOXL2.HPSC cells were induced with 5 mM MMA for three days and then co-injected with MIA PaCa-2 cells to form tumors. (F) qPCR analysis of COL1A1,COL1A2,ACTA2 and LOXL2 expression in the tumors in (C) (n=5 mice per group). (G) WB results of COL I,αSMA,LOXL2 expression in the tumors in (C) (n=5 mice per group). sM:MMA.sh3-LOXL2.HPSC+MIA PaCa-2 tumors,VM:MMA.Vector-LOXL2.HPSC+MIA PaCa-2 tumors. (H-I) IHC analysis of LOXL2 expression in the tumors in (C) (n=5 mice per group). (J-L) Comparison of linear ECM alignment between the tumors in (C). Linear ECM alignment in tumor tissues was assessed via IF staining for COL I (n=5 mice per group). (M) Multiplex IF staining of the tumors in (C). αSMA (white), LOXL2 (pink) (n=5 mice per group). (N) Number of double-stained cells ($LOXL2^{+}$PSCs) in (M). (O) Number of αSMA stained cells (PSCs) in (M). (P) The proportion of $LOXL2^{+}$ PSCs among the total PSCs in (M).(*p<0.05, **p<0.01, ***p<0.001)

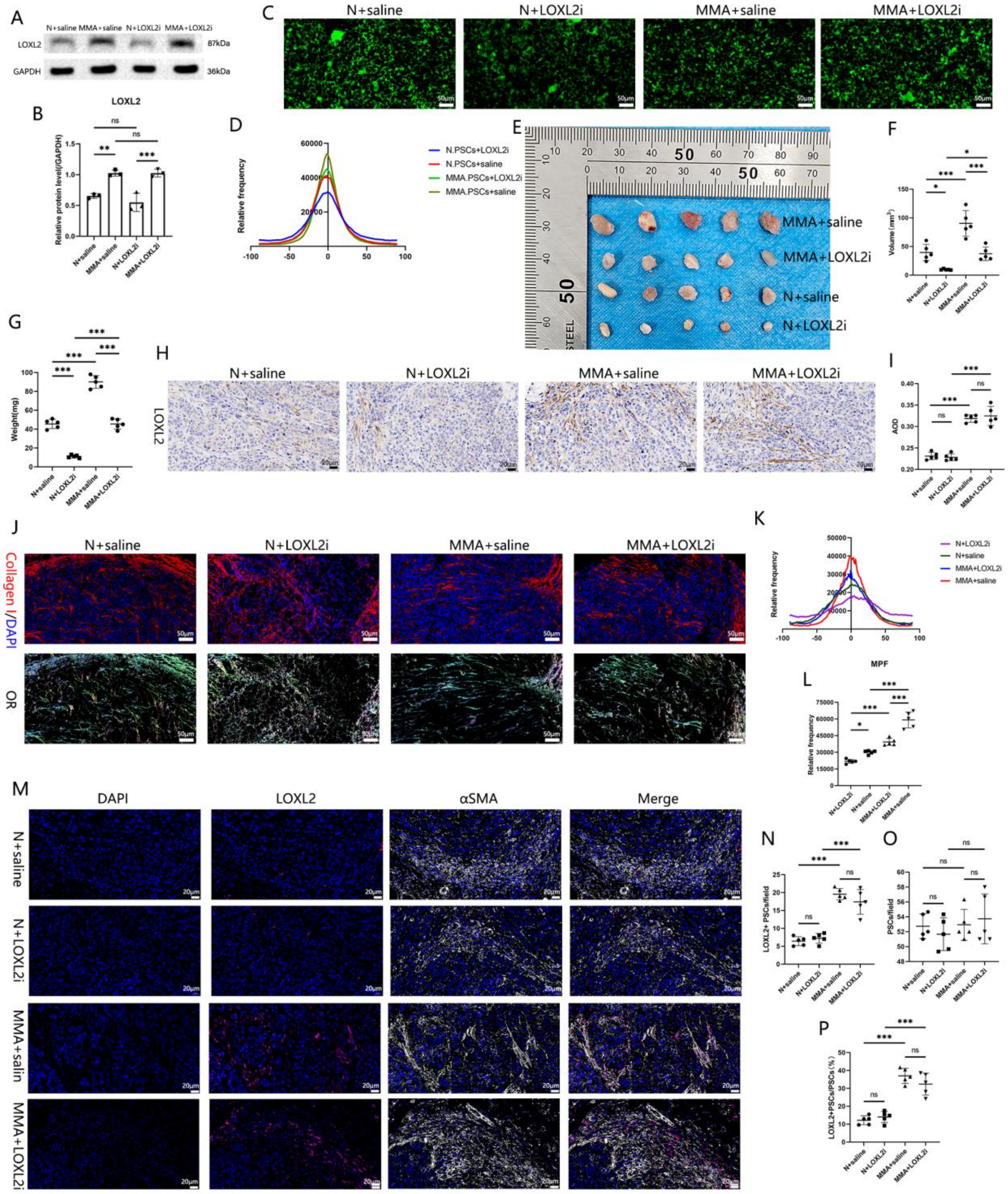

Figure S8:LOXL2i inhibits MMA-induced linear ECM alignment and PDAC progression. Representative (A) and quantitative (B) WB results of LOXL2 expression in the N.HPSC+saline,N.HPSC+LOXL2i, MMA.HPSCs+saline, MMA.HPSCs+LOXL2i (n=3 biological replicates). (C) ECM production in the N.HPSC+saline,N.HPSC+LOXL2i, MMA.HPSCs+saline, MMA.HPSCs+LOXL2i (n=3 biological replicates). (D) Comparison of linear ECM alignment as measured in

(C). (E-G) Comparison of tumor volume and weight between the N.HPSC+MIA PaCa-2+saline tumors, N.HPSC+MIA PaCa-2+LOXL2i tumors, MMA.HPSC+MIA PaCa-2+saline tumors and MMA.HPSC+MIA PaCa-2+LOXL2i tumors (n=5 mice per group). (H-I) IHC analysis of LOXL2 expression in tumors in (E) (n=5 mice per group). (J-L) Comparison of linear ECM alignment between the tumors in (E). Linear ECM alignment in tumor tissues was assessed via IF staining for COL I (n=5 mice per group). (M) Multiplex IF staining of the tumors in (E). αSMA (white), LOXL2 (pink) (n=5 mice per group). (N) Number of double-stained cells ($LOXL2^{+}$PSCs) in (M). (O) Number of αSMA stained cells (PSCs) in (M). (P) The proportion of $LOXL2^{+}$ PSCs among the total PSCs in (M).(*$p<0.05$, **$p<0.01$, ***$p<0.001$)

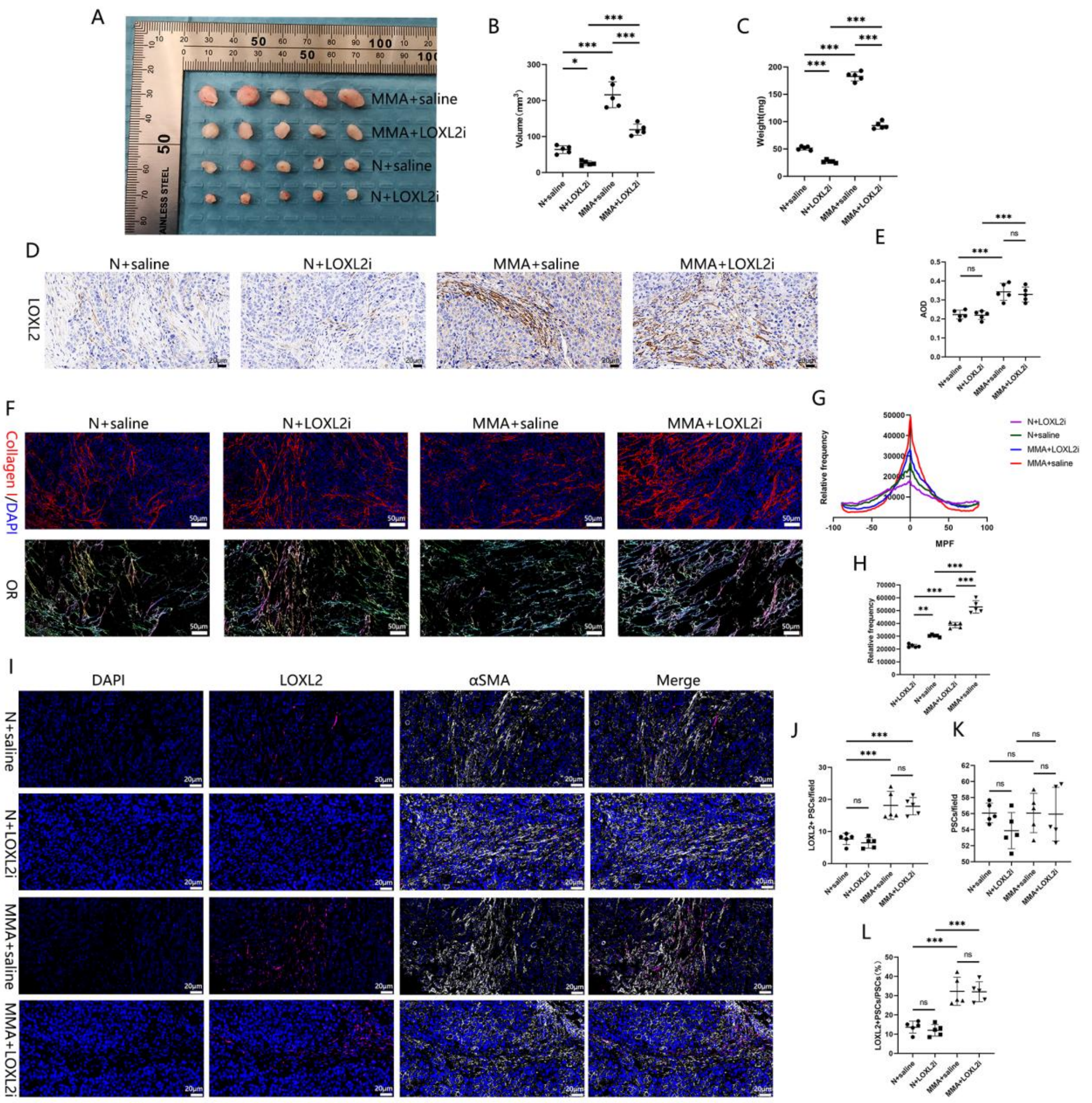

Figure S9:LOXL2i inhibits MMA-induced linear ECM alignment and PDAC progression in vivo. (A-C) Comparison of tumor volume and weight between the N.HPSC+Capan2+saline tumors, N.HPSC+Capan2+LOXL2i tumors, MMA.HPSC+Capan2+saline tumors and MMA.HPSC+Capan2+LOXL2i tumors (n=5 mice per group). (D-E) IHC analysis of LOXL2 expression in the tumors in (A) (n=5 mice per group). (F-H) Comparison of linear ECM alignment between the tumors in (A). Linear ECM alignment in tumor tissues was assessed via IF staining for COL I (n=5 mice per group). (I) Multiplex IF staining of the tumors in (A). α SMA (white), LOXL2 (pink) (n=5 mice per group). (J) Number of double-stained

cells ($LOXL2^+$PSCs) in (I). (K) Number of αSMA stained cells (PSCs) in (I). (L) The proportion of $LOXL2^+$ PSCs among the total PSCs in (I).(*$p<0.05$, **$p<0.01$, ***$p<0.001$)

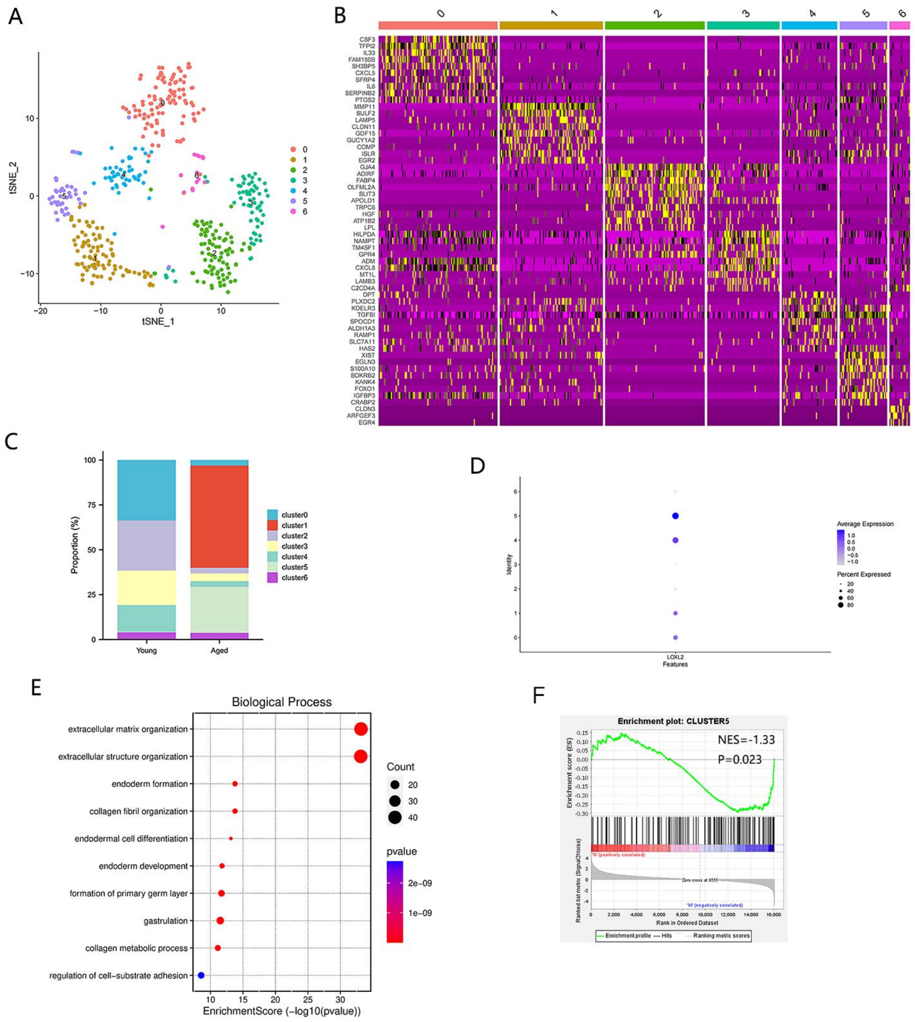

Figure S10：LOXL2⁺C5.PSCs in pancreas. (A) Reclustering analysis of PSCs. (B) Heatmap showing the expression of signature genes in each PSC subcluster. (C) The proportion of each PSCs cluster in the aging and young pancreas. (D) Bubble plot displaying LOXL2 expression in each PSC subcluster. (E) GO analysis of signature genes in C5.PSCs. (F) GSEA of MMA.HPSCs, control HPSCs and C5.PSCs.

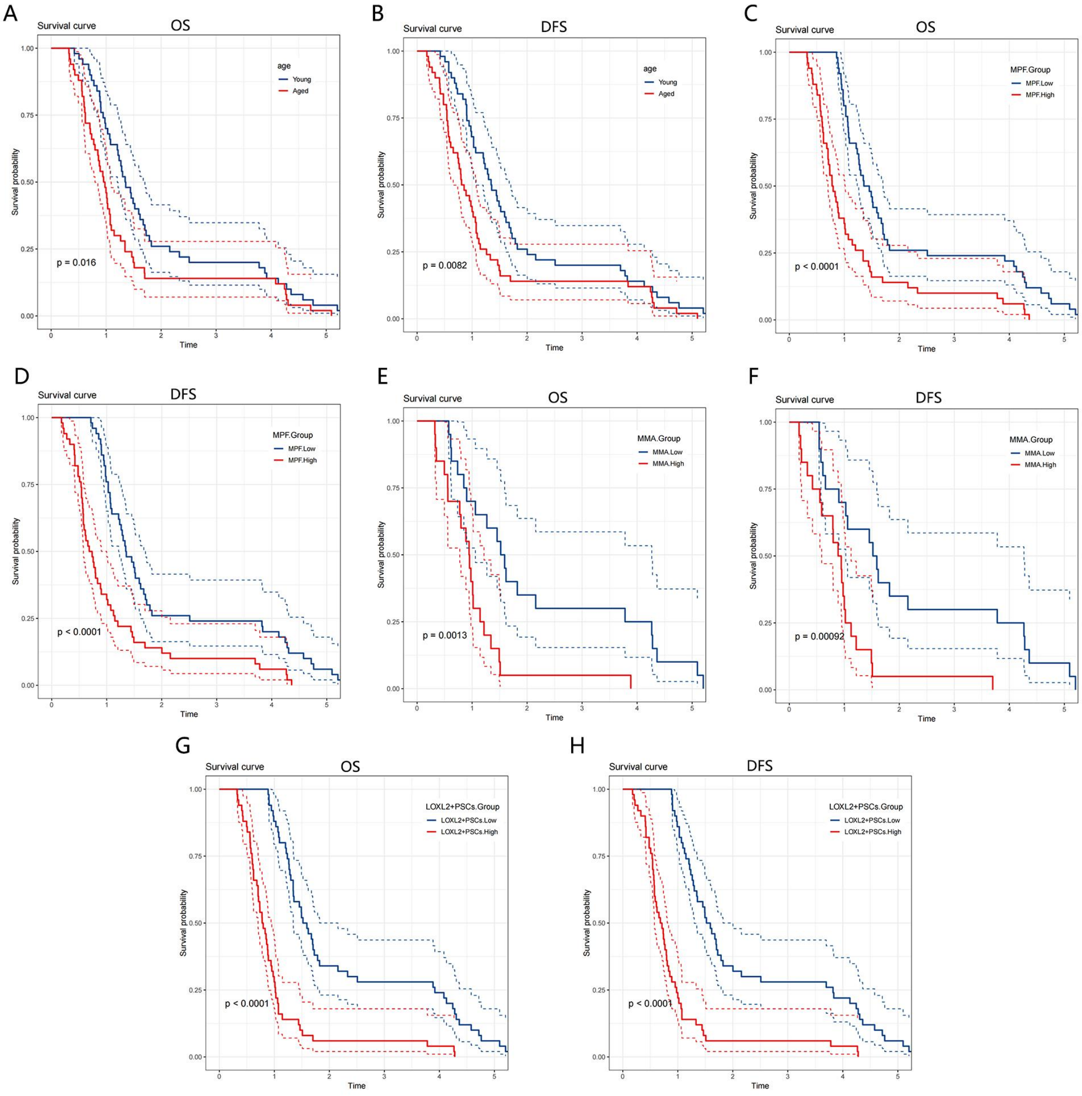

Figure S11：Long-term follow-up of a separate cohort of 100 PDAC Patients from Zhongda Hospital. (A) OS of aged and young PDAC patients at Zhongda Hospital. (B) DFS of aged and young PDAC patients at Zhongda Hospital. (C) OS of the MPF.High and MPF.Low groups. (D) DFS of the MPF.High and MPF.Low groups. (E) OS of the MMA.High and MMA.Low groups. (F) DFS of the MMA.High and MMA.Low groups. (G) OS of the LOXL2^{+}PSCs.High and LOXL2^{+}PSCs.Low groups. (H) DFS of the LOXL2^{+}PSCs.High and LOXL2^{+}PSCs.Low groups.

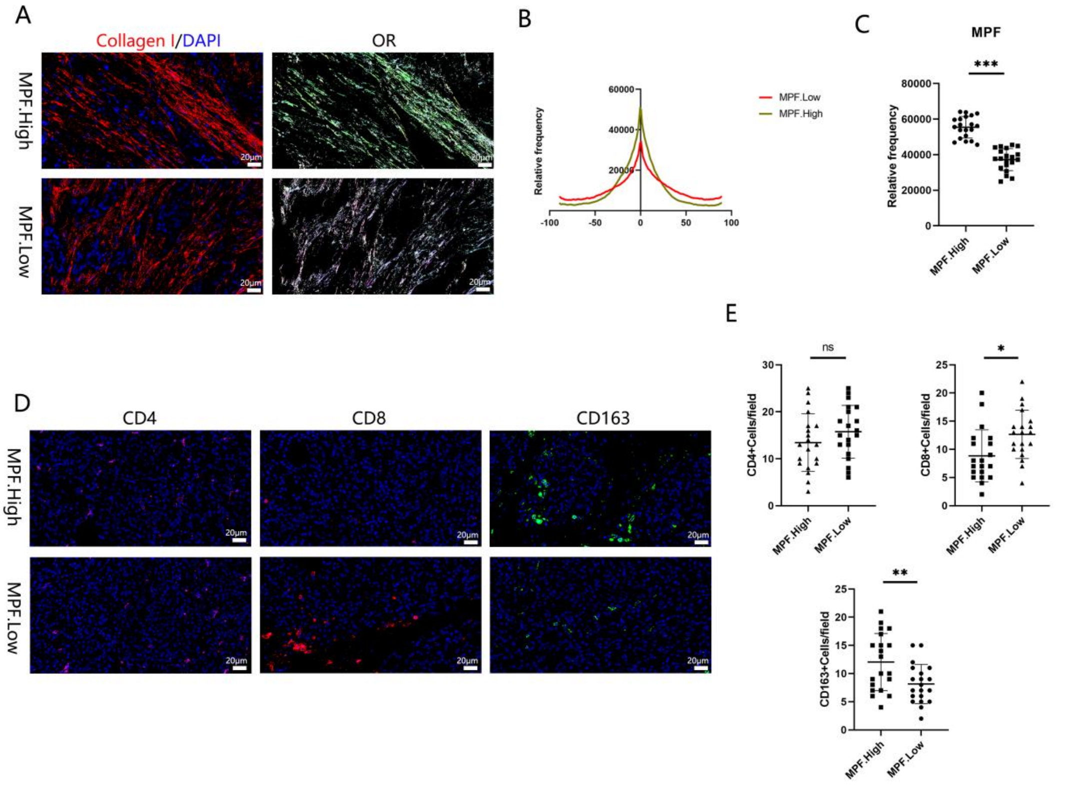

Figure S12:Enhanced linear ECM alignment in tumor tissues is associated with an immunosuppressive tumor microenvironment. (A-C) Comparison of linear ECM alignment between the tumors in MPF.High group and MPF.Low group. Linear ECM alignment in tumor tissues was assessed via IF staining for COL I. (D) IF staining of the tumors in MPF.High group and MPF.Low group. CD4 (pink), CD8 (red), CD163 (green) . (E) Number of $CD4^+$ T cells, $CD8^+$ T cells, and $CD163^+$ M2 macrophages in (D).(*p<0.05, **p<0.01, ***p<0.001)

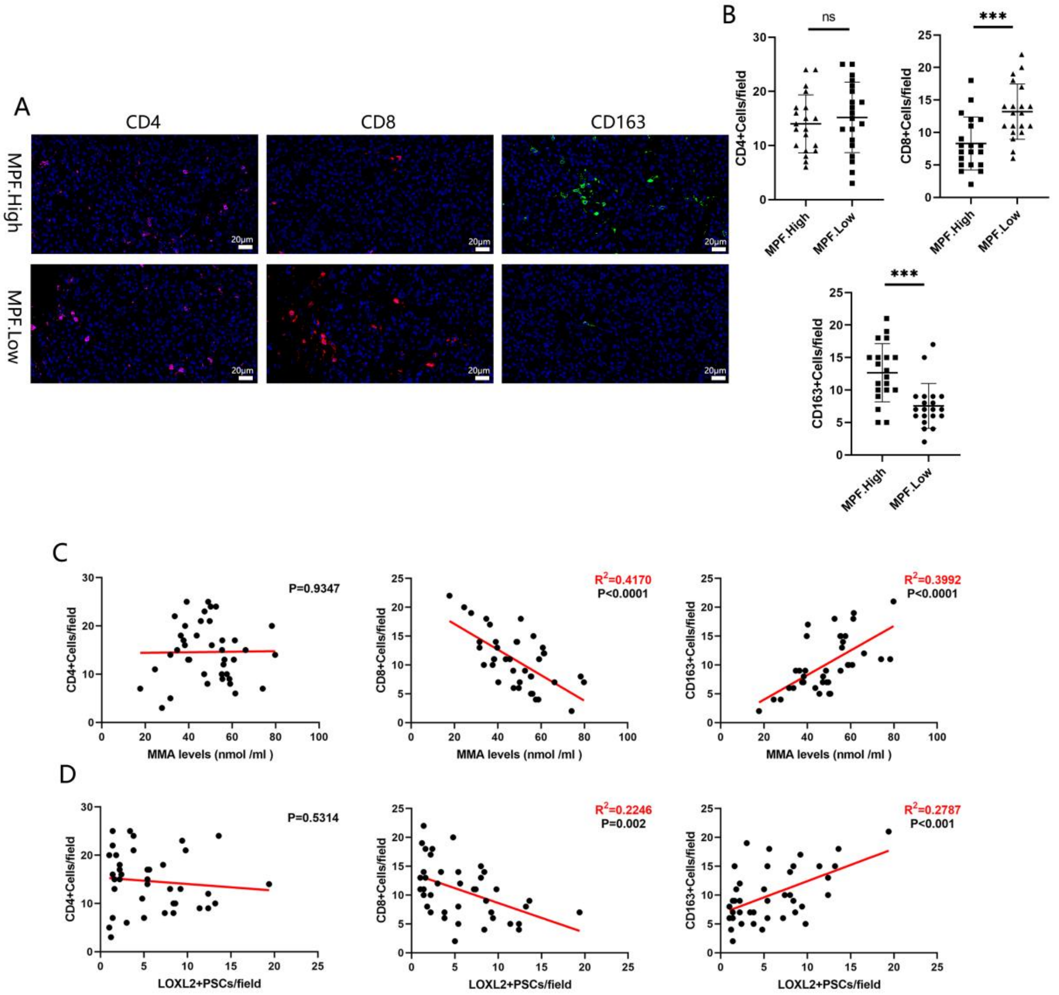

Figure S13:The MMA–LOXL2$^+$ PSC–ECM axis in adjacent **normal pancreatic tissues is associated with an immunosuppressive tumor microenvironment.**(A) IF staining of tumors stratified by linear ECM alignment in adjacent normal pancreatic tissues (MPF.High group vs MPF.Low group). CD4 (pink), CD8 (red), CD163 (green) . (B) Number of CD4$^+$ T cells, CD8$^+$ T cells, and CD163$^+$ M2 macrophages in (A). (C) Correlation analysis between serum MMA levels and immune cells infiltration in tumor tissues.(D) Correlation analysis between LOXL2$^+$PSCs infiltration in adjacent normal pancreatic tissues and immune cells infiltration in tumor tissues.(*p<0.05, **p<0.01, ***p<0.001)
