# Supplementary material for: MMA-induced LOXL2+ PSCs promote linear ECM alignment in the aging pancreas leading to pancreatic cancer progression
Source: Cell Death Dis. 2025 May 27;16(1):419. doi: 10.1038/s41419-025-07751-5 (PMC12116754; doi:10.1038/s41419-025-07751-5)

**Figure 4B**

GAPDH


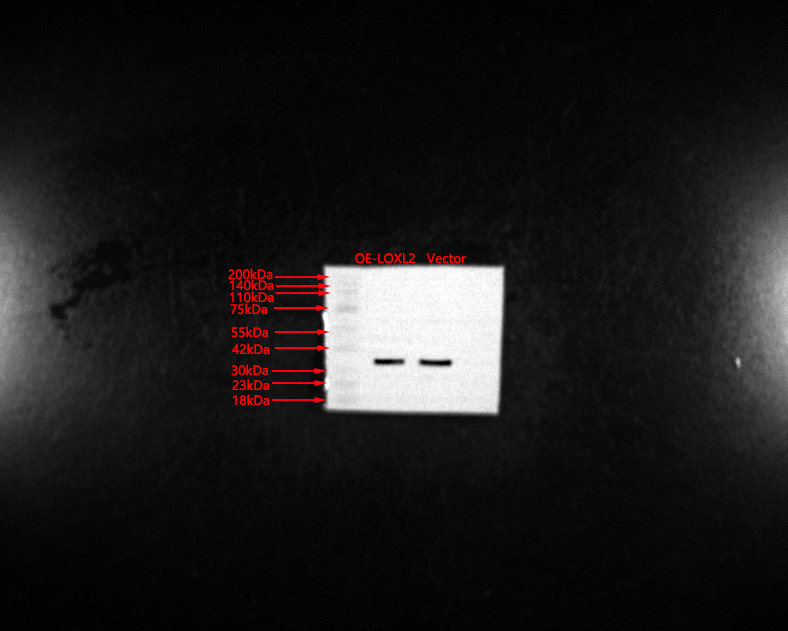


OE-LOXL2


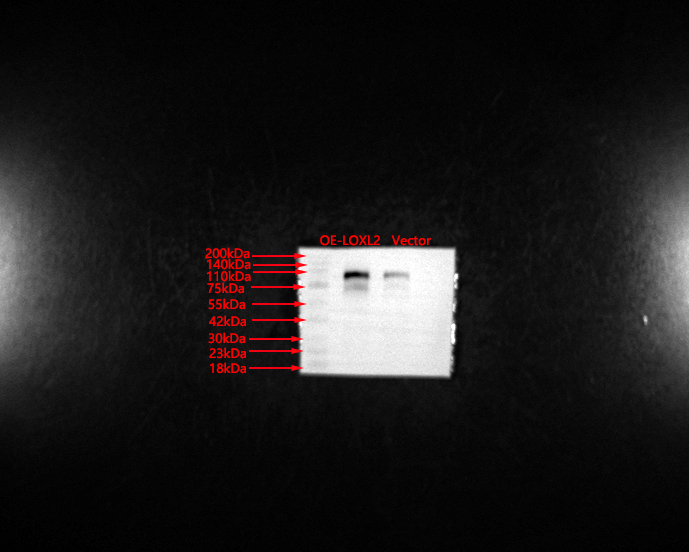


GAPDH


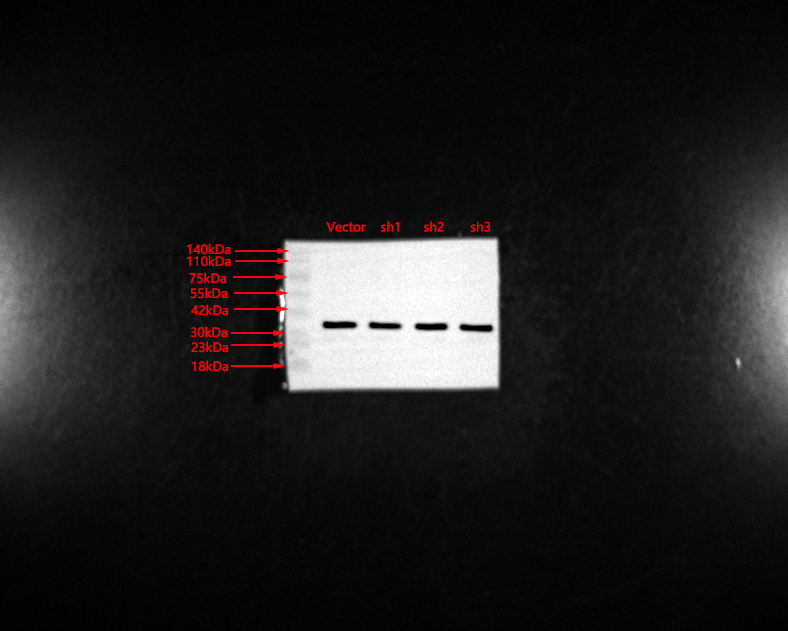


Sh-LOXL2


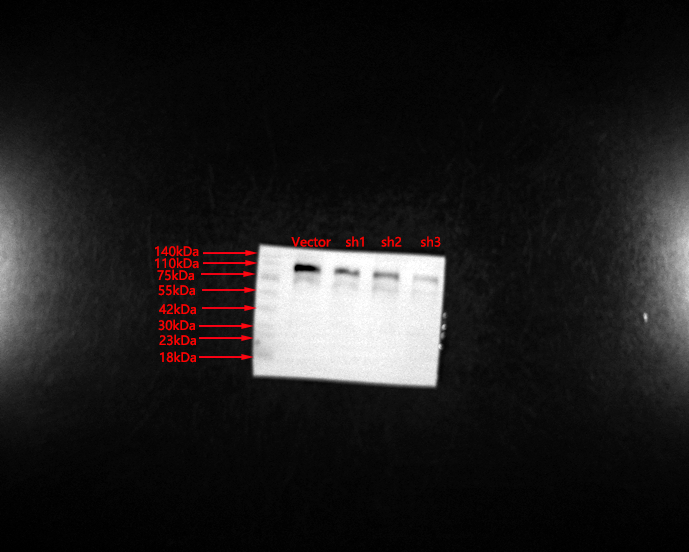


**Figure 6C**

OE-PBX3


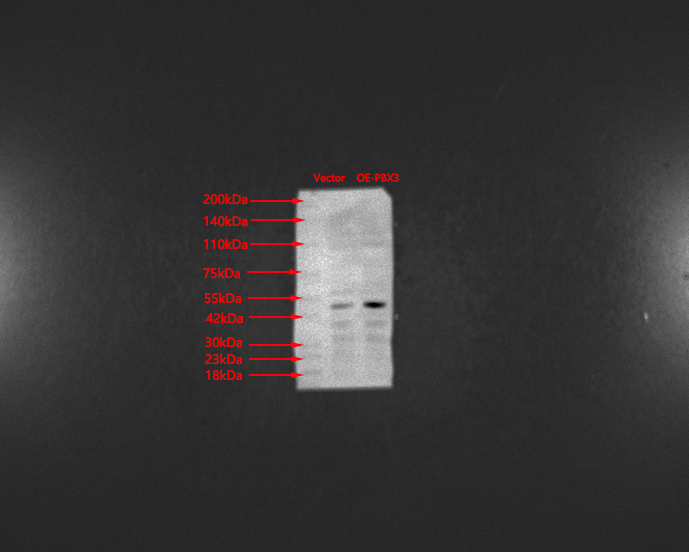


GAPDH


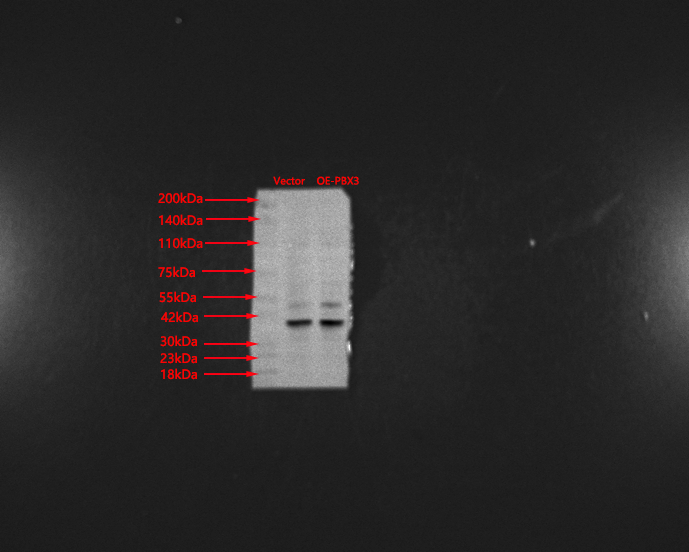


OE-ELF3


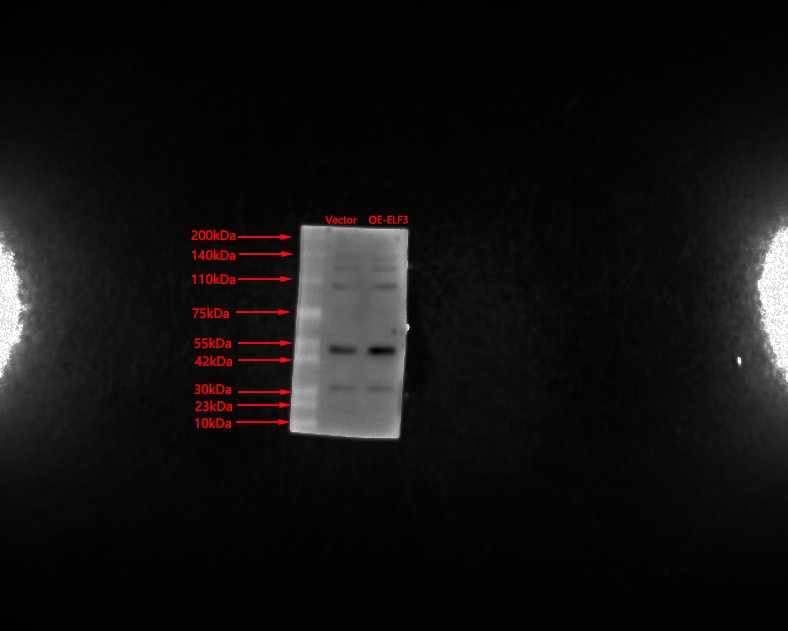


GAPDH


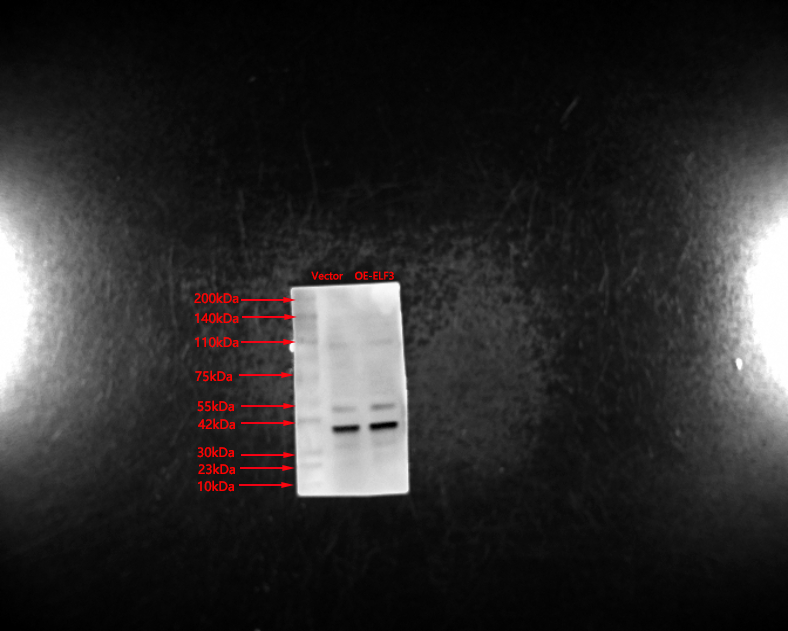


OE-E2F1


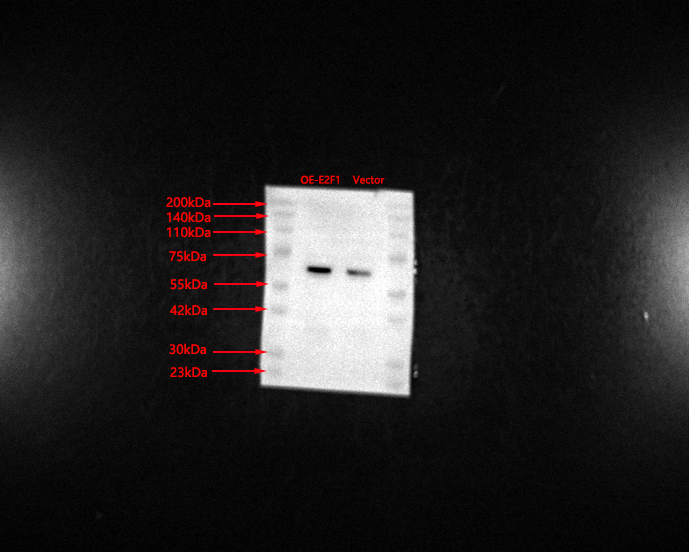


GAPDH


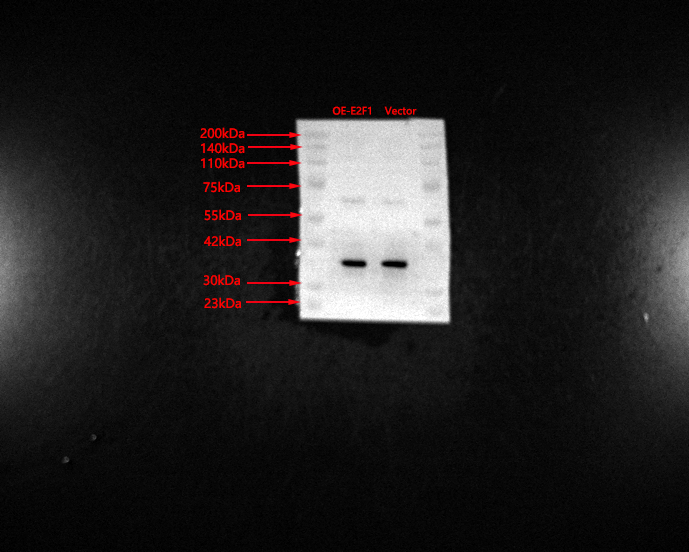


OE-KLF10


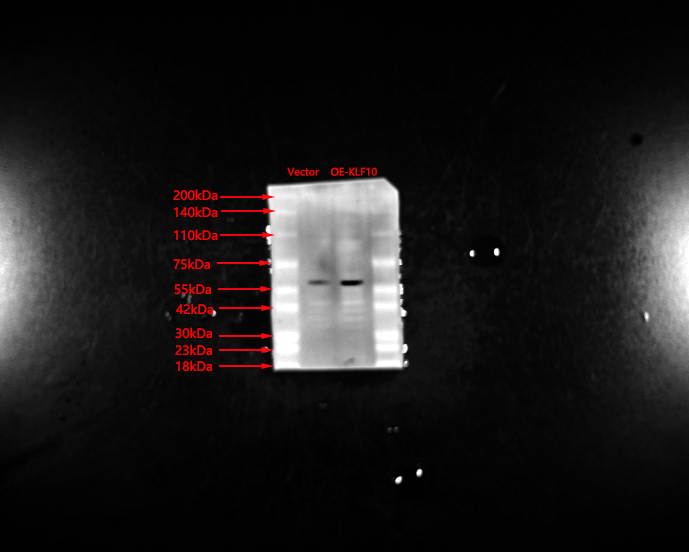


GAPDH


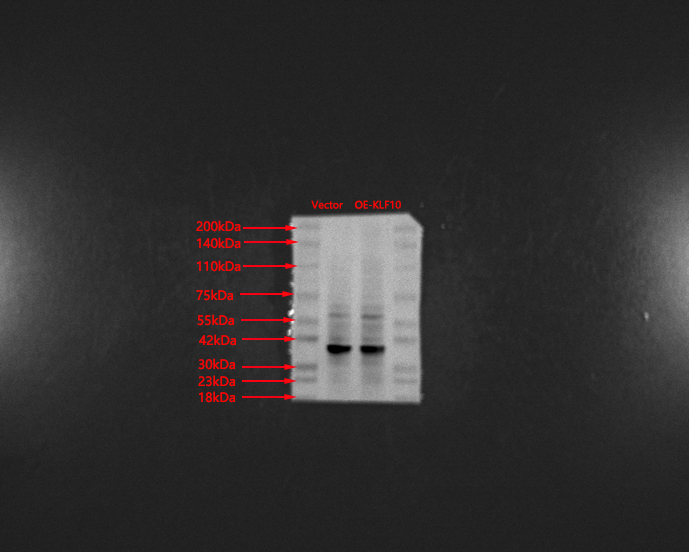


LOXL2


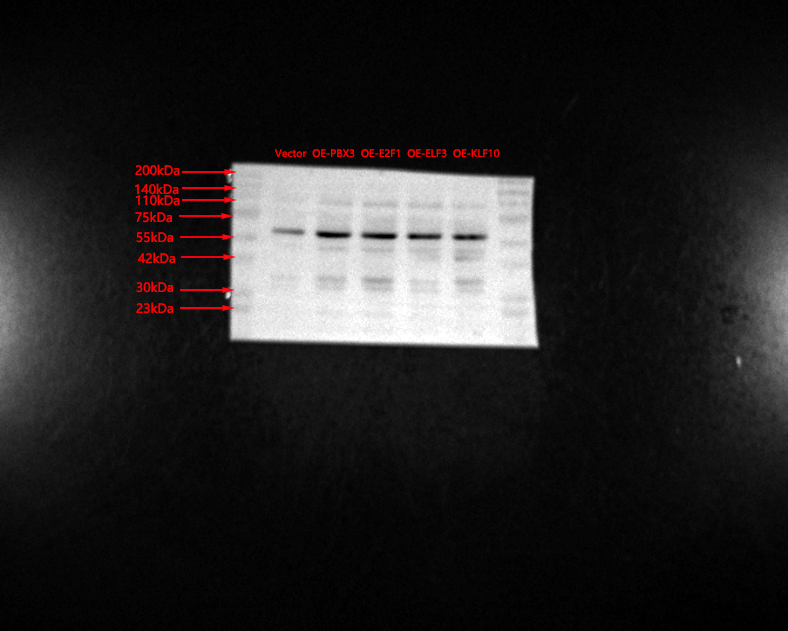


GAPDH


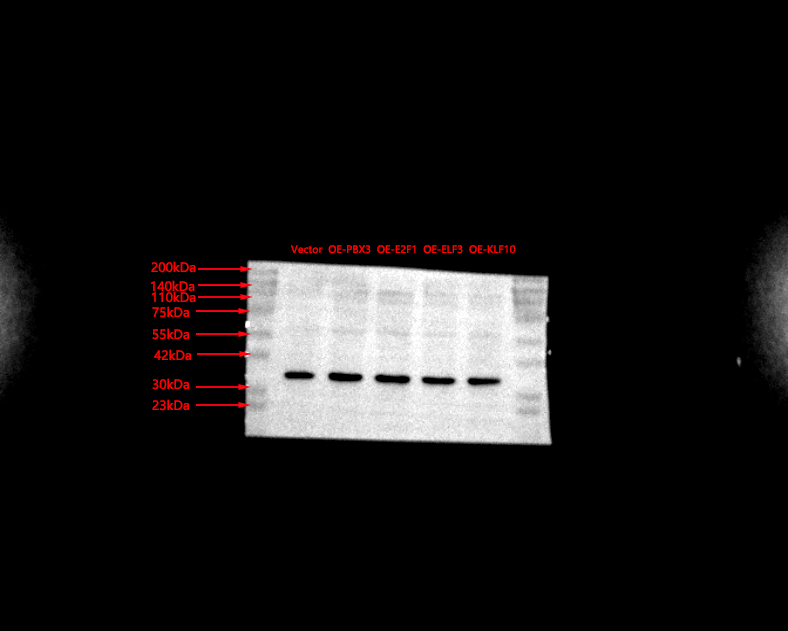


**Figure 6M**

KLF10


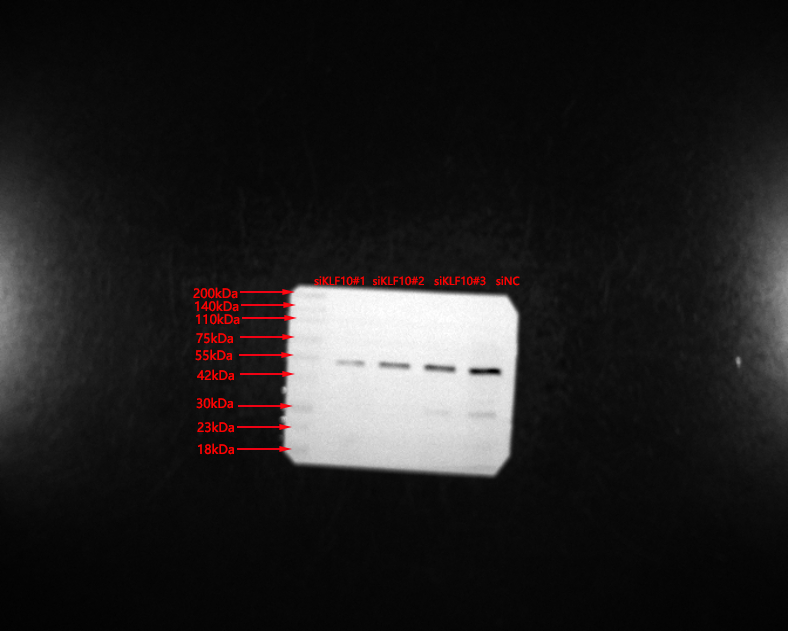


GAPDH


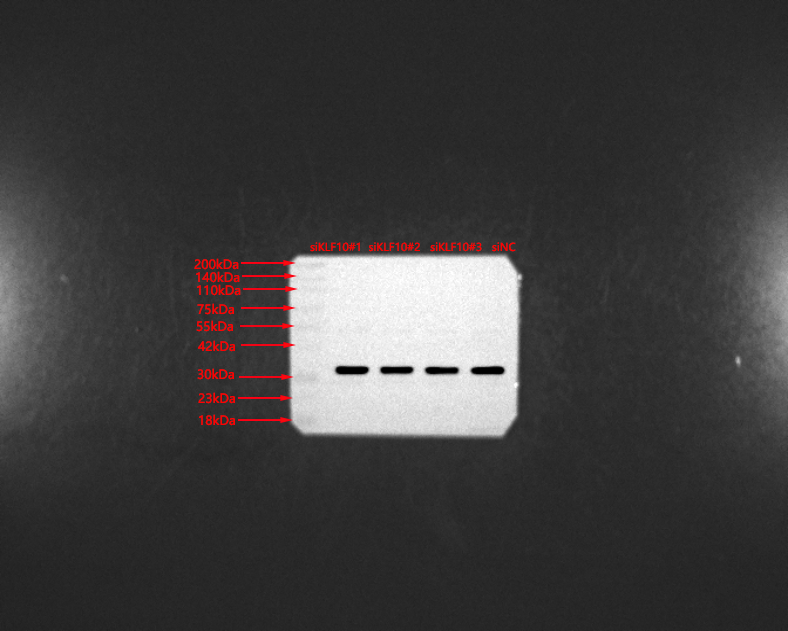


**Figure 6O**

LOXL2

**
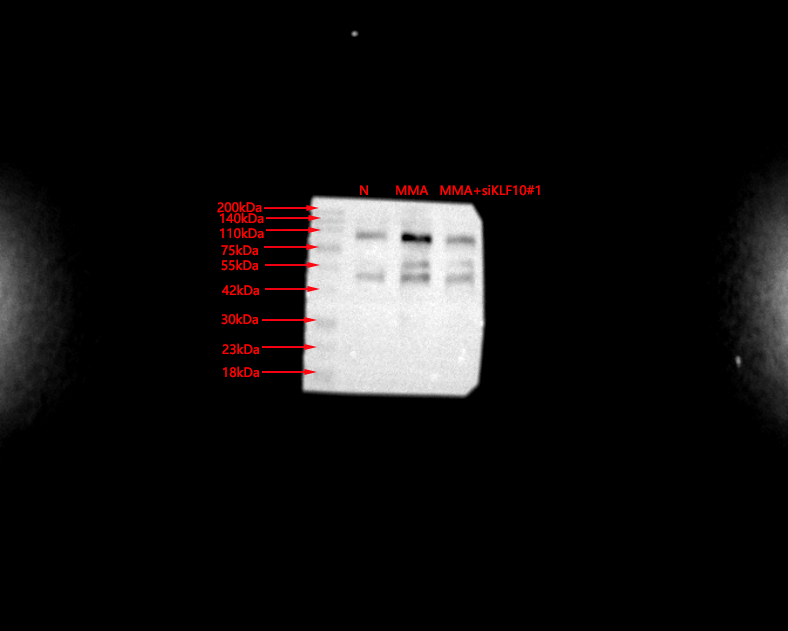
**

GAPDH

**
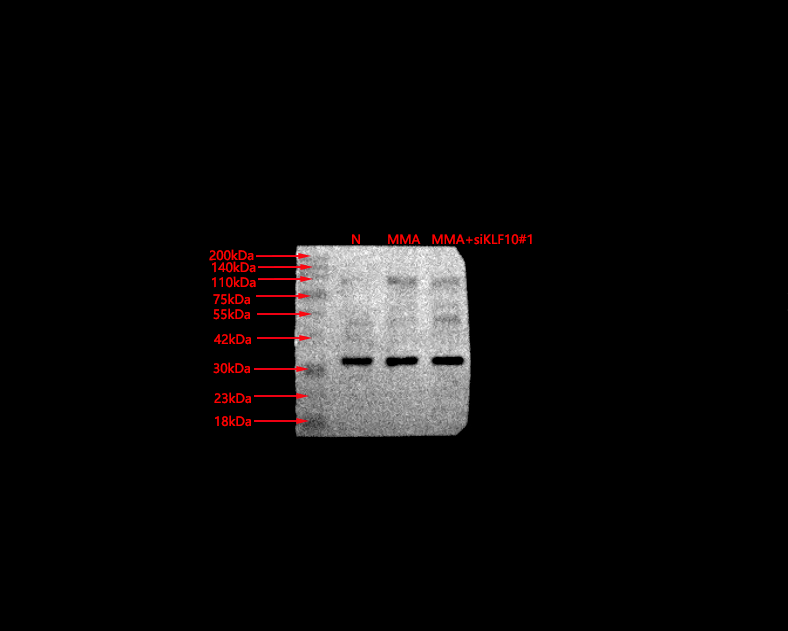
**

KLF10


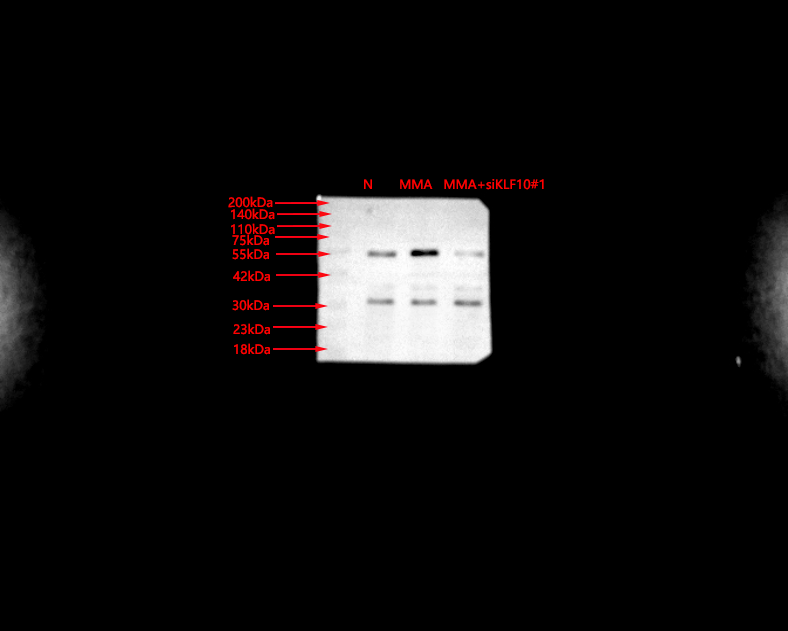


GAPDH


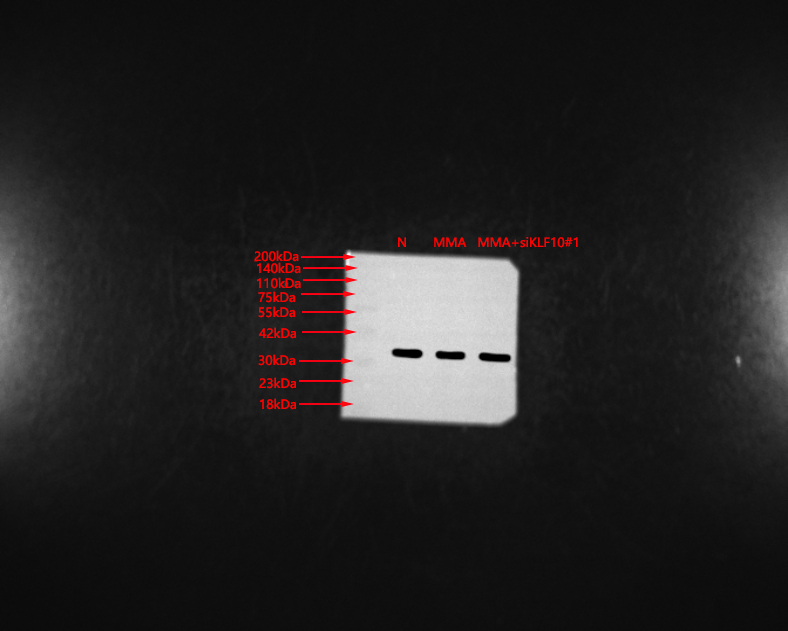


**Figure 7B**

Upper left


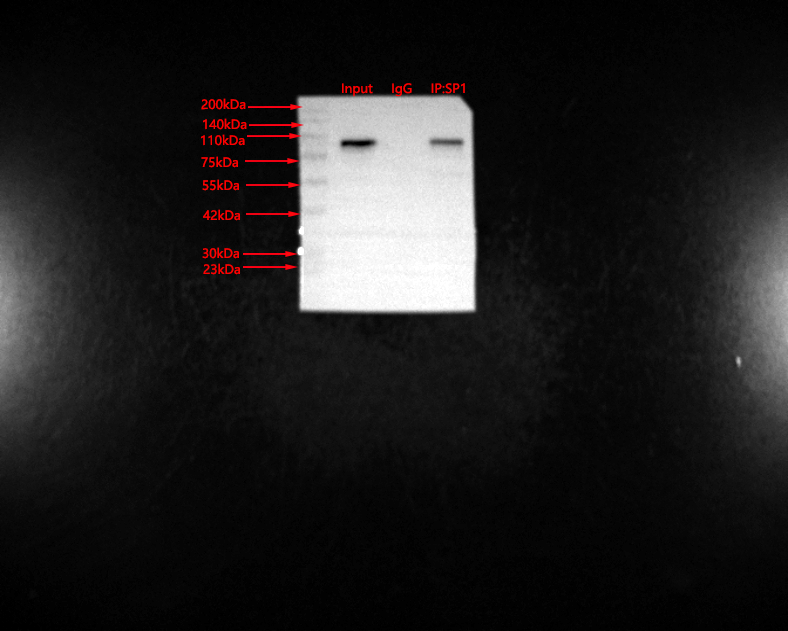


Bottom left


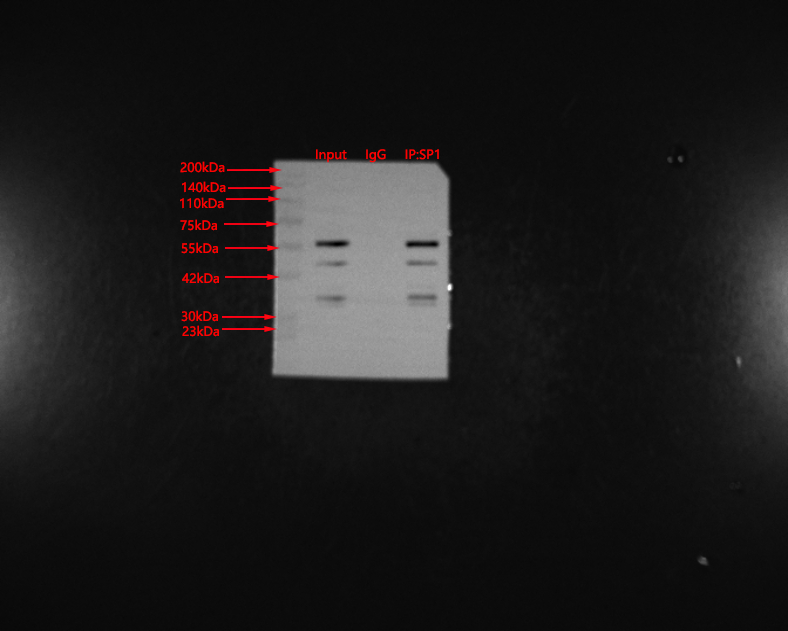


Upper right


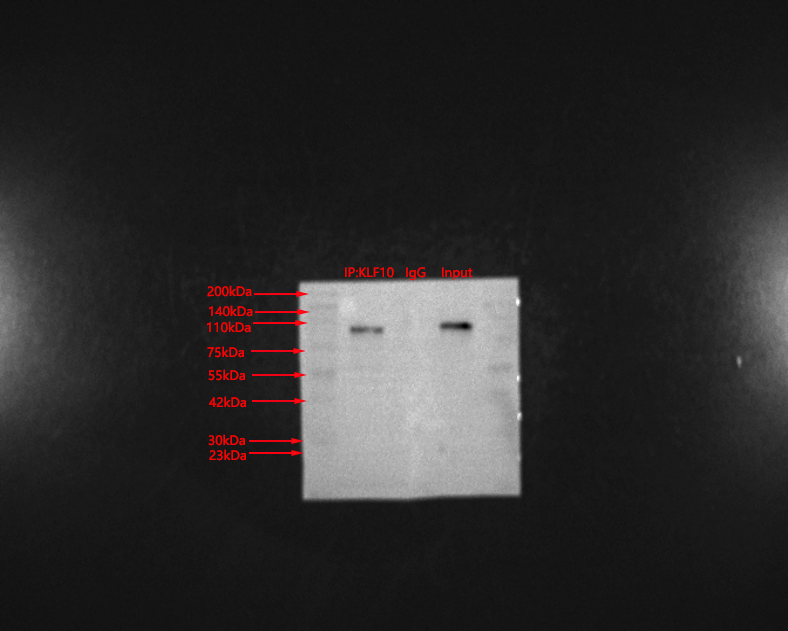


Bottom right


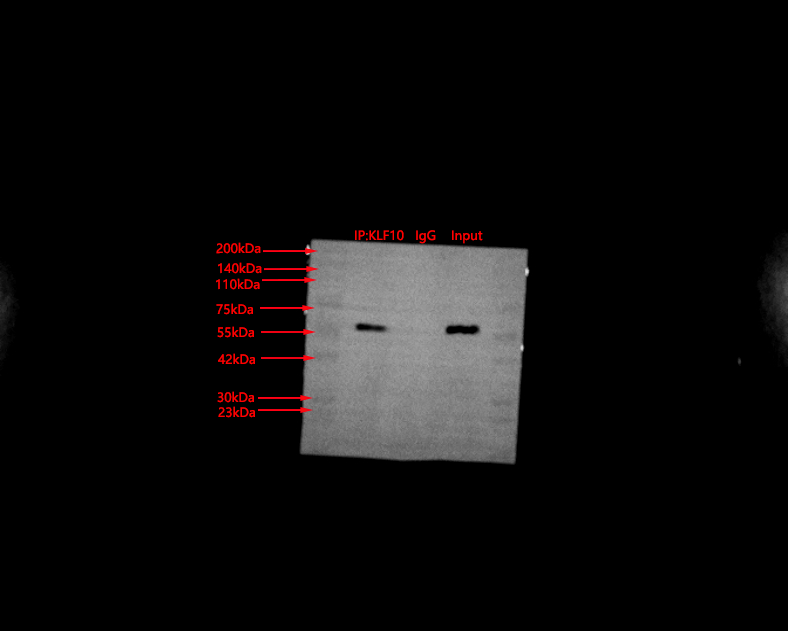


**Figure 7G**

KLF10


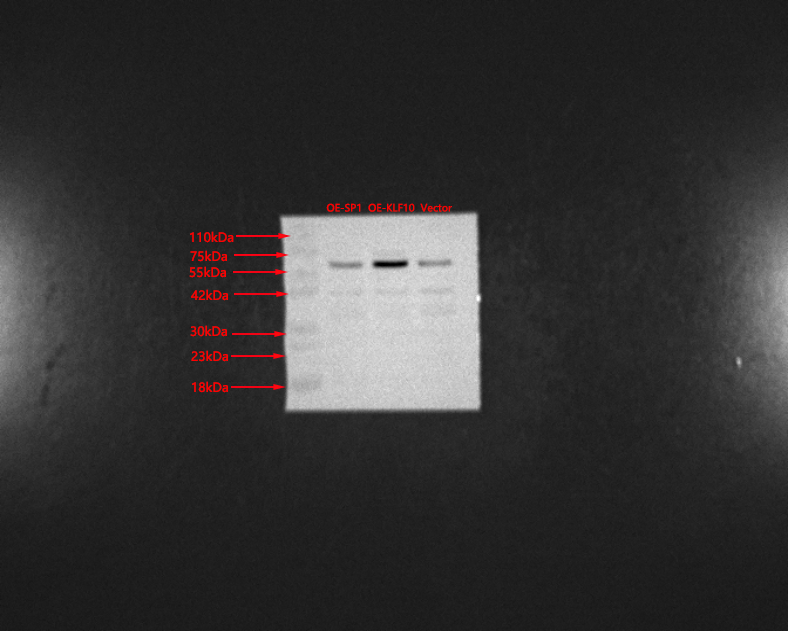


GAPDH


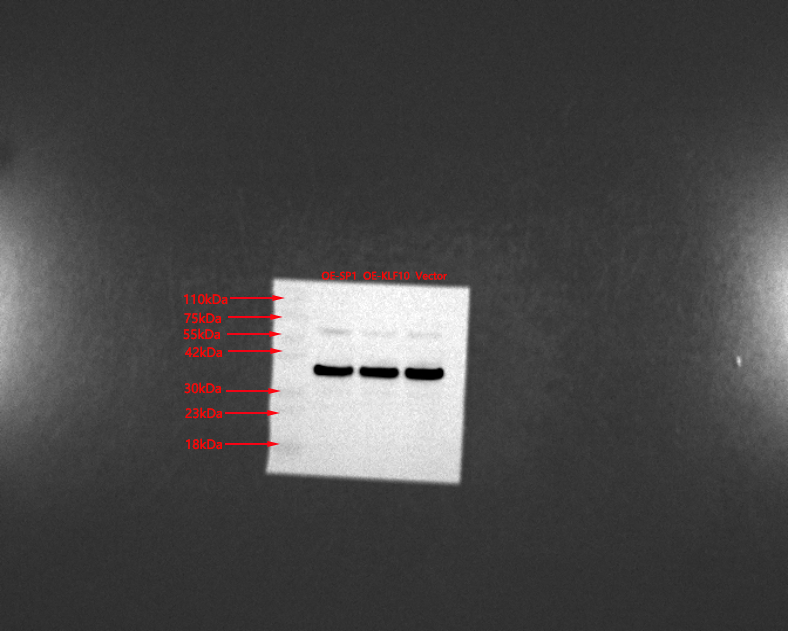


SP1


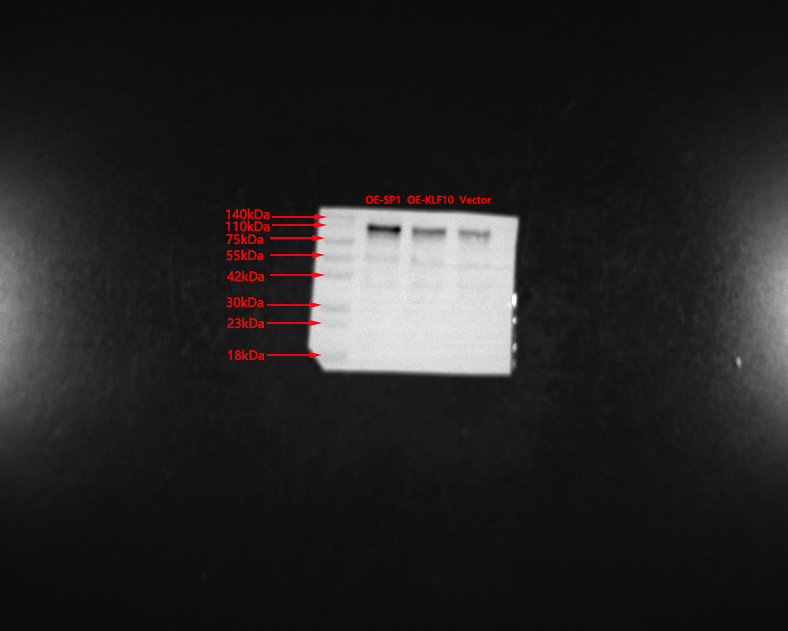


GAPDH


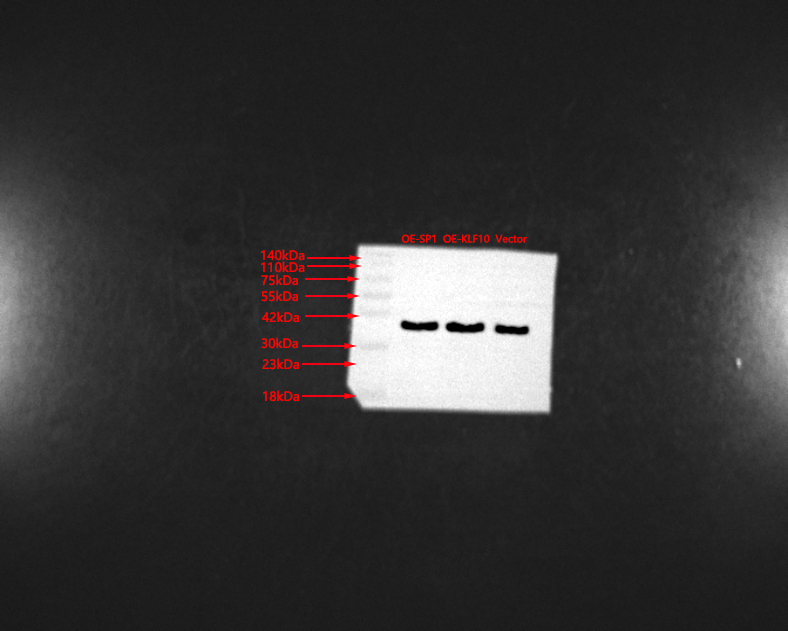


**Figure S3C**

COL I


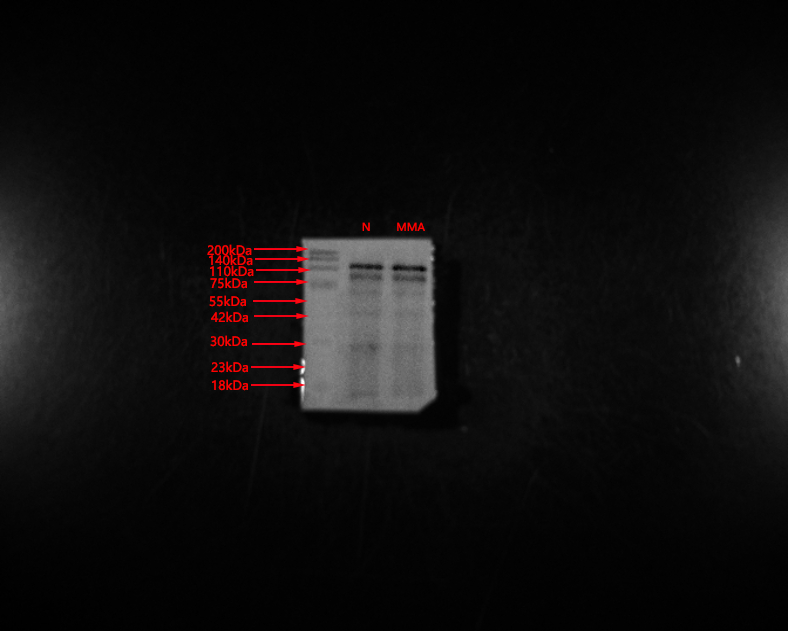


GAPDH


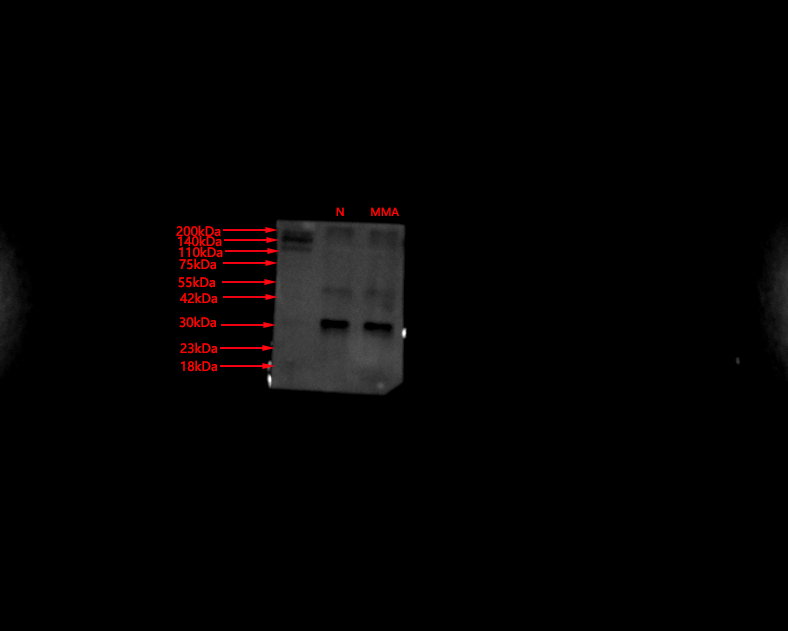


**Figure S5E**

COL I


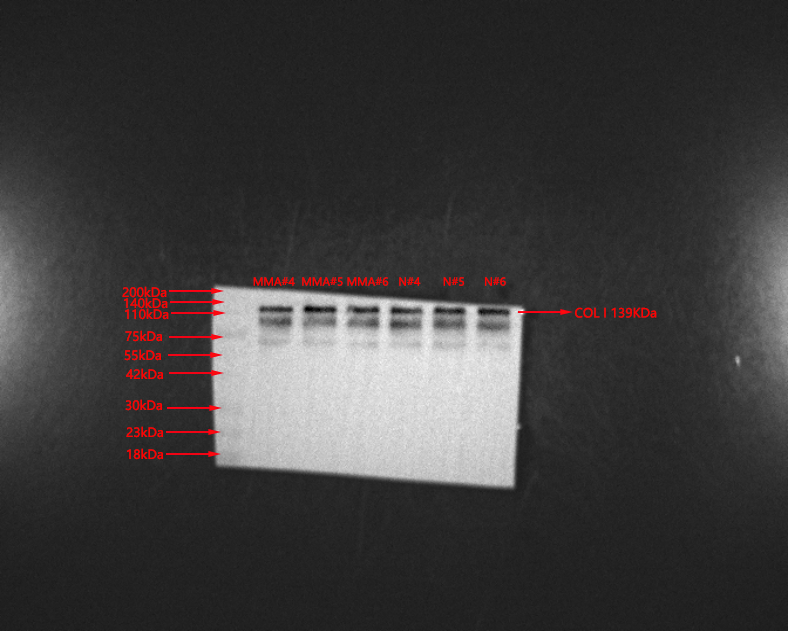


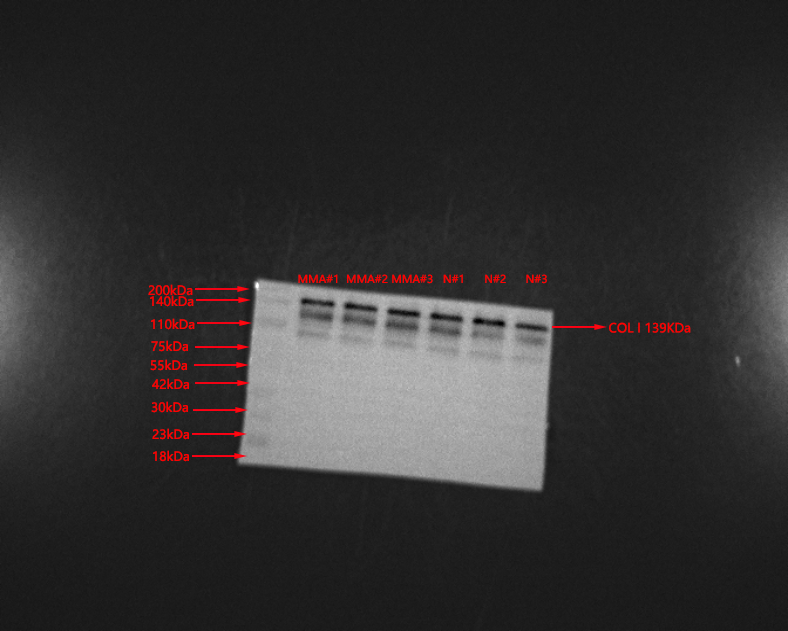


GAPDH


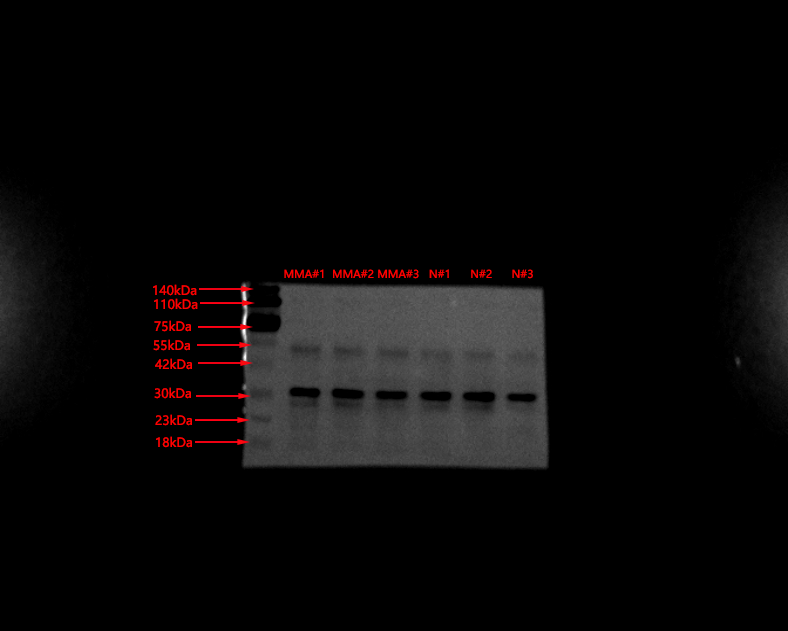


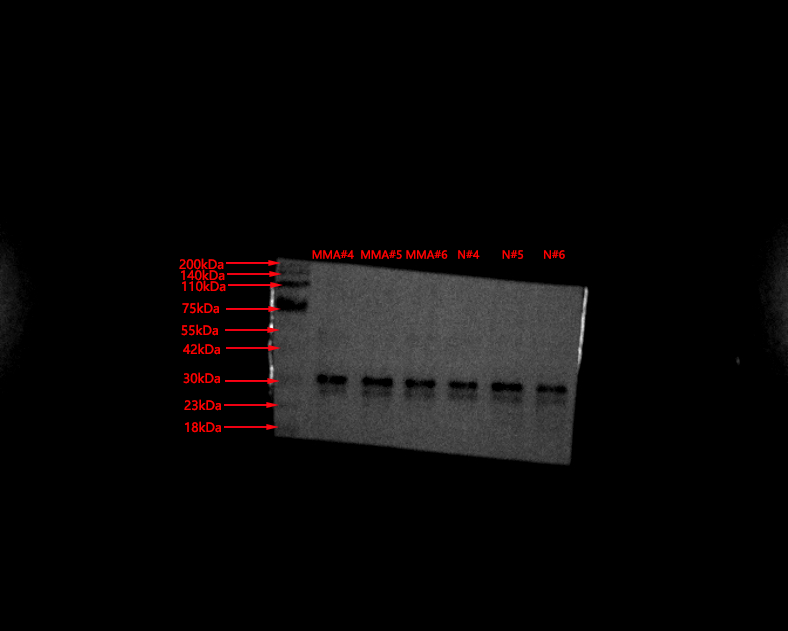


αSMA


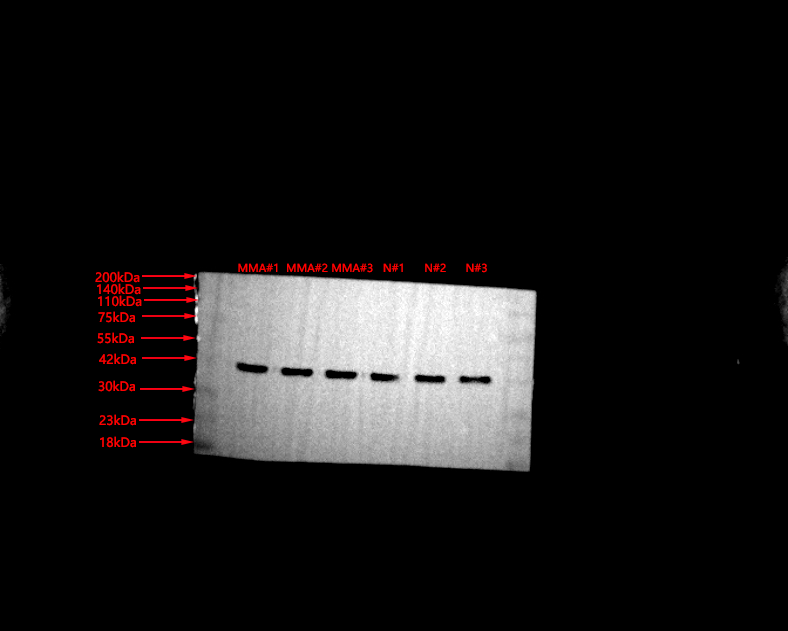


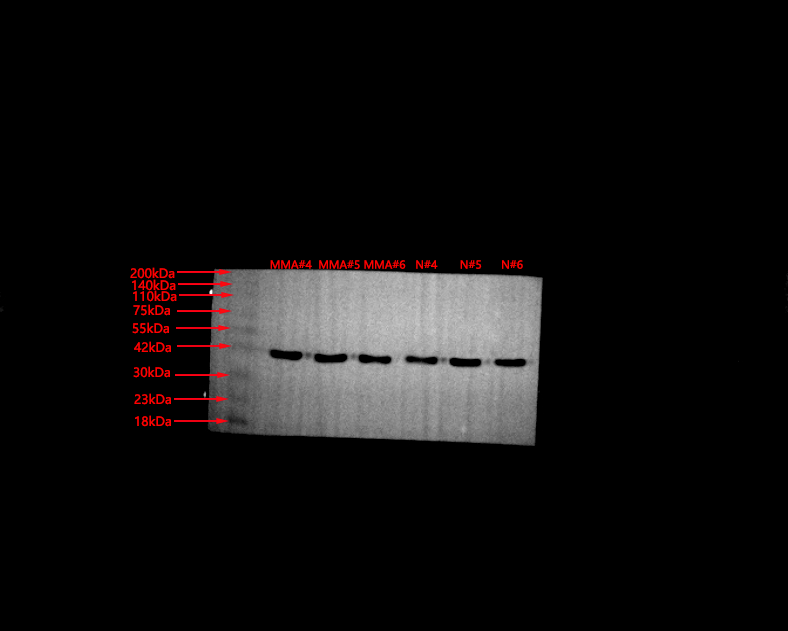


GAPDH


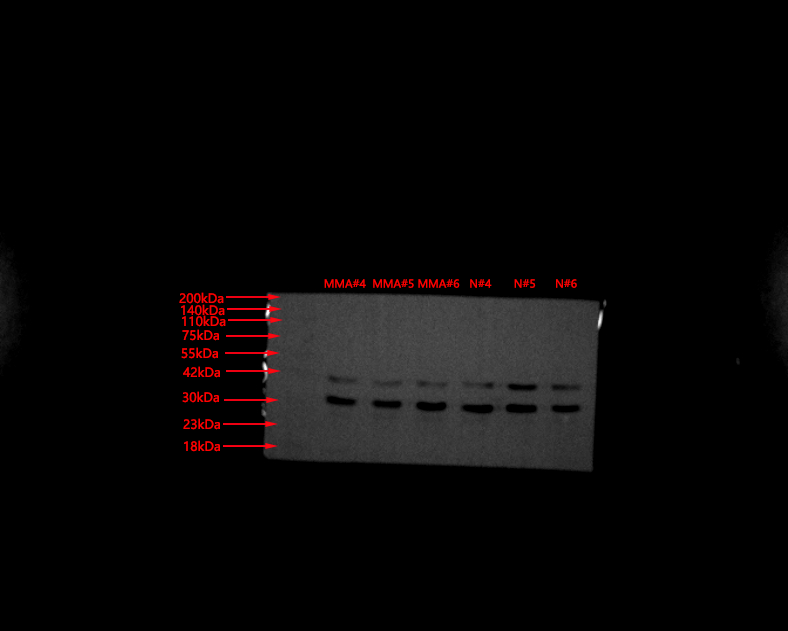


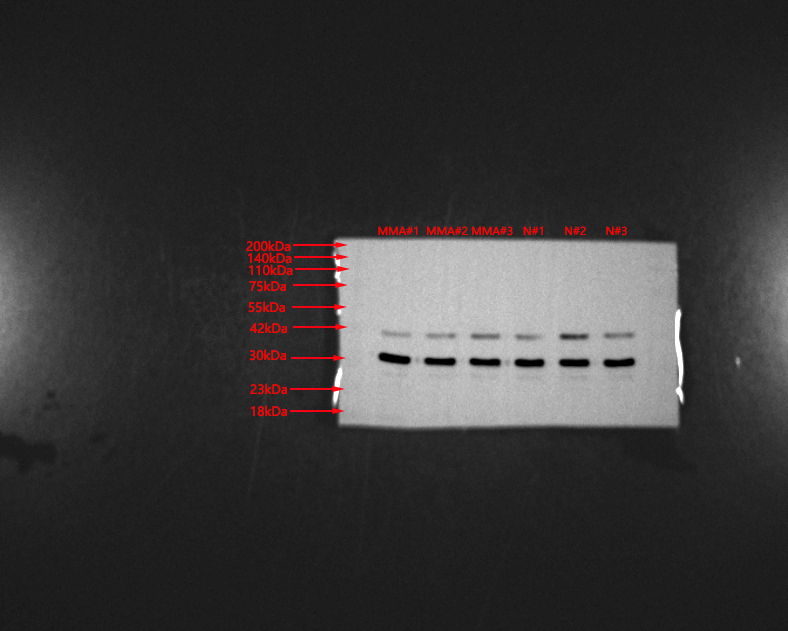


LOXL2


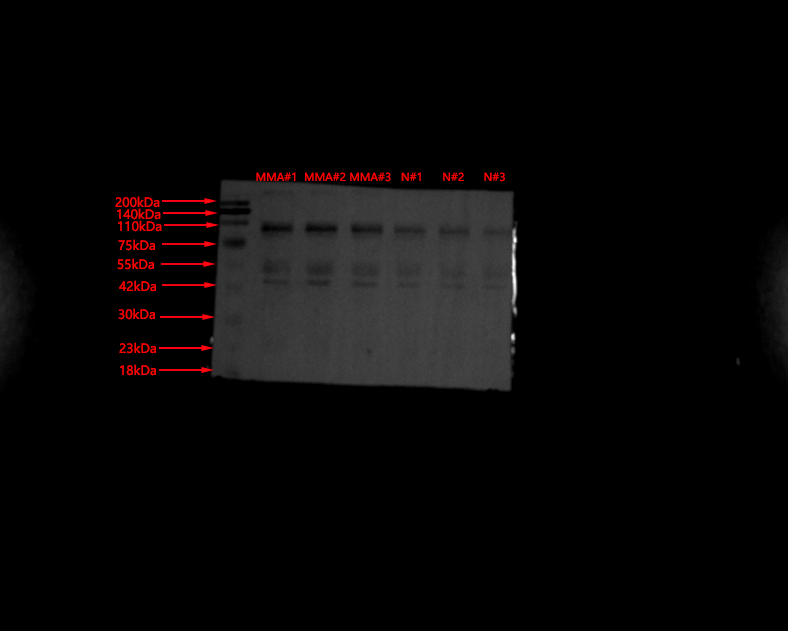


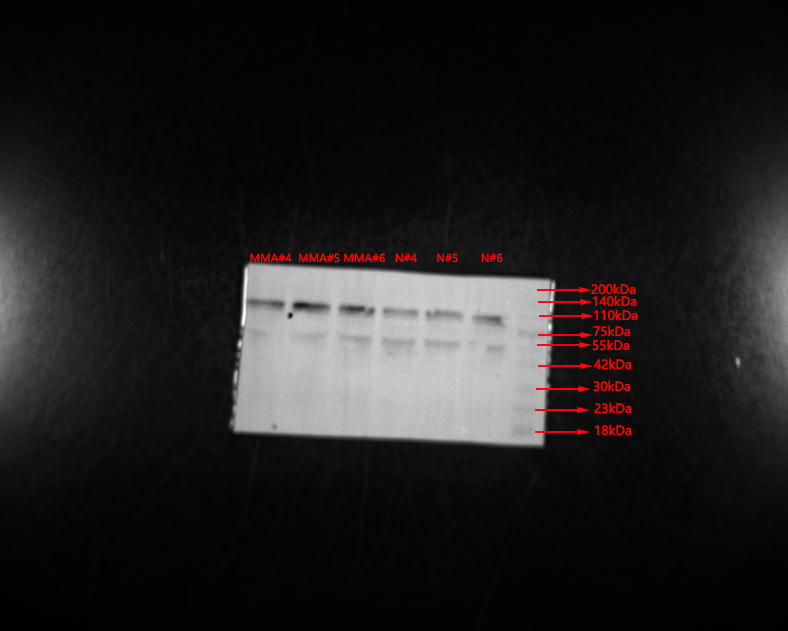


GAPDH


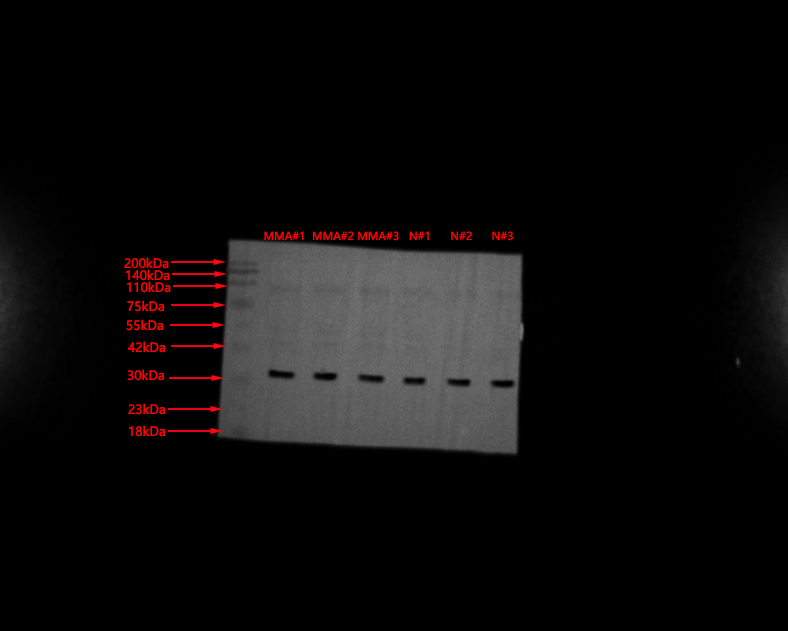


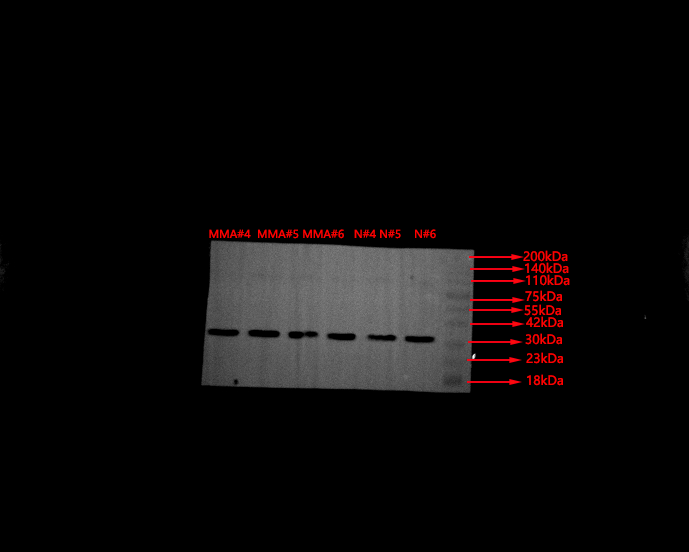


**Figure S5K**

LOXL2


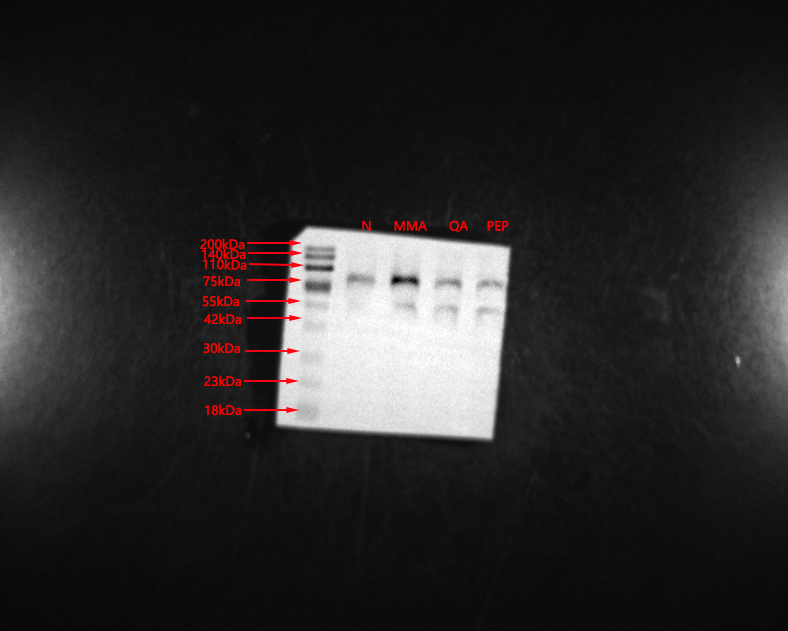


GAPDH


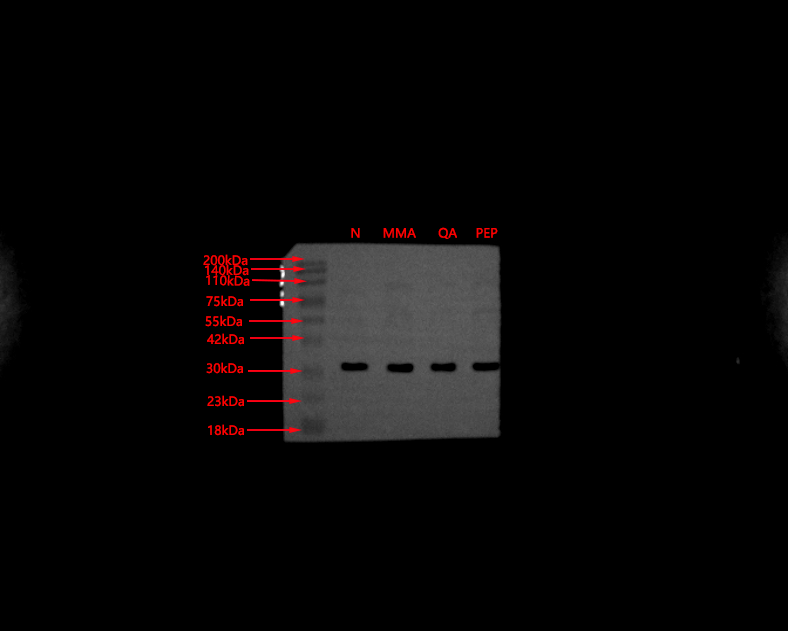


**Figure S6B**

COL I


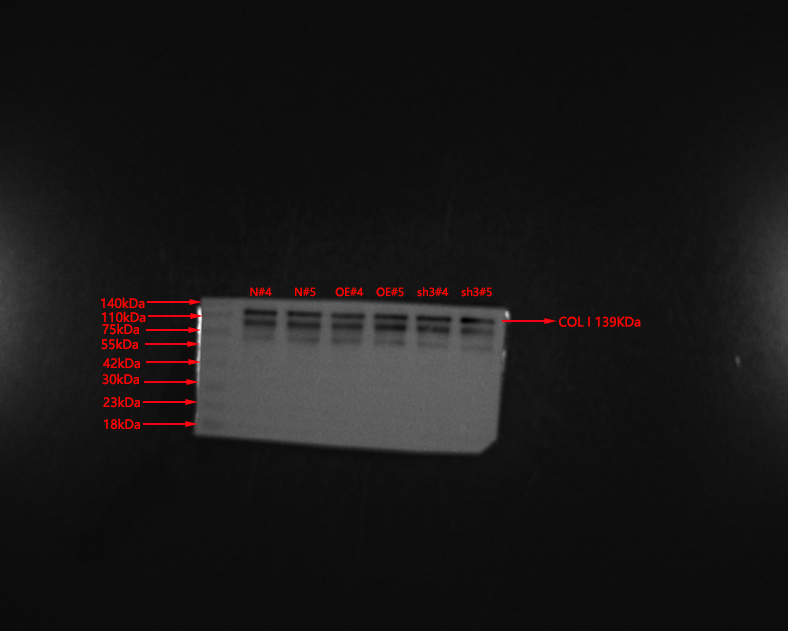


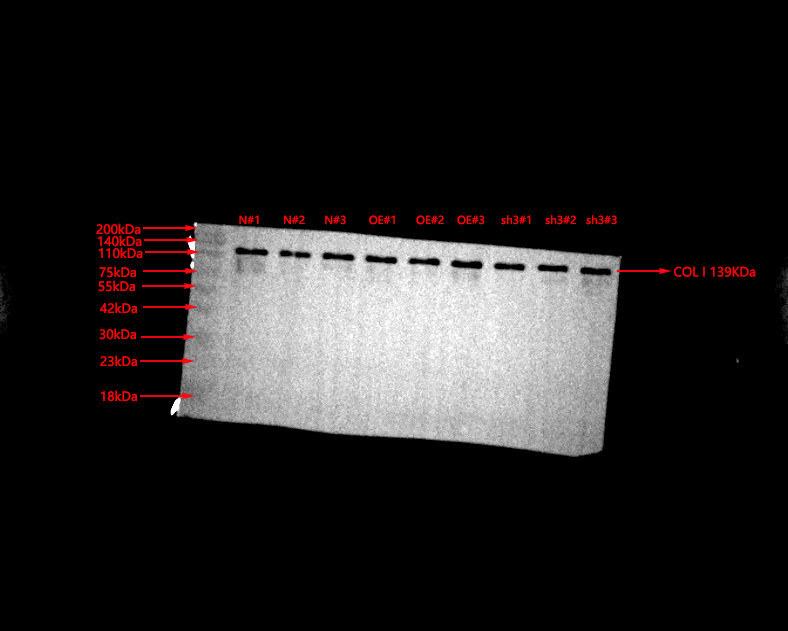


GAPDH


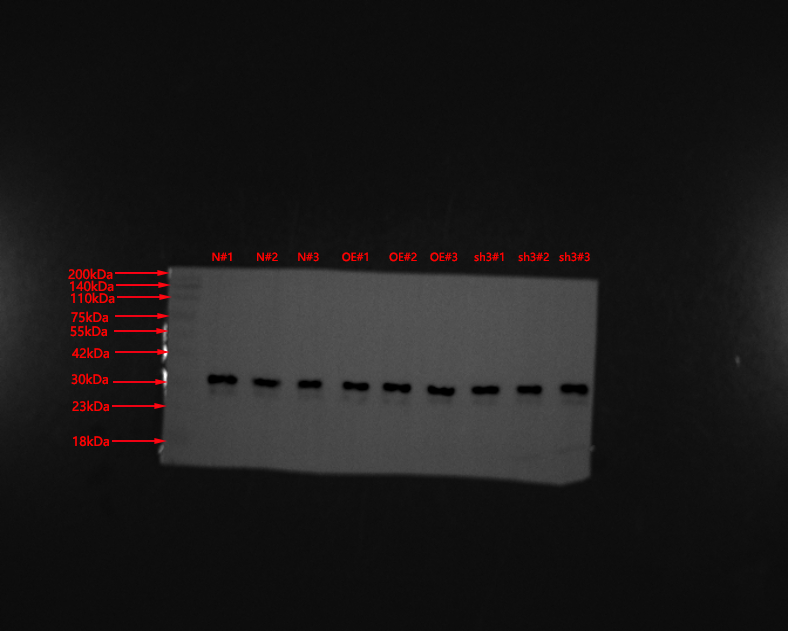


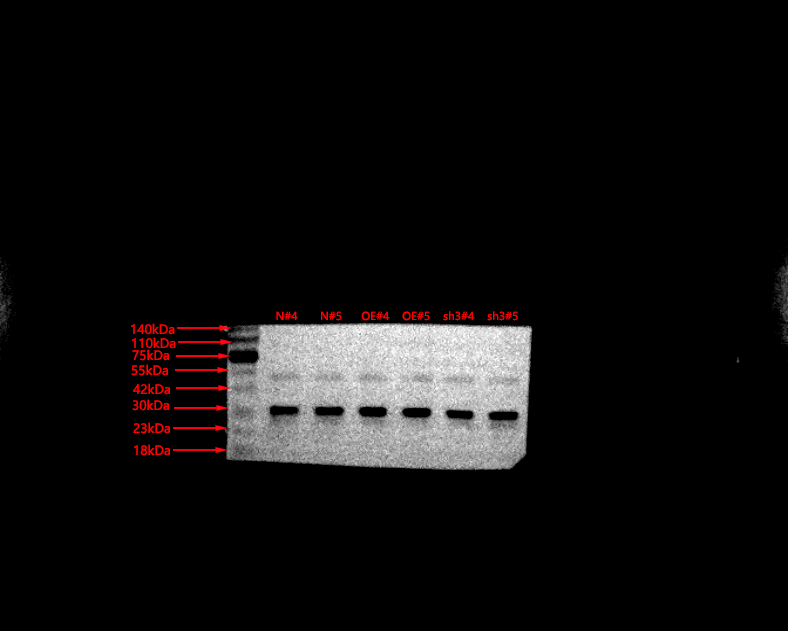


αSMA


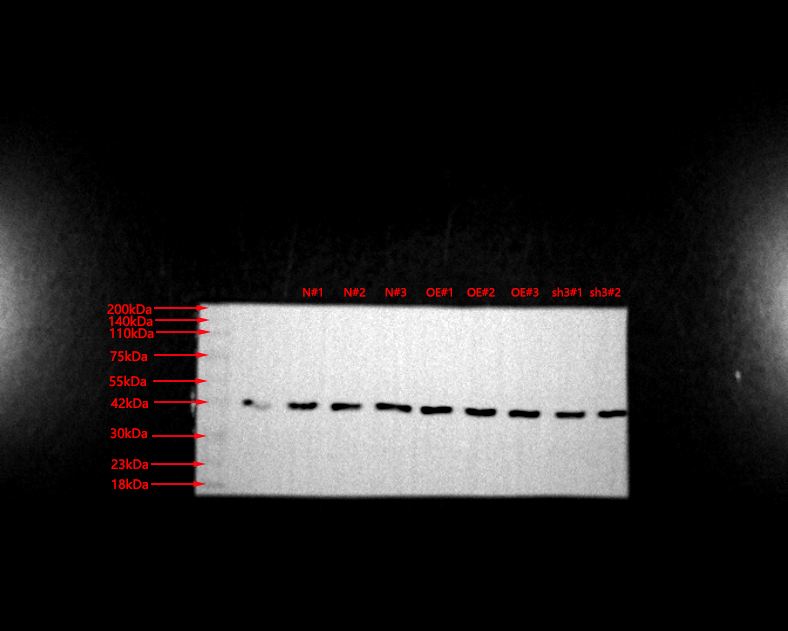


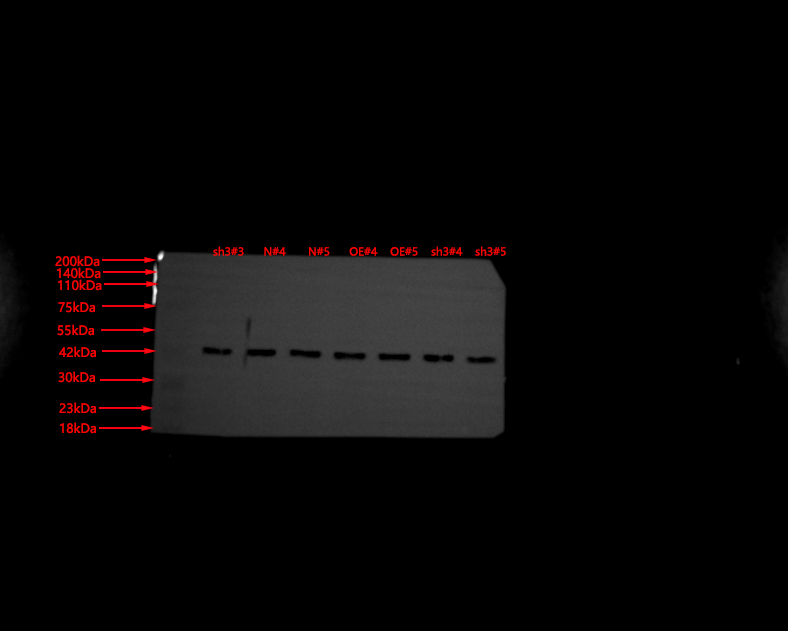


GAPDH


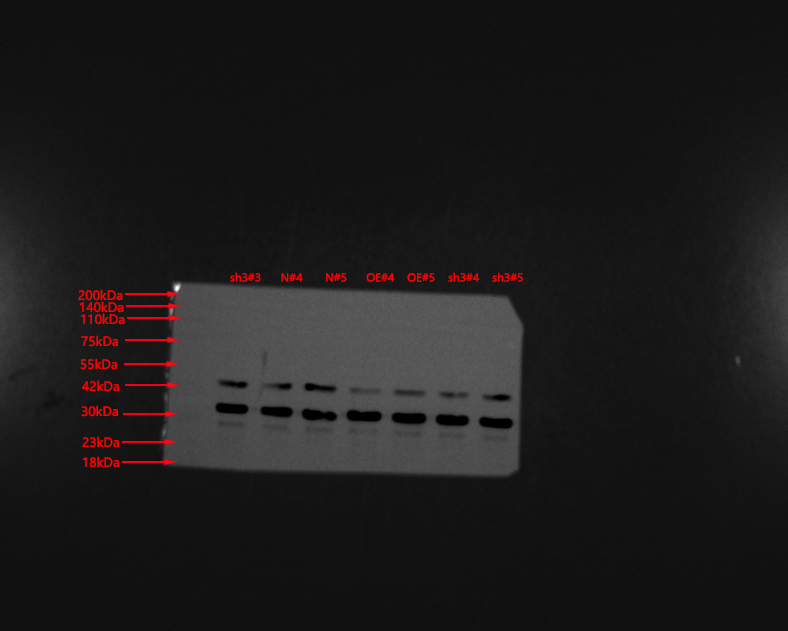


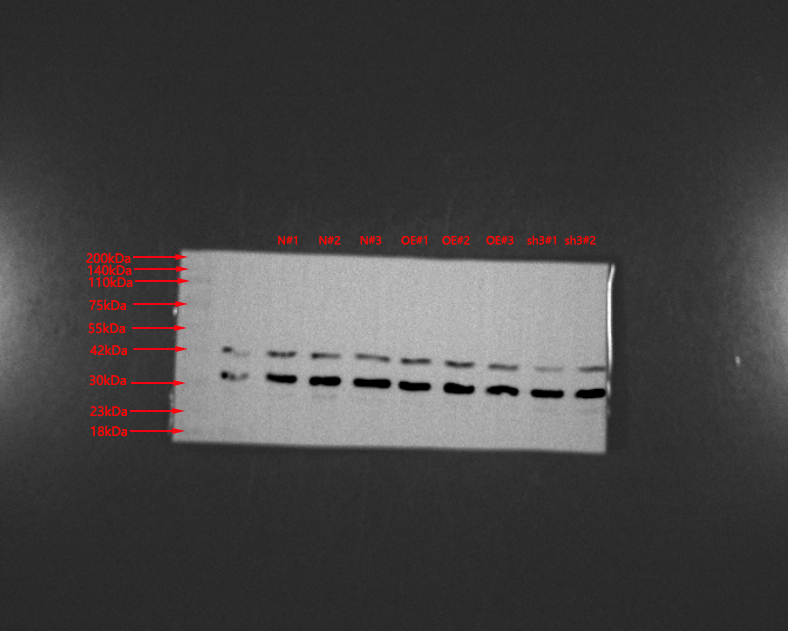


LOXL2


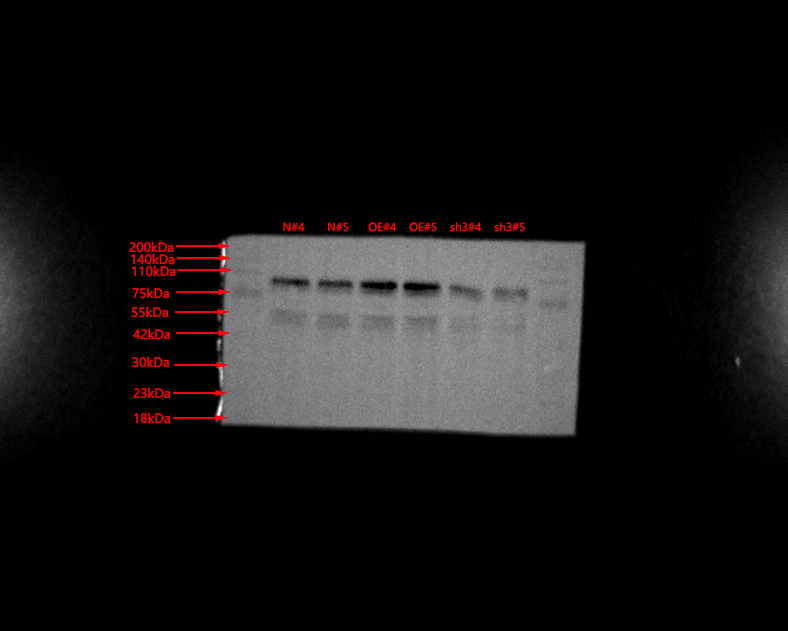


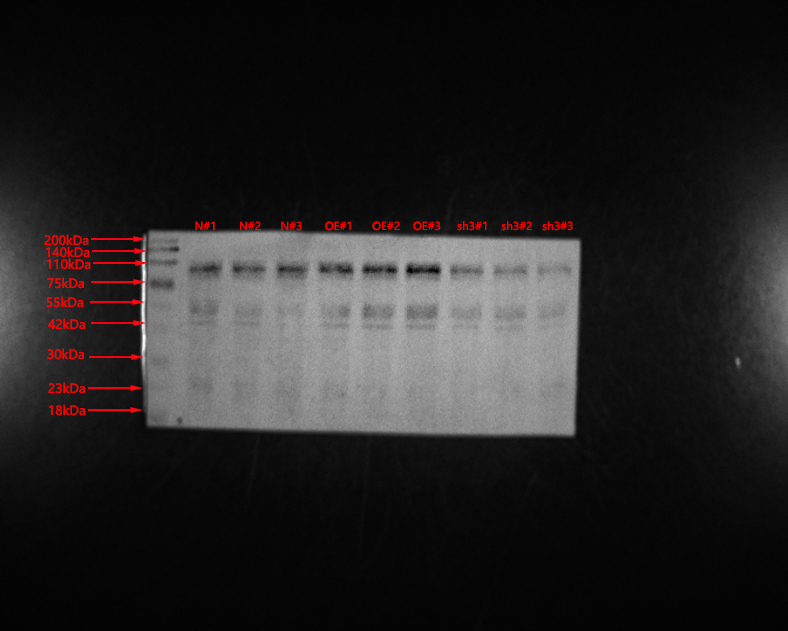


GAPDH


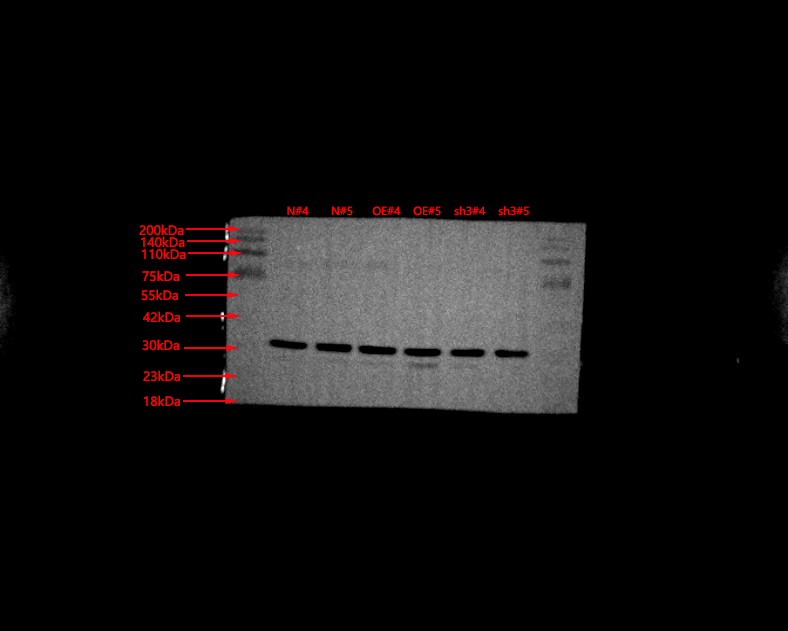


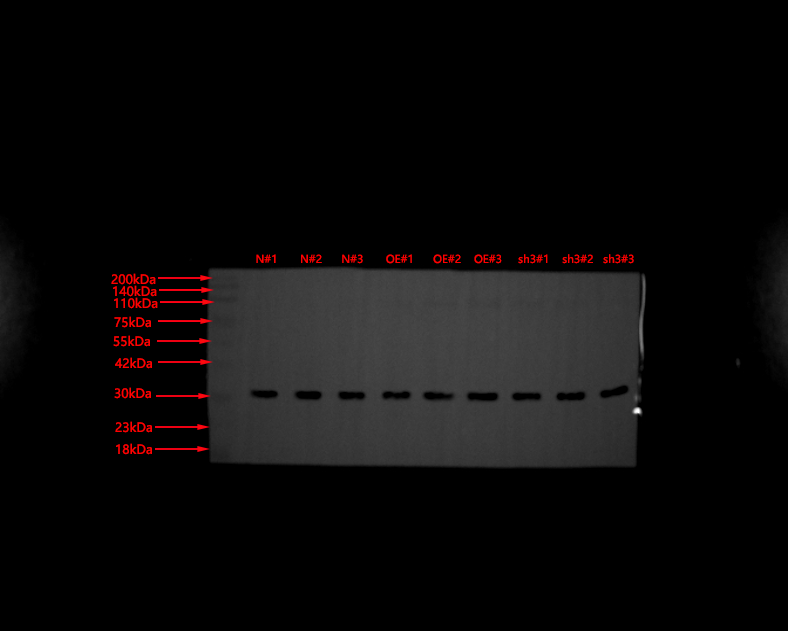


**Figure S7A**

LOXL2


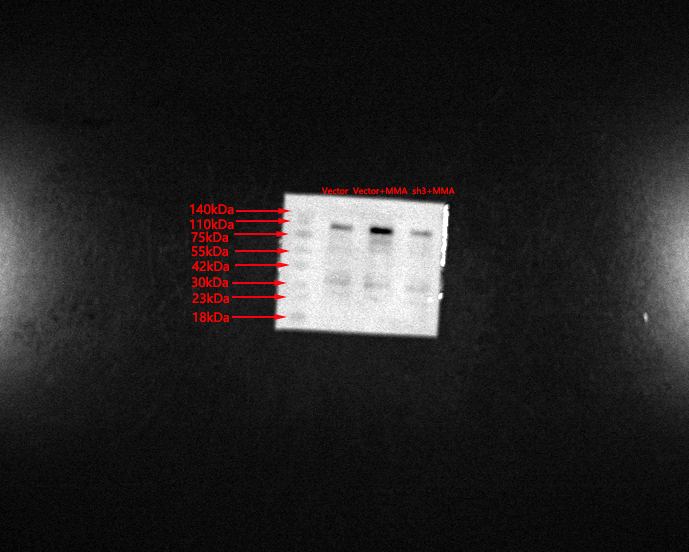


GAPDH


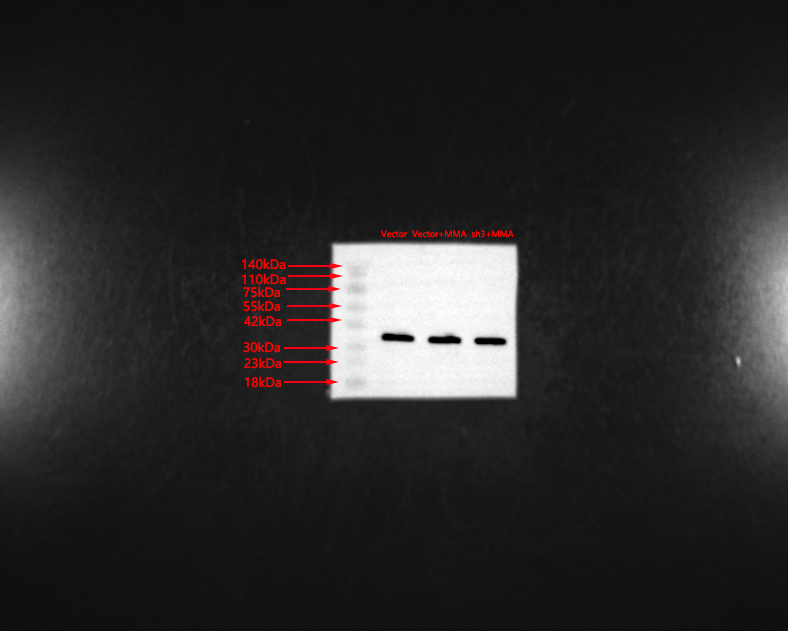


**Figure S7G**

COL I


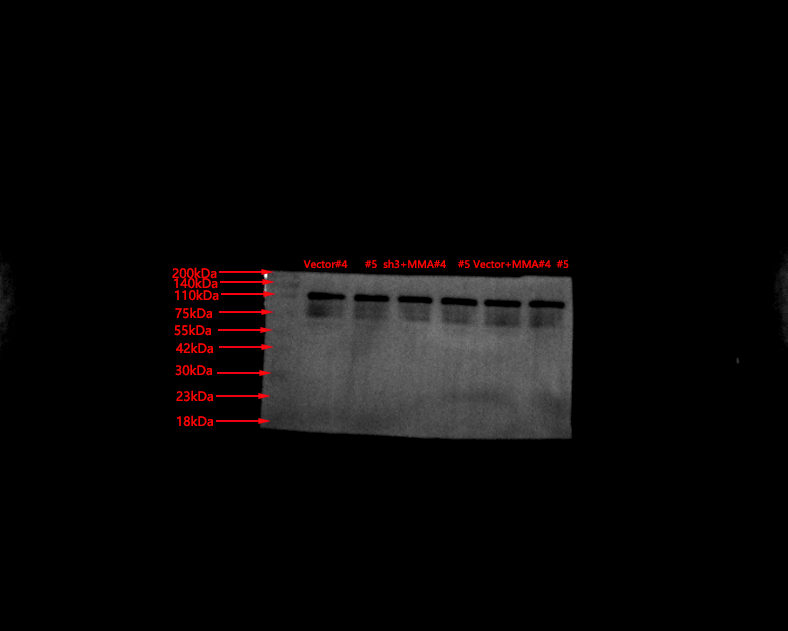


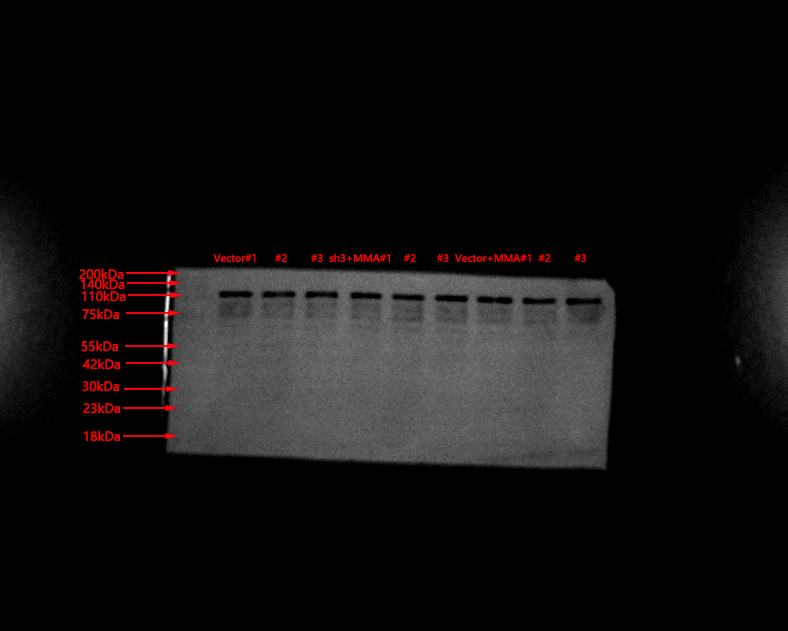


GAPDH


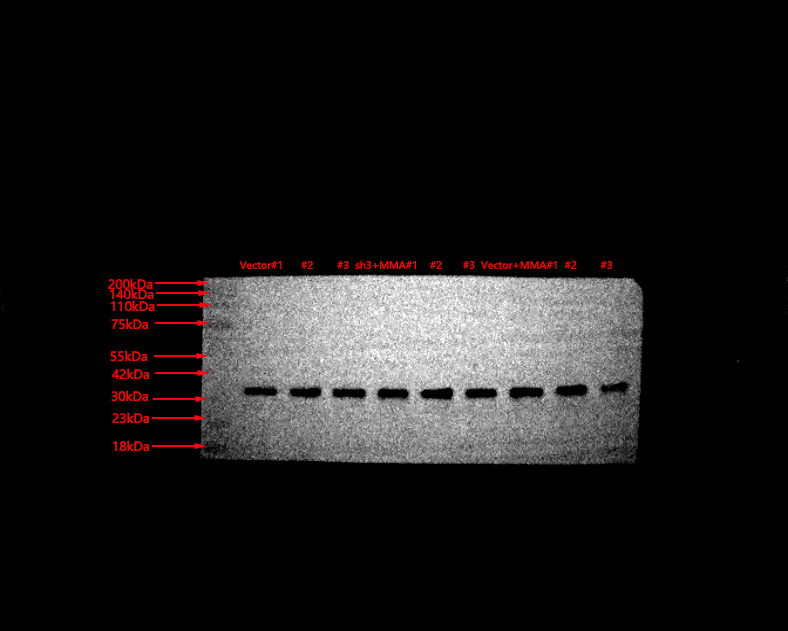


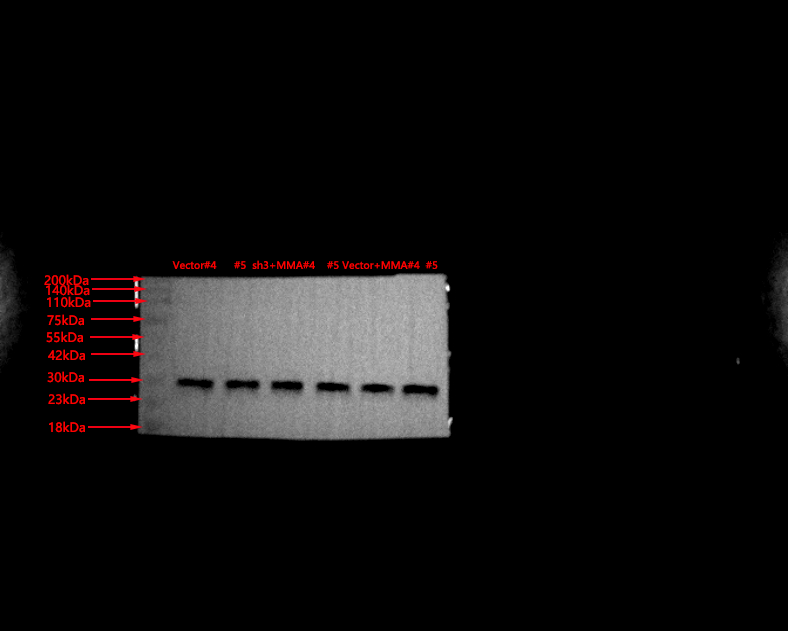


αSMA


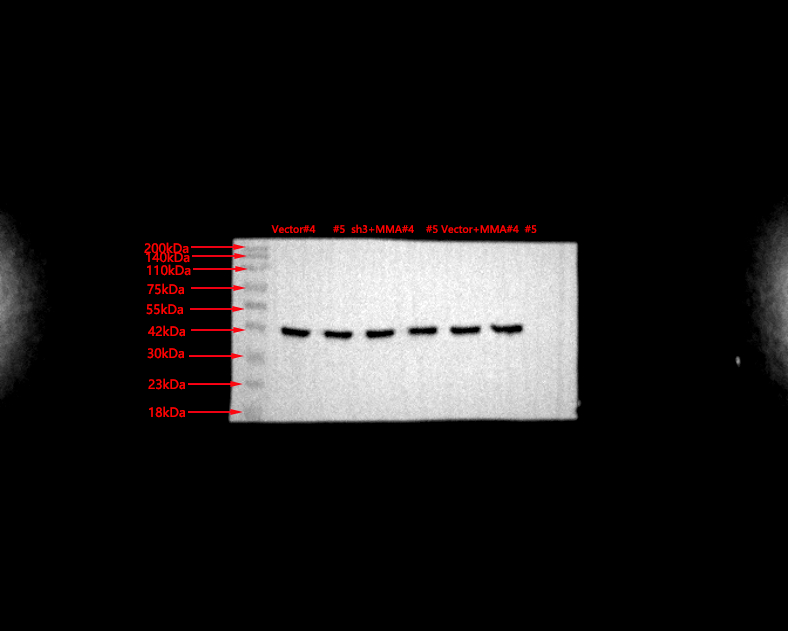


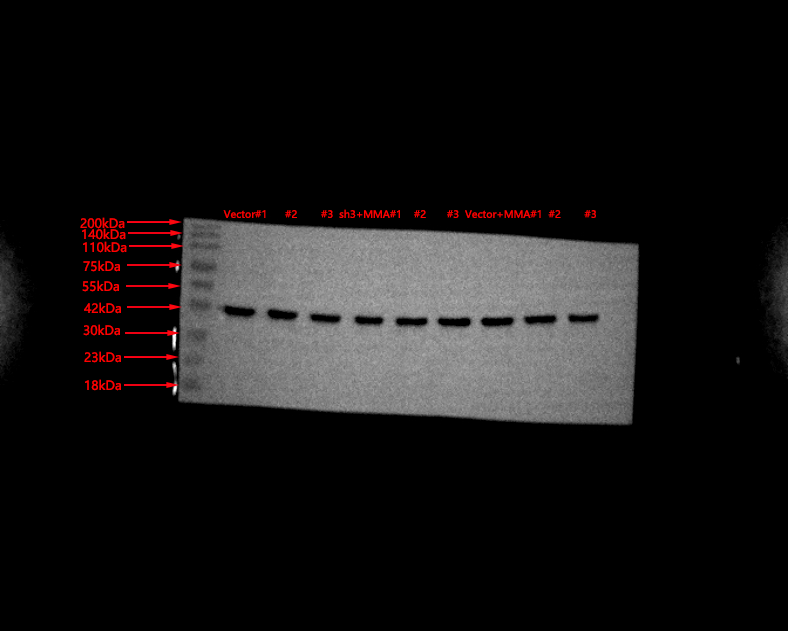


GAPDH


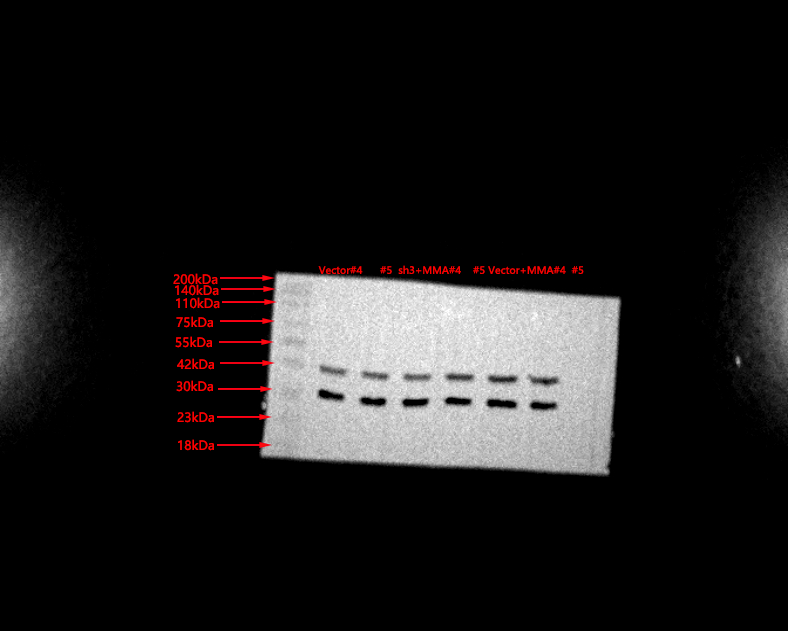


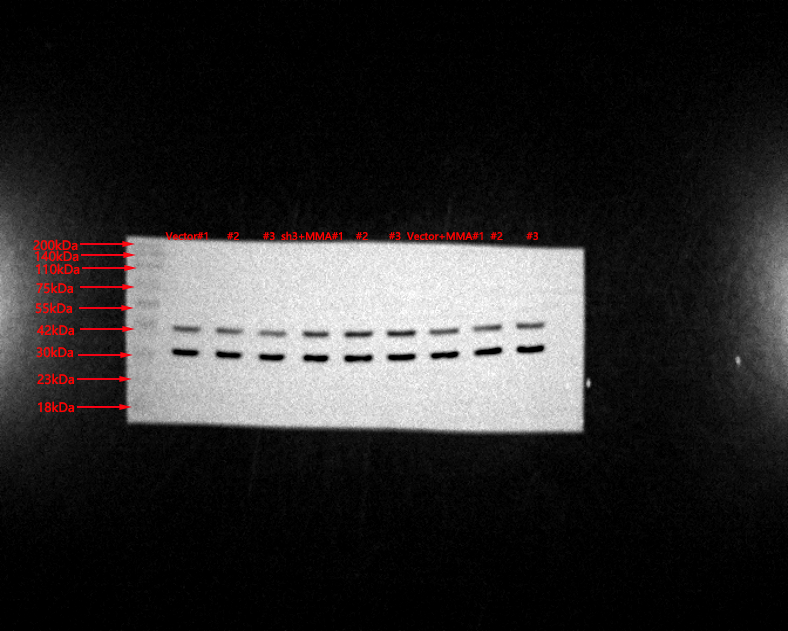


LOXL2


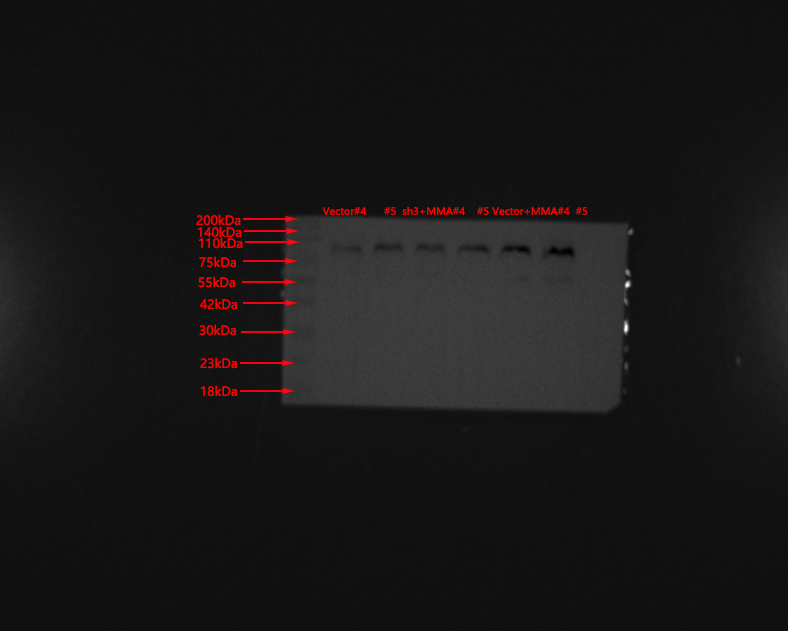


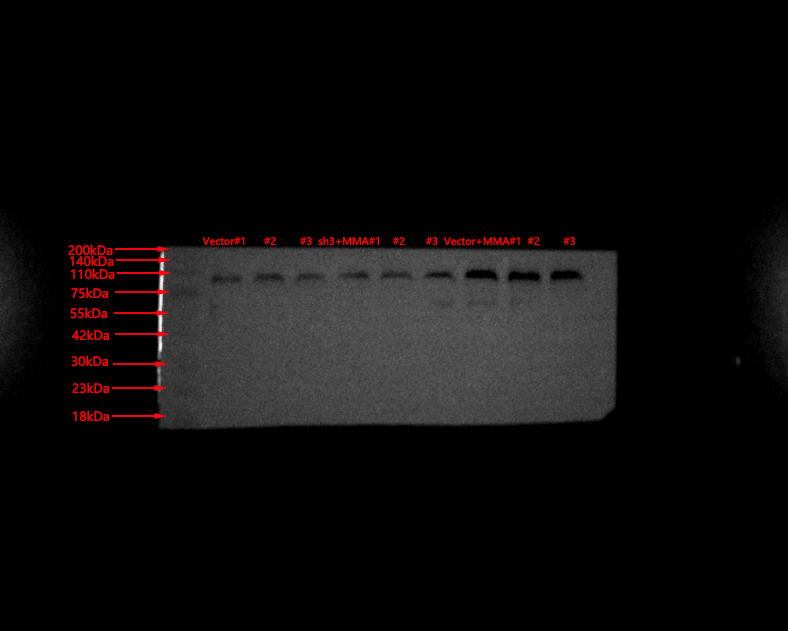


GAPDH


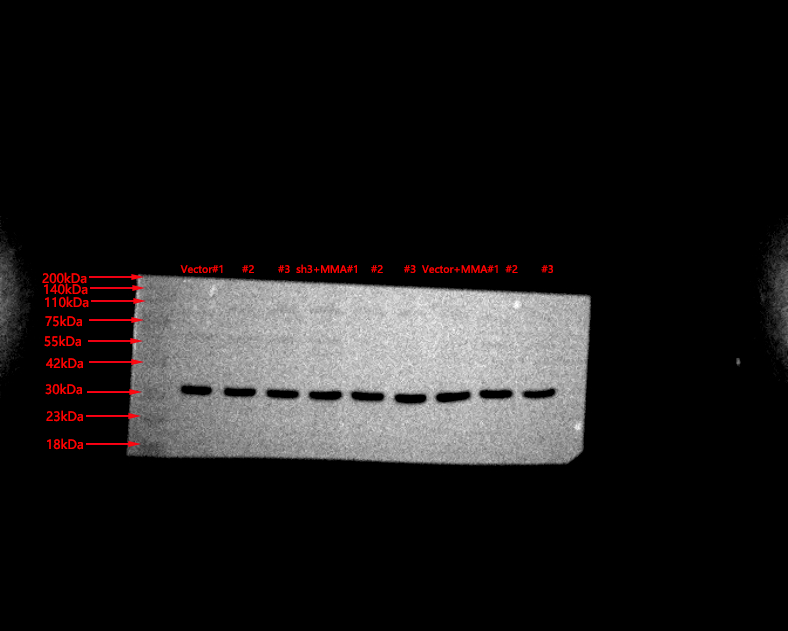


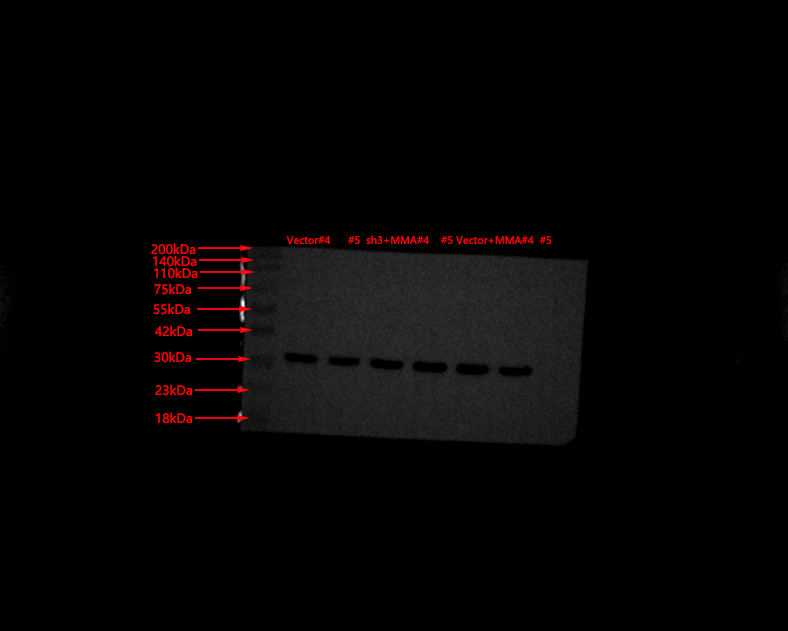


**Figure S8A**

LOXL2


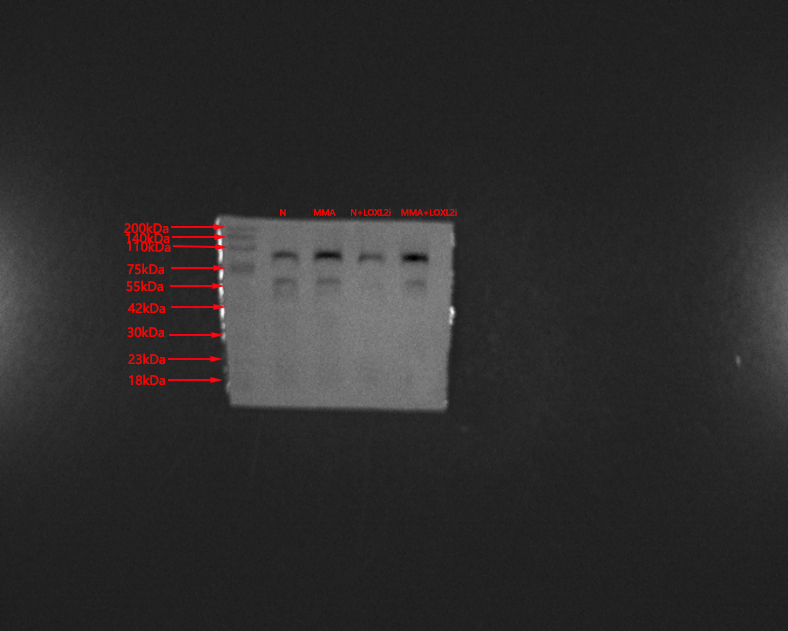


GAPDH


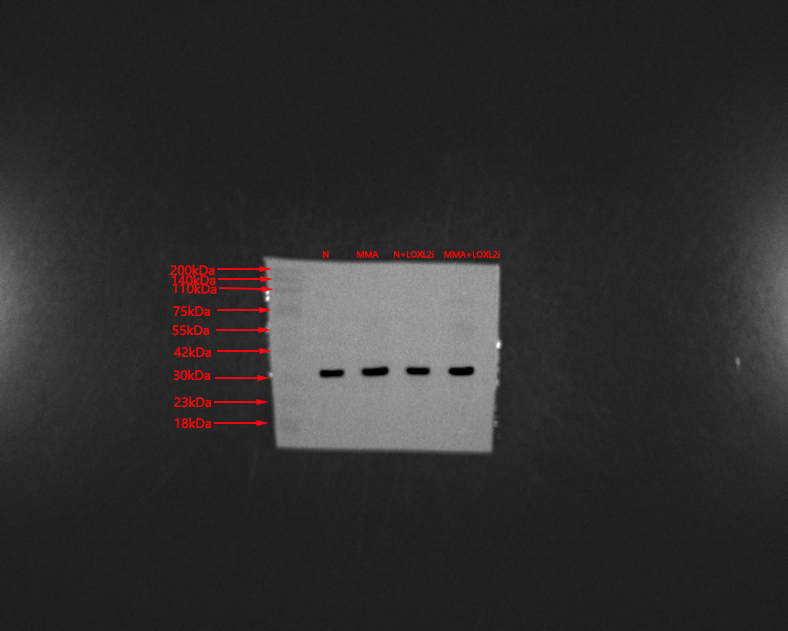

Supplement: Supplementary file 3 — Original Western blots [file 41419_2025_7751_MOESM3_ESM.docx]
